# On Pentafluoroorthotellurates and Related Compounds

**DOI:** 10.1021/acs.chemrev.5c00075

**Published:** 2025-09-16

**Authors:** Julia Bader, Lukas Fischer, Kurt F. Hoffmann, Niklas Limberg, Alexandre Millanvois, Friederike Oesten, Alberto Pérez-Bitrián, Johanna Schlögl, Ahmet N. Toraman, Daniel Wegener, Anja Wiesner, Sebastian Riedel

**Affiliations:** † Department of Chemistry and Biochemistry, 9166 Freie Universität Berlin, Fabeckstr. 34/36, 14195 Berlin, Germany; ‡ Department of Chemistry, 8166 University of British Columbia, 2036 Main Mall, V6T 1Z1 Vancouver, BC, Canada; § Institut für Chemie, 9373 Humboldt-Universität zu Berlin, Brook-Taylor-Straße 2, 12489 Berlin, Germany

## Abstract

This Review surveys the properties and applications of the pentafluoro­orthotellurate (“teflate”, OTeF_5_) ligand and highlights the syntheses of the known teflate-based compounds across the periodic table. Due to the accessibility to several useful teflate transfer reagents and its unique properties, including strong electron-withdrawing character, considerable steric bulk, and stability against oxidation, a variety of intriguing *p*-block and *d*-block species have been reported. These encompass highly reactive Lewis acids, versatile weakly coordinating anions, neutral and cationic noble gas compounds, and a wide number of transition metal complexes. The lower analogues of the pentafluoro­orthochalcogenate group, OSeF_5_ and OSF_5_, are described as well, although fewer examples are known. Recent progress in the derivatization of the OTeF_5_ group to *cis*- and *trans*-PhTeF_4_O or *trans*-(C_6_F_5_)_2_TeF_3_O moieties is also discussed, opening pathways to exciting new research directions.

## Graphical abstract

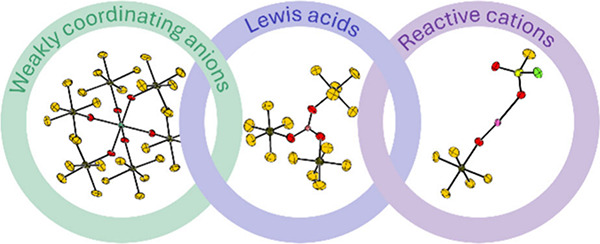

## 1 Introduction

The element fluorine possesses the highest electronegativity among the elements, giving rise to unique properties of its compounds. In fact, almost every element in the periodic table reacts with fluorine, with the exception of the noble gases He and Ne, to form a huge variety of different species. By substituting fluorides in element-fluoride compounds with sterically demanding groups with high electronegativities, some of these properties like melting point, vapor pressure, coordination behavior etc. can be manipulated. Famous examples of these endeavors are perfluorinated aryl, aryloxy, alkyl, and alkyloxy groups or perfluorinated carborates. A highly promising candidate in this regard is the pentafluoro­orthotellurate group (OTeF_5_), also known as the teflate group, which displays similarly strong electron-withdrawing properties when compared to fluorine while having a greater volume and a reduced lone pair back bonding. The rich chemistry of this “big brother” of fluorine across the periodic table is shown in Figure 1.

**Figure 1:**
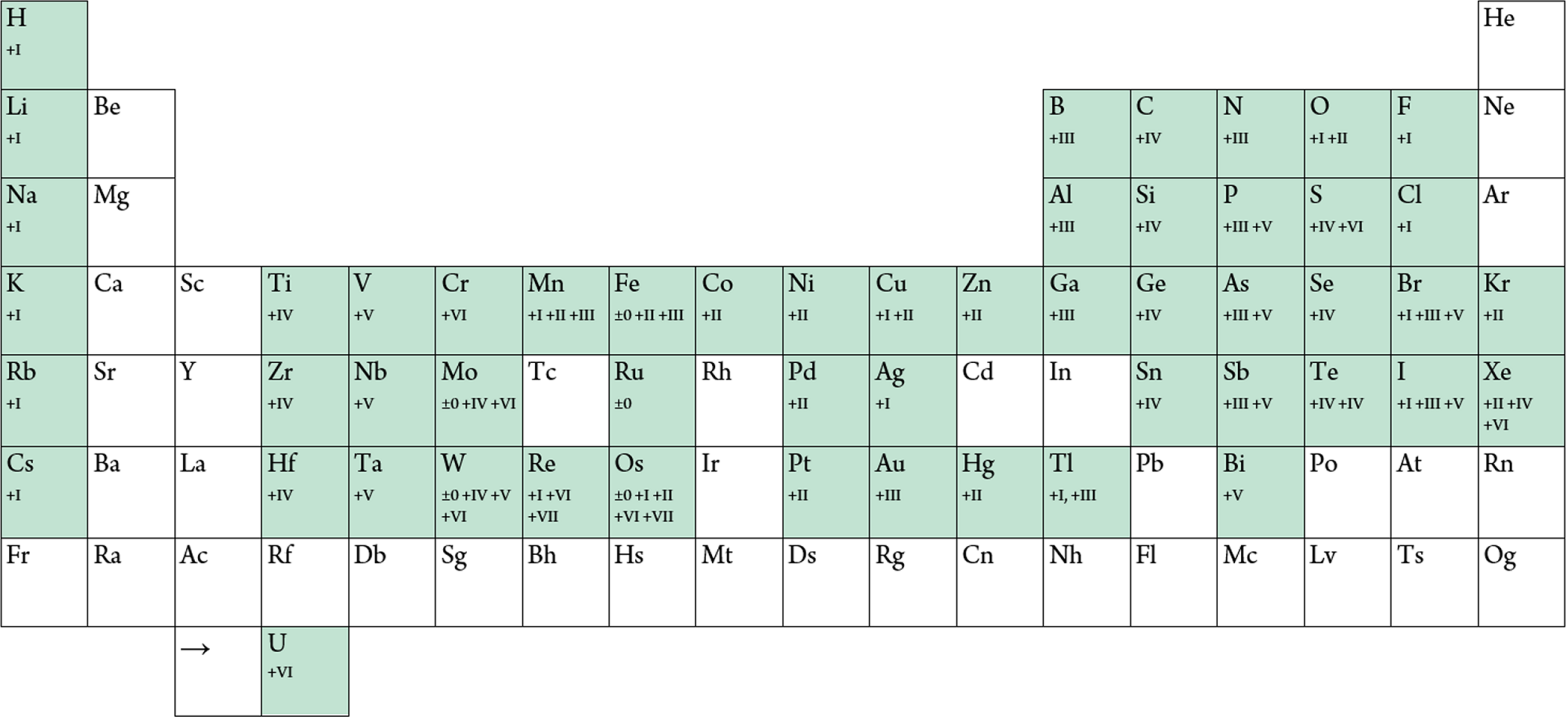
Periodic table of teflate compound elements and their oxidation states.

### 1.1 Scope of the Review

Following up on an initial review by Seppelt from 1982,^1^ this Review aims to provide a comprehensive overview of all hitherto reported teflate compounds across the periodic table of elements. Furthermore, the selenates (OSeF_5_) and sulfonates (OSF_5_) will also be addressed. Starting with a general description of the peculiarities of the OTeF_5_ group, the following sections focus on different element−OTeF_5_ compounds, thereby discussing their syntheses, aspects of their characterization and properties, and their possible applications in preparative chemistry.

## 2 Fundamental Compounds and Their Applications

### 2.1 Pentafluoro­orthotelluric Acid

The pentafluoro­orthotelluric acid HOTeF_5_ (teflic acid) was accidentally discovered in 1964 by Engelbrecht and Sladky when they reacted BaTeO_4_ and fluorosulfonic acid in a molar ratio of 1:10.^2^ The reaction was accompanied by several byproducts, such as F_5_TeOSO_3_H, F_5_TeOSO_2_F, (F_5_TeO)_2_SO_2_, TeF_6_, SiF_4_ (reaction with the glassware) and SO_3_, and therefore the yield of isolated HOTeF_5_ was relatively low.^3^ However, they optimized the reaction conditions and found that the use of barium tellurate with the exact stoichiometric composition BaH_4_TeO_6_ (also written as BaTeO_4_·2H_2_O) increases the yield to 95% (see eq 1).^4^


1 BaTeO4·2H2O+7HSO3F→−20to160°C,3hHOTeF 5+Ba(SO3F)2+5H2SO4

At ambient conditions HOTeF_5_ is a glass-like solid (mp = 40 ± 1 °C, bp = 60 ± 1 °C, ρ = 2.626 g/cm^3^) with a vapor pressure of 53 mbar (40 Torr) at 0 °C and 147 mbar (110 Torr) at 17 °C, making it a convenient reagent that can be easily condensed at room temperature.^2,3^ Still, *caution must be taken when exposing HOTeF_5_ and some of its derivatives to moisture, since contact with water leads to hydrolysis under formation of telluric acid Te­(OH)_6_ and hydrogen fluoride (HF)*.

Several years after its initial discovery, a more convenient route to obtain HOTeF_5_ in good yields (70–85%) was developed by reacting Te­(OH)_6_ and HSO_3_F as shown by eq 2.^5–7^


2 Te(OH)6+5HSO3F→170°C,5to6hHOTeF 5+5H2SO4

The compound HOTeF_5_ is easily characterized by ^19^F NMR spectroscopy due to its resulting AB_4_ spin system (see Figure 2). As expected, the compound reacts as a strong Brønsted acid, and its acidity has been determined to range between those of HCl and HNO_3_.^8^ For a more specific application, the synthesis of the deuterated acid DOTeF_5_ starting from [NBu_4_]​[OTeF_5_] and excess of sulfuric acid-*d*_*2*_ in glassware that is treated with D_2_O and flame-dried beforehand is reported.^7^

### 2.2 Reactive Synthons

Besides the acid HOTeF_5_, typical reactive synthons to introduce the teflate ligand into main group and transition metal compounds are B­(OTeF_5_)_3_, AgOTeF_5_ and Xe­(OTeF_5_)_2_.^9–14^ For B­(OTeF_5_)_3_ and AgOTeF_5_ the reactions can be described as metathesis reactions with the formation of BF_3_ and AgCl, respectively, as driving force, enabling the synthesis of e.g. As­(OTeF_5_)_3_, Sb­(OTeF_5_)_3_, U­(OTeF_5_)_6_, Xe­(OTeF_5_)_4_, and Xe­(OTeF_5_)_6_ (with B­(OTeF_5_)_3_) and Me_3_GeOTeF_5_ (with AgOTeF_5_).^15–20^ In contrast to this, Xe­(OTeF_5_)_2_ introduces OTeF_5_ groups by an oxidative addition leading to compounds in high oxidation states such as As­(OTeF_5_)_5_, I­(OTeF_5_)_5_, or Te­(OTeF_5_)_6_.^15,21,22^ Another effective oxidative transfer reagent is the hypochlorite ClOTeF_5_, which has been applied for the synthesis of [Au­(OTeF_5_)_3_]_2_, Fe­(OTeF_5_)_3_, Ga­(OTeF_5_)_3_, and Tl­(OTeF_5_)_3_.^5,23–27^ The commonly used synthesis of B­(OTeF_5_)_3_, AgOTeF_5_, Xe­(OTeF_5_)_2_ and ClOTeF_5_ as well as their general introduction pathways are summarized in Scheme 1 (see also Sections 5.1, 9.2, 10.2, and 11.1.1).

**Scheme 1:**
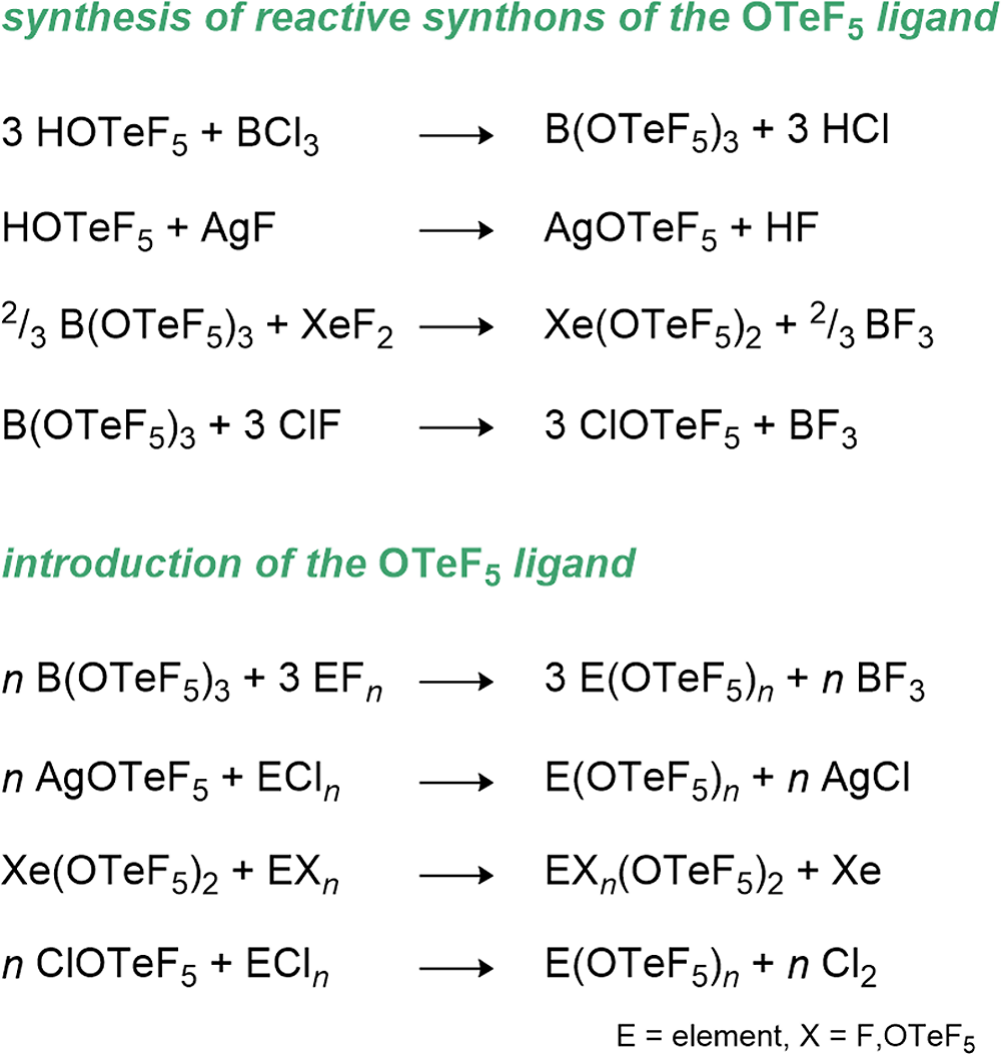
Synthesis and Utilization of the Most Commonly Used OTeF_5_ Transfer Reagents

### 2.3 Properties

The access to unusually high oxidation states in teflate-based compounds,^16,22,28^ that are typically only achieved with fluoride ligands, led to the investigation of the electron-withdrawing properties of the OTeF_5_ group and the comparison to those of the most electronegative element, fluorine. Different methods have been applied such as comparison of the chemical shift differences of the compounds Me-X and Et-X (X = F, Cl, Br, I, OTeF_5_) in ^1^H NMR spectra,^29,30^ the preference of equatorial OTeF_5_ substitution on square pyramidal IF_*n*_(OTeF_5_)_5–*n*_ (*n* = 1–4) and the infrared and multinuclear NMR experiments of OPF_2_X (X = Br, Cl, F or OTeF_5_).^21,31^ Additionally, ^127^I and ^129^Xe Mössbauer together with ^125^Te and ^129^Xe NMR spectroscopy on different teflate and fluoride complexes were carried out for this purpose.^32^ These experimental investigations came to opposite conclusions. A very recent work reinvestigated this topic with a combination of different quantum-chemical methods and concluded that the teflate group exhibits extraordinary electron-withdrawing properties, but less pronounced than in the case of fluorine.^33^ When having a closer look at the QTAIM charges (QTAIM = quantum theory of atoms in molecules) of the different atoms within the OTeF_5_ fragment, it is clear that the bonding within the teflate moiety is significantly ionic. In addition, it was also shown that the OSF_5_, OSeF_5_ and OTeF_5_ group exhibit nearly identical electron-withdrawing properties.

A comparison of the negatively charged OTeF_5_ with the fluoride ligand in terms of electronic properties have been investigated on the homoleptic transition metal teflate complexes [Ni­(OTeF_5_)_4_]^2–^, [Co­(OTeF_5_)_4_]^2–^, [Mn­(OTeF_5_)_4_]^2–^ and [Mn­(OTeF_5_)_5_]^2–^.^34–36^ From SQUID measurements and UV/visible spectra accompanied with quantum-chemical calculations it was deduced that the teflate group is a weak/medium-field ligand and can be included in the spectrochemical series at a similar position as the fluorido ligand.

A striking difference between the teflate and the fluoride ligand is their size. The higher steric demand of the OTeF_5_ group results in a reduced tendency to act as a bridging ligand. This is demonstrated in the case of [Ni­(OTeF_5_)_4_]^2–^, [Mn­(OTeF_5_)_4_]^2–^ or [Mn­(OTeF_5_)_5_]^2–^, which exist in a monomeric form while the [NiF_4_]^2–^, [MnF_4_]^2–^ and [MnF_5_]^2–^ ions built extended structures.^37–39^ Therefore, the typical oligomerization is prevented. In the case of the gold-based teflate compound, [Au­(OTeF_5_)_3_]_2_, dimers are formed in the solid state, whereas AuF_3_ shows a helical polymeric structure.^40,41^

Remarkably, the negative charge of the teflate group is distributed over the whole periphery of the ligand leading to very weak intermolecular interactions. Therefore, neutral teflate compounds such as B­(OTeF_5_)_3_, Ti­(OTeF_5_)_4_, U­(OTeF_5_)_6_ and Xe­(OTeF_5_)_2_ possess a high vapor pressure and are sublimable at moderate temperatures despite their size and high molecular weight.^9,12,17,42^ Combined with its high oxidation stability, these properties render the OTeF_5_ group ideal for the application in weakly coordinating anions (WCAs), as described in Section 2.3.2).^43,44^

Reports discussing the stability of the teflate group toward bases or electrophiles are scarce in the literature. Teflic acid is prone to hydrolysis when dissolved in water.^2^ Tötsch and Sladky conducted a detailed study on the hydrolysis of TeF_6_ and found that the hydrolysis of HOTeF_5_ to *cis*-TeF_4_(OH)_2_ proceeds rapidly.^45^ In contrast, the hydrolysis of *cis*-tetrafluorotelluric acid to *fac*- and *mer*-TeF_3_(OH)_3_ requires several days. For the subsequent hydrolysis step, only the configuration is discussed in the literature without specifying reaction times. Ultimately, TeF­(OH)_5_ readily hydrolyzes or disproportionates to Te­(OH)_6_ and *cis*-TeF_2_(OH)_4_. The reaction mechanism likely involves tellurium in a coordination number of 7.^46–49^

Based on these observations, we generally avoid alcohols as solvents in our work with teflate compounds. From our experience in this field, we have concluded that hydrolysis occurs primarily when excess water is added to the reaction mixture. For instance, the anion [Al­(OTeF_5_)_4_]^−^ forms aqua complexes before decomposition. We have crystallized two unpublished examples: Na­[Al­(H_2_O)_2_(OTeF_5_)_4_] and K_2_[Al­(H_2_O)​(OTeF_5_)_5_].

Other Lewis bases are not known to induce decomposition of the teflate ligand. Only for the related systems TeF_6_, MeOTeF_5_, and (MeO)_2_TeF_4_, the addition of one fluoride (from reactions with [NMe_4_]­F) has been reported, resulting in hepta-coordinated tellurium.^48,49^

The aluminate [Al­(OTeF_5_)_4_]^−^ is unfortunately not resistant to the strongest class of electrophiles^50^—silylium cations. Attempts in our group toward the synthesis of alkylated silylium cations [SiEt_3_]^+^ suggest that they may form at low temperatures but readily react to produce Et_3_SiOTeF_5_.^51^ Other strong electrophiles, such as protons, carbocations, or halonium ions, have been reported as salts of teflate-based WCAs and are discussed in Section 2.3.2.

Another significant observation from our research over recent years is that reducing agents, such as R_*x*_SiH_*y*_ (*x* = 3, 2, 1, 0; *y* = 4 − *x*) compounds or hydrides, tend to reduce the OTeF_5_ group to elemental tellurium. This reductive decomposition pathway represents an important limitation when designing synthetic approaches involving teflate-containing compounds.

The teflate group is readily characterized by ^19^F NMR spectroscopy. The fluorine atom in the *trans* position to the oxygen atom is magnetically inequivalent to the four fluorine atoms in the *cis* position, giving rise to an AB_4_ or A_4_B spin system (see Figure 2). The AB_4_ pattern can appear as a pseudo quintet and pseudo doublet as in the case of AgOTeF_5_, but often the close values of δ­(^19^F_A_) and δ­(^19^F_B_) result in a highly convoluted pattern of higher order as in HOTeF_5_ and B­(OTeF_5_)_3_. It is also possible that the A and B part almost fully coincide as in the spectrum of the [Sb­(OTeF_5_)_6_]^−^ anion. In those cases, the satellites of the ^123^Te and ^125^Te nuclei (0.91 and 7.14% natural abundance) with characteristic ^1^ *J*(F–Te) coupling constants in the range of 2500 to 3700 Hz are more informative. The ^2^ *J*(F_A_-F_B_) coupling constant is usually in the range of 175–190 Hz.

**Figure 2:**
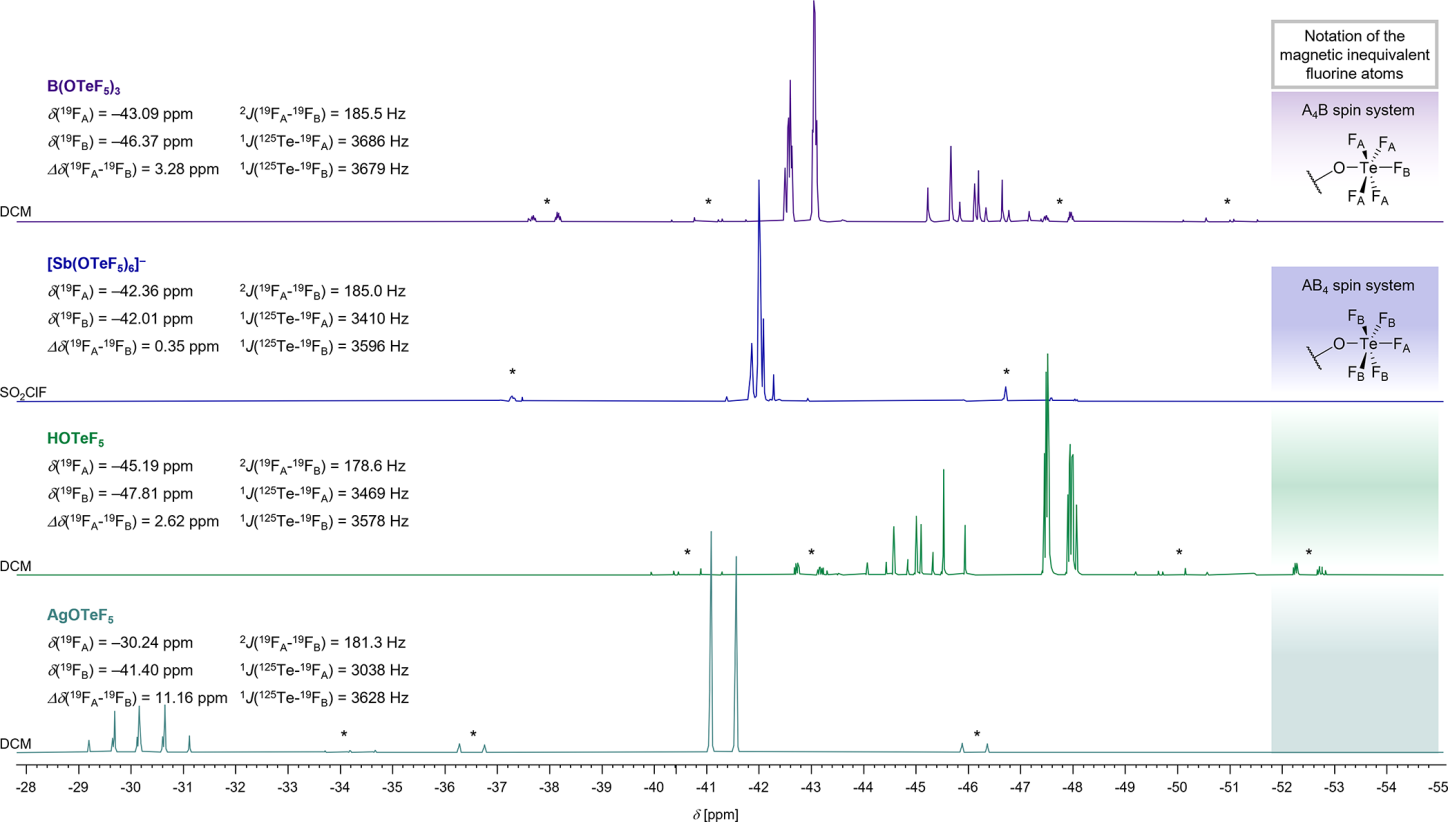
^19^F NMR spectra (377 MHz, r.t.) of B­(OTeF_5_)_3_, [Sb­(OTeF_5_)_6_]^−^, HOTeF_5_ and AgOTeF_5_ illustrating different appearances of the A_4_B or AB_4_ spin systems. The ^125^Te satellites are marked with *.

#### 2.3.1 Lewis Acidity

The high electronegativity of fluorine allows the synthesis of very strong Lewis acids. A well-known example is SbF_5_, which is regarded as one of the strongest conventional Lewis acids. One of the most commonly used methods to measure the Lewis acidity of a given compound is the so-called fluoride ion affinity (FIA). This affinity is defined as the negative reaction enthalpy of the adduct formation of a given Lewis acid and a fluoride ion and can be easily obtained by quantum-chemical calculation.^52,53^ Other typical experimental methods for estimating Lewis acidity are the Gutmann–Beckett method or the formation of e.g. nitrile adducts. For more details on these techniques along with an overview of known Lewis superacids we refer to a recently published review article about this topic.^54^

The substitution of fluoride ligands with bulkier electron-withdrawing groups is an effective strategy to obtain even stronger Lewis acids. This becomes most evident when comparing the corresponding FIA values (see Figure 3). The Lewis acidity of element halides is usually outperformed by oxygen-bound Lewis acids such as triflate, perfluoro-*tert*-butyl alkoxide or teflate compounds. Triflate-based Lewis acids can suffer from bidentate binding of the SO_3_CF_3_ group toward the central atom which explains comparably low FIA values in the case of aluminum and antimony compounds.^52^

Another significant factor for these high Lewis acidities, besides the electron-withdrawing properties of the teflate group, is the reduced lone pair back bonding of the O atoms of the teflate group toward the electron deficient central element, in contrast to direct element-fluorine bonds. Still, the OTeF_5_ ligand, like all oxygen-bound ligands, can delocalize electron density from the free electron pairs at the oxygen atom to the central element.

Several Lewis acids based on teflates have been already reported, such as B­(OTeF_5_)_3_, As­(OTeF_5_)_5_ and more recently, the compounds Al­(OTeF_5_)_3_ and Ga­(OTeF_5_)_3_, which possess exceptionally high FIA values.^26,55^ In the solid state, the Al and Ga teflate have a dimeric structure, which can be easily converted into soluble solvent adducts by the addition of appropriate donor solvents.^26,56^ Depending on the donor solvent, its Lewis acidity will, of course, be lowered.

The compound Sb­(OTeF_5_)_5_ is assumed to be one of the strongest possible neutral Lewis acids, but its synthetic preparation as a neat species or as a solvent adduct remains still elusive.^15,18^ For the lower homologue, the 5-fold coordinated As­(OTeF_5_)_5_, several procedures were reported in literature and it is expected to show a remarkable acidity.^15,52,57^ The potential of this compound is still underexplored, since only one report shows the usage of As­(OTeF_5_)_5_ as a reactant for the synthesis of the arsenium cations [AsCl_4_]^+^ and [AsBr_4_]^+^.^58^ Analogous strong Lewis acids can also be prepared by combining teflates with transition metals, as it was shown by the synthesis of [Au­(OTeF_5_)_3_]_2_. While its first synthesis was already reported in 1985,^40^ a recent publication demonstrates an alternative synthetic approach and is more focused on the Lewis acidic properties of this species.^24^

#### 2.3.2 Weakly Coordinating Anions with Teflate Groups

Due to the low polarizability of fluorine and based on its small size combined with the high electronegativity, anions with fluorine atoms at the periphery of the molecule are excellent candidates for the formation of weakly coordinating anions. Several review articles on that topic have been published in the past,^59–62^ but the teflates only played a minor role, possibly due to their comparably complex synthesis and their expensive starting materials. Still, teflate-based WCAs are of scientific interest due to their high stability toward strong oxidizers. In the 1980s and 1990s, several tetra- and hexacoordinate anions of group 5, 13, and 15 elements have been investigated, namely [B­(OTeF_5_)_4_]^−^ and [M­(OTeF_5_)_6_]^−^ (with M = As, Nb, Sb, Ta, and Bi).^44,57,63–65^ More recently the heavier analogues of the borate ion, [Al­(OTeF_5_)_4_]^−^ and [Ga­(OTeF_5_)_4_]^−^ have been reported.^55,66^ Especially the [Al­(OTeF_5_)_4_]^−^ and [Sb­(OTeF_5_)_6_]^−^ anions have been used to stabilize strong oxidizing cations such as the xenonium ions [Xe­(OTeF_5_)​(SO_2_ClF)]^+^, [Xe­(OTeF_5_)​(C_5_F_5_N)]^+^, and [Xe­(OTeF_5_)​(C_5_H_3_F_2_N)]^+^ or carbocations like [CCl_3_]^+^, [CBr_3_]^+^, [CFCl_2_]^+^ [CFBr_2_]^+^, [C­(OTeF_5_)_3_]^+^, [CBr­(OTeF_5_)_2_]^+^, and [C­(C_6_F_5_)_3_]^+^.^43,67–71^ Furthermore, the aluminate has been utilized for the formation of different reactive cations such as [C_6_H_7_]^+^, [C_9_H_13_]^+^, [P_4_H]^+^, [NO]^+^, [NOP_4_]^+^, [ClMe_2_]^+^, [(CF_3_CH_2_)_2_Cl]^+^, [C_5_F_5_NH]^+^, [C_5_F_4_ClNH]^+^, [(C_5_F_5_N)_2_H]^+^, and [(C_5_Cl_5_N)_2_H]^+^.^51,55,72–75^ The examples of protonated arenes and pyridines demonstrate the robustness of the anion against the smallest electrophile, the proton. Labile cationic complexes, such as the acetylene, ethene or P_4_ complexes of silver­(I) ions^76–78^ or the recently reported transition metal carbonyl cations,^79^ have not been realized so far with teflate-based WCAs. Still, teflates possess a well delocalized negative charge over the 20 or 30 fluorine atoms and could be useful in this regard. A common method to visualize the coordination ability of WCAs is the depiction of the calculated electrostatic potential (ESP) plotted on the electron density. In Figure 4, the more negatively polarized regions in the fluorides ([BF_4_]^−^, [AlF_4_]^−^ and [SbF_6_]^−^) are compared with the teflate-based anions, where the charge delocalization is clearly distinguishable. Within the group of teflates the negatively polarized areas decrease in the row [B­(OTeF_5_)_4_]^−^ > [Al­(OTeF_5_)_4_]^−^ > [Sb­(OTeF_5_)_6_]^−^, indicated by the change of orange and yellow areas to more yellow and green areas at the fluorine atoms.

**Figure 3:**
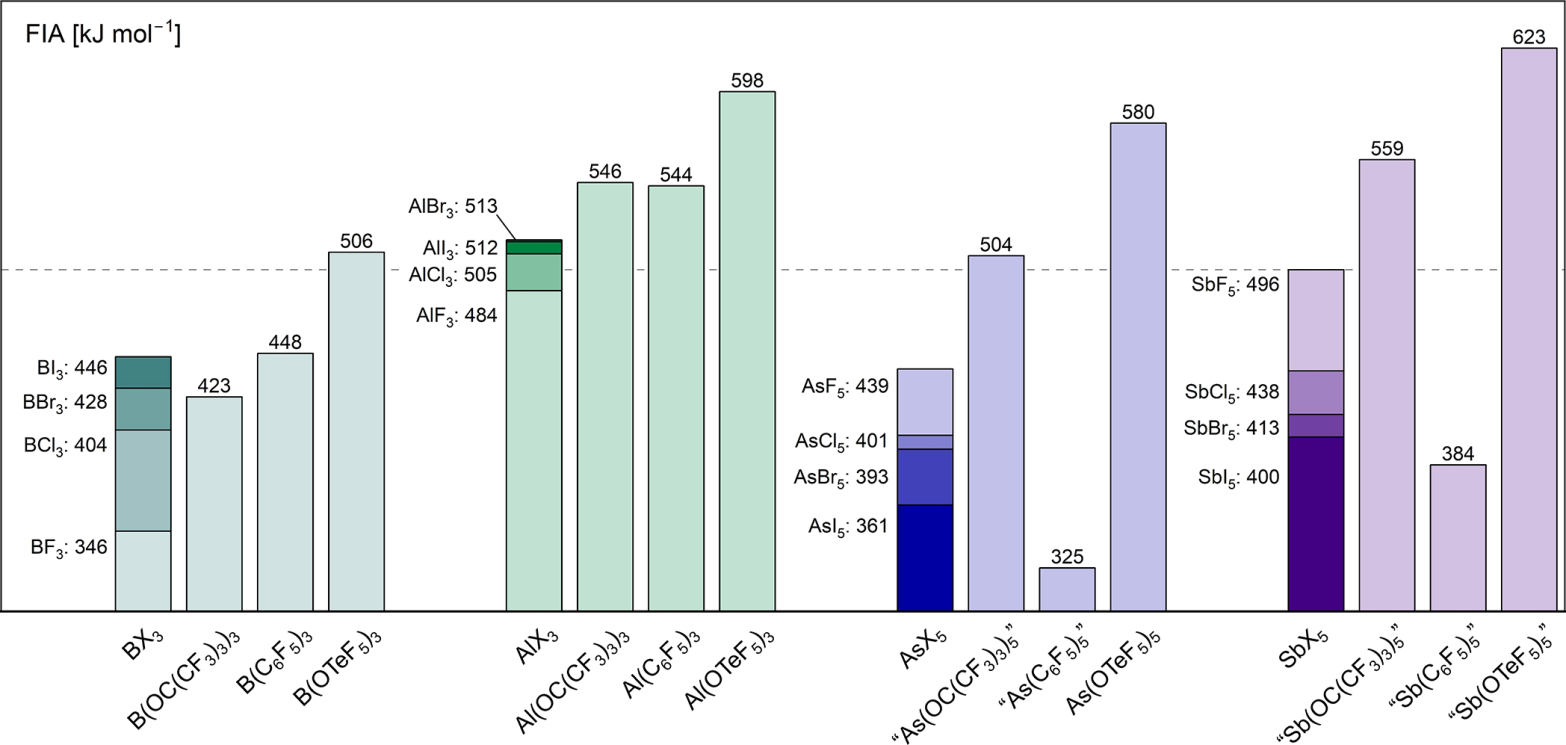
Comparison of FIA values^52^ of monomeric boron, aluminum, arsenic and antimony compounds. Substances in quotation marks are not synthetically available. The gray dashed line indicates the FIA value of SbF_5_ and therefore the threshold for Lewis superacidity.

**Figure 4:**
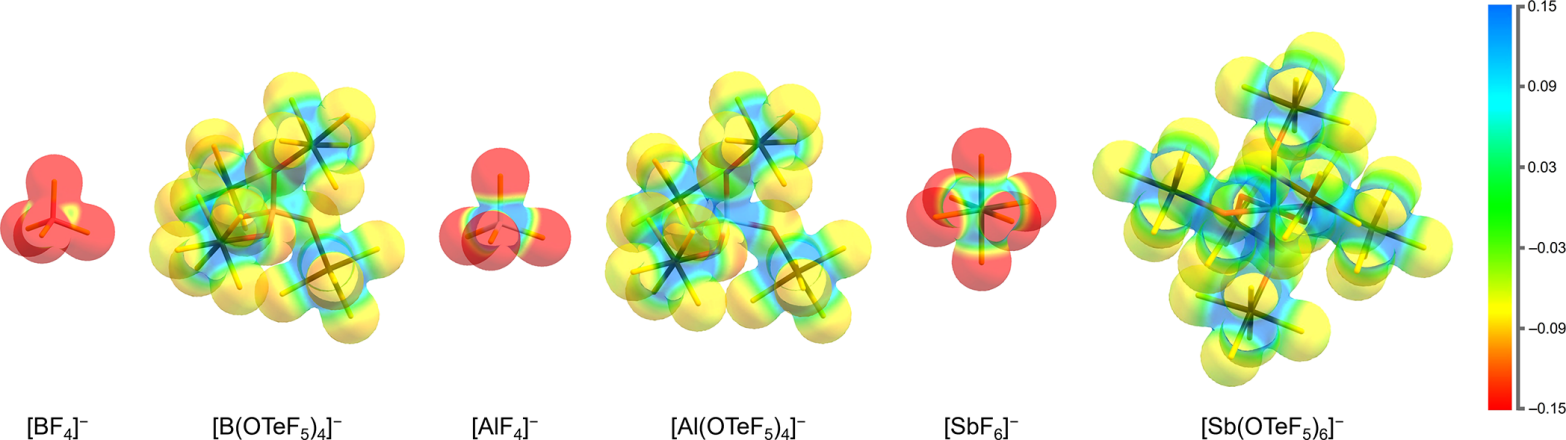
Computed electrostatic potentials plotted on the electron density (BP86/def2-SVP level, isovalue: 0.025 e bohr^–3^) using ORCA 5.0.4 ^80^ visualized with Chemcraft.^81^

#### 2.3.3 Ligand Properties

The recent synthesis of homoleptic 3d metal complexes based on Ni and Co has made it possible to further understand the properties of the teflate ligand in coordination chemistry, proving that it behaves like the fluoride also in ligand-field terms.^34,36^ In fact, [NEt_4_]_2_[Ni­(OTeF_5_)_4_] and [NEt_4_]_2_[Co­(OTeF_5_)_4_] represent analogues of the polymeric [MF_4_]^2–^ species (M = Ni, Co) but with monomeric structures. Through a combination of magnetic and electronic spectroscopy measurements on [NEt_4_]_2_[Ni­(OTeF_5_)_4_], the ligand-field properties of the teflate ligand indicate a weak/medium-field character. Additionally, the inferred value for the ligand-field parameter allowed it to be included in the spectrochemical series at a similar position as the fluoride ion and next to the fluorinated O-donor ligands [OC_4_F_9_]^−^ and [OC_6_F_5_]^−^.^34,82^ In the nephelauxetic series of ligands a Racah parameter of *B* = 896 cm^–1^ of the OTeF_5_ group positions it before the heavier halides and again next to [OC_4_F_9_]^−^ and [OC_6_F_5_]^−^ ligands. In the case of the [Co­(OTeF_5_)_4_]^2–^ anion, a comparative bonding study with the hypothetical monomeric [CoF_4_]^2–^ anion through the interacting quantum atoms (IQA) energy decomposition scheme^83^ demonstrated a similar strength in the Co–X (X = OTeF_5_, F) bonds.^36^ This reinforces the similar electron-withdrawing properties of both groups.

### 2.4 Selenium-Based Derivatives

Already in 1959 the syntheses of the pentafluoroselenium hypofluorite FOSeF_5_ and the peroxide F_5_SeOOSeF_5_ starting from the fluorination of SeO_2_ or SeOCl_2_ were reported.^84^ The reaction is accompanied by the formation of SeOF_2_ and SeF_6_. A more selective reaction uses Hg­(OSeF_5_)_2_ and fluorine in different molar ratios.^85^ In case of a molar ratio of Hg­(OSeF_5_)_2_:F_2_ = 1:1, the corresponding peroxide is formed. If the amount of fluorine is further increased to a ratio of 1:2.6, the hypofluorite can be formed. The mercury­(II) pentafluoro­orthoselenate is also a useful starting material for the introduction of OSeF_5_ ligands.^18,23,85–87^ It is slowly formed by the reaction of HOSeF_5_ with HgF_2_ ^88^ and can be used for the synthesis of As­(OSeF_5_)_3_,^18^ CH_*n*_(OSeF_5_)_*4‑n*_ (*n* = 0, 1, 3),^86^ ClOSeF_5_,^85^ F_5_SeOCN,^89^ F_2_Se­(OSeF_5_)_2_,^87^ F_5_SeO-COCF_3_,^90^ F_5_SeOOSeF_5_,^85^ NO_2_(OSeF_5_),^23^ POF_2_(OSeF_5_),^18^ OSe­(OSeF_5_)_2_,^23^ CrO_2_(OSeF_5_)_2_ ^42^ and VO­(OSeF_5_)_3_.^42^ The corresponding acid HOSeF_5_ is another synthetically very useful synthon. Three different preparation routes have been reported.^91–93^ The most favorable is the reaction of SeO_2_F_2_ with HF. The pentafluoro­orthoselenic acid was used to synthesize e.g. the alkaline metal salts MOSeF_5_ (M = Li, Na, K, Rb, Cs), the silane Me_3_SiOSeF_5_, the xenon compound Xe­(OSeF_5_)_2_ as well as the above-mentioned Hg­(OSeF_5_)_2_ compound.^88,94,95^

Unfortunately, the selenium compounds in oxidation state +VI are not as stable as the tellurium-based compounds, due to the *d*-block contraction caused by the additional 3d^10^ electrons.^96^ Therefore, the selenium of the OSeF_5_ group is easily reduced to the oxidation state +IV and acts as a better oxidizer.^1^ For instance, HOSeF_5_ readily oxidizes alkali chlorides^1,88,93^ and HCl^94^ to chlorine, is observed to attack glass and metal apparatuses^93^ and thermally decomposes^91^ under the formation of selenium tetrafluoride. Chlorine formation is also reported when Hg­(OSeF_5_)_2_ is reacted with TiCl_4_ and WCl_6_.^42^ The desired substitution products Ti­(OSeF_5_)_4_ and W­(OSeF_5_)_6_ are not stable and undergo a formal elimination of SeOF_4_. The pentavalent compound dimerizes above −100 °C to diselenium dioxide octafluoride Se_2_O_2_F_8_.^94,97^ The colorless liquid Se_2_O_2_F_8_ is also formed in the synthetic attempts toward Se­(OSeF_5_)_4_ ^87^ from SeCl_4_ and Hg­(OSeF_5_)_2_ or during storage of the instable As­(OSeF_5_)_3_.^18^ In contrast, the corresponding teflate species As­(OTeF_5_)_3_, Ti­(OTeF_5_)_4_ and W­(OTeF_5_)_6_, except for Se­(OTeF_5_)_4_, are synthetically available. Since fundamental starting materials for the introduction of the OSeF_5_ group are either too reactive (HOSeF_5_) or not accessible (B­(OSeF_5_)_3_ or AgOSeF_5_), only a limited number of OSeF_5_-based compounds have been realized and are summarized in Table 1.

**Table 1:** Overview of OSeF_5_ Compounds

Group	Compound	Refs
1	HOSeF_5_	^91–93^
	MOSeF_5_ (M = Li, Na, K, Rb, Cs)	^87,88^

14	CH_3_OSeF_5_	^21,86^
	CH_2_(OSeF_5_)_2_	^86^
	CH(OSeF_5_)_3_	^86^
	C(OSeF_5_)_4_	^86,87^
	F_5_SeO-COCl	^86^
	F_5_SeOCN	^87,89^
	F_5_SeO-COCF_3_	^90^
	C_5_F_9_OSeF_5_	^98^
	F_5_SeO-COF	^98^
	Me_3_SiOSeF_5_	^94^

15	NH_4_[OSeF_5_]	^88^
	[NO_2_]​[OSeF_5_]/NO_2_(OSeF_5_)	^23^
	POF_2_(OSeF_5_)	^18^
	As(OSeF_5_)_3_	^18^

16	F_5_SeOSeF_5_	^85,87,98–101^
	F_5_SeOOSeF_5_	^84,85,87,99^
	F_5_SeO-SO_2_-OSO_2_F	^90^
	F_5_SeOSF_5_	^98^
	F_5_SeO-SO_2_F	^99^
	*cis*-BrCH_2_-SF_4_-OSeF_5_	^102^
	F_2_Se(OSeF_5_)_2_	^87^
	OSe(OSeF_5_)_2_	^23,87^

17	FOSeF_5_	^84,85,87,98^
	ClOSeF_5_	^85^
	BrOSeF_5_	^85^
	Br(OSeF_5_)_3_	^85^
	Rb[Br(OSeF_5_)_4_]	^23^
	I(OSeF_5_)_3_	^85^
	F_*n*_I(OSeF_5_)_5‑*n*_	^21^

18	Xe(OSeF_5_)_2_	^87,103–107^
	XeF(OSeF_5_)	^103,106,107^
	[Xe(OSeF_5_)]​[AsF_6_]	^106^
	Xe(OSeF_5_)​(OTeF_5_)	^5,107^

transition metal	VO(OSeF_5_)_3_	^42^
	CrO_2_(OSeF_5_)_2_	^42^
	[Mn(CO)_5_(OSeF_5_)]	^108^
	[Re(CO)_5_(OSeF_5_)]	^108^
	Hg(OSeF_5_)_2_	^87,88^

### 2.5 Sulfur-Based Derivatives

In contrast to the OTeF_5_ and OSeF_5_ groups, most of the reported OSF_5_ compounds can be found bearing an organic backbone. This moiety is found as alkyl and aryl derivatives and these are mainly obtained from highly reactive reagents prepared from scarcely available thionyl tetrafluoride (SOF_4_).^109–110^ [Bibr newref111] To prepare these alkyl derivatives, hypochlorite (ClOSF_5_) and hypofluorite (FOSF_5_) are used and are respectively obtained from direct treatment of chlorine monofluoride (ClF) and fluorine with SOF_4_ (see Scheme 2A).^111,112^ With these reagents in hand, an addition on halogenated alkenes can be performed.^113–116^ In the case of aryl-OSF_5_ species, the first reported procedure required the pentafluoro­oxosulfate peroxide F_5_SOOSF_5_ (see Scheme 2B),^117^ which can be prepared via several methods like the reaction of FOSF_5_ with SOF_4_ or SOF_2_ or by photolysis of FOSF_5_.^118,119^ In 2021, Haupt and co-workers explored the reactivity of the pentafluoro­oxosulfate silver salt (AgOSF_5_) that was synthesized from AgF and SOF_4_.^110^ The silver salt is only stable in MeCN solution and was utilized for the formation of three first-row transition metal complexes bearing the OSF_5_ ligand, namely, [(bpy)_2_Ni­(OSF_5_)_2_] and [(bpy)_2_Cu­(OSF_5_)_*n*_] (*n* = 1, 2; bpy = 2,2′-bipyridine) through a metathesis reaction. Furthermore, AgOSF_5_ was reported to react with halides to CuOSF_5_, [NEt_3_Me]​[OSF_5_], [PPh_4_]​[OSF_5_] and [PNP]​[OSF_5_] (PNP = bis­(triphenylphosphine)­iminium) again via metathesis reactions.^109^ The poor nucleophilicity of the [OSF_5_]^−^ anion was demonstrated with an inefficient addition (see Scheme 2C) at an *in situ* generated aryne (12% yield).^110^ Recently, a solution of AgOSF_5_ in MeCN was synthesized from commercially available SOCl_2_, AgF_2_ and [NEt_4_]­Cl (ratio SOCl_2_:AgF_2_:[NEt_4_]Cl = 1:16:2) and reacted with primary amines to sulfurimidoyl fluorides.^120^ The excess of AgF_2_ was reduced when AgF instead of tetraethylammonium chloride was added to the reaction mixture (ratio SOCl_2_:AgF_2_:AgF = 1:2.5:1.2).^121^ Then, the pentafluoro­oxosulfate anion was reacted with dibenzobromolium and dibenzochlorolium salts to yield a set of new aryl-OSF_5_ species (see Scheme 2D). The lipophilicity of those compounds was investigated through reverse phase HPLC (HPLC = high-performance liquid chromatography) and gave the following order of lipophilicity when compared to related fluorinated derivatives: SCF_3_ > OSF_5_ > OCF_3_ ≈ SF_5_ > CF_3_. Finally, Pitts et al. used the acetonitrile solution of AgOSF_5_ to functionalize cyclobutanes with an OSF_5_ group (see Scheme 2E) and discussed the products’ potential as bioisosteres of *para*-substituted benzene rings.^122^

**Scheme 2:**
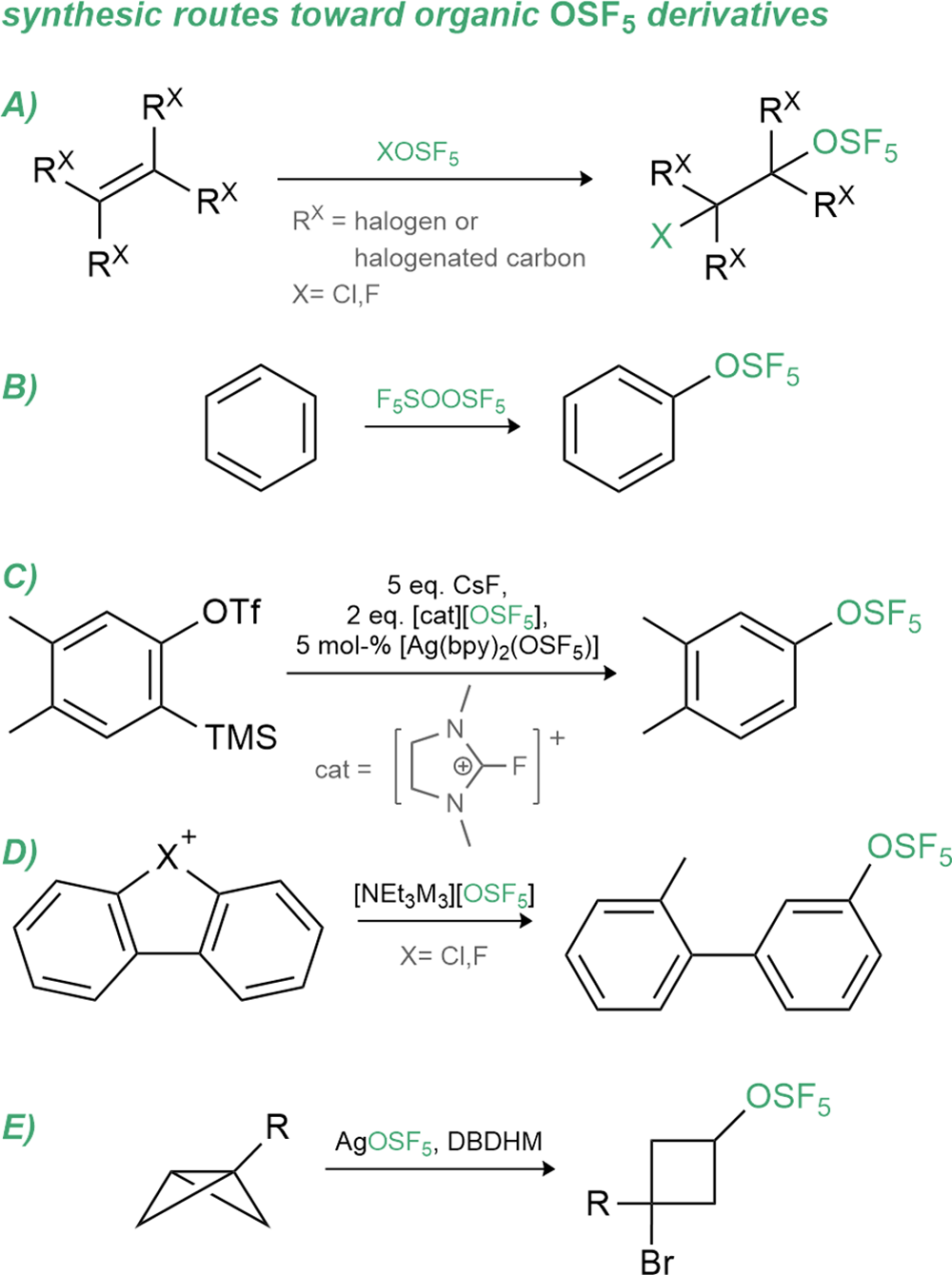
Overview of Synthetic Routes toward Organic OSF_5_ Species (OTf = triflate, DBDHM = 1,3-dibromo-5,5-dimethylhydantoin)

Until now, the limited availability of starting materials has prevented chemists from the synthesis of OSF_5_-based compounds. Besides the comparably new synthon AgOSF_5_ (in acetonitrile solutions), only the peroxide (OSF_5_)_2_,^118^ the fluoro­sulfonyl FSO_3_SF_5_,[Bibr newref123] and the hypofluorite FOSF_5_ ^123^ are found to be handleable reactants. The Brønsted acid HOSF_5_ already decomposes at −78 °C to HF and SOF_4_.^124^ Metal salts of the [OSF_5_]^−^ anion are prone to metal fluoride formation and SOF_4_ elimination.^125^ The hypochlorite ClOSF_5_ is only stable for a limited amount of time even stored at lower temperature (−40 °C) and decomposes to its starting materials ClF and SOF_4_.

Due to the few examples of OSF_5_-bearing compounds, little is known about their toxicity. Only in the case of the oxide F_5_SOSF_5_ a provisional operational limit of 0.5 ppm was discussed.^126^

### 2.6 Derivatives of the Teflate Group

Despite the advantageous properties of the pentafluoro­orthotellurate group that allowed the synthesis of a variety of different species presented in this review, the teflate ligand unfortunately shows some drawbacks. These include the occasionally observed sensitivity to hydrolysis leading to the formation of decomposition products (e.g., HF), as well as the necessity of special starting materials (Te­(OH)_6_ and HSO_3_F) for the synthesis of the most commonly used teflate source HOTeF_5_.^1,7^

Consequently, a modification of the teflate ligand and in particular the substitution of some of the fluorine atoms of the OTeF_5_ unit by (perfluoro)­aryl groups was sought. The reactivity of the phenyltellurium­(VI) compound PhTeF_5_ and its derivatives *trans*-Ph_2_TeF_4_ and *mer*-Ph_3_TeF_3_ toward alcohols, amines and silicon reagents had been already studied in 1989.^127^ However, the obtained teflate derivatives have never been employed, most likely due to the inconvenient synthesis of the starting material PhTeF_5_ and its derivatives at that time.^128–131^ In 2019 an easy-to-handle system for the oxidative fluorination of diaryltellurides consisting of trichloroisocyanuric acid (TCICA), KF and trifluoroacetic acid was reported by Togni, Pitts et al.^132,133^ This system made the synthesis of a broad scope of TeF_5_-substituted arenes, including PhTeF_5_, attainable. The latter was found to be air-stable, but hydrolyzes quantitatively to the *cis*-PhTeF_4_OH compound in an acetonitrile/water mixture,^132^ and therefore entails the first organotellurium derivative of HOTeF_5_, which was easily accessible.

This outcome was used as starting point for the synthesis of two different sets of air-stable aryl derivatives of the teflate group. The preparation of *cis*-PhTeF_4_OH was improved and further transformed into the corresponding silver salt, which could be used as a *cis*-PhTeF_4_O transfer reagent in its reaction with [PPh_4_]Cl (see Scheme 3A).^134^

**Scheme 3:**
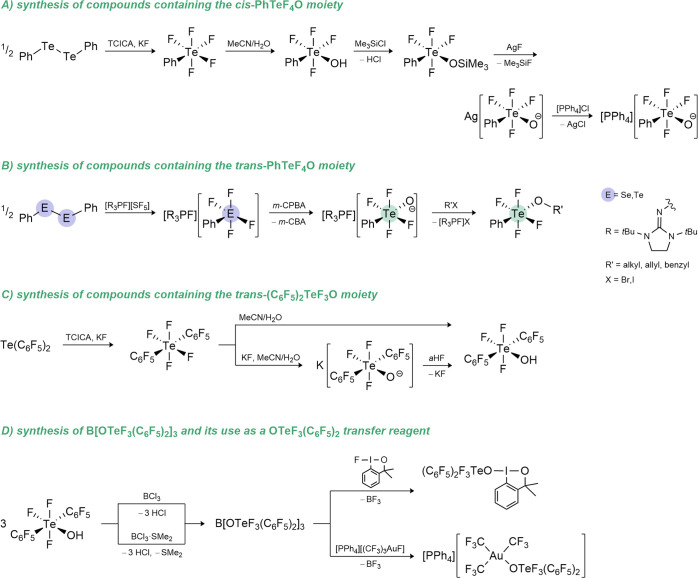
Overview of the Synthetic Routes toward Organic Derivatives of the OTeF_5_ Group and Reactivity of B­(OTeF_3_(C_6_F_5_)_2_)_3_

An approach for the synthesis of the *trans*-PhTeF_4_O moiety was made by Dielmann et al. with the use of an oxidative fluorination of diphenylditelluride by a [SF_5_]^−^ salt to yield the elusive [PhTeF_4_]^−^ anion. Further oxidation of this species led to the formation of the *trans*-[PhTeF_4_O]^−^ anion, which could be subsequently alkylated to *trans*-PhTeF_4_OR (R = alkyl, allyl, benzyl) (see Scheme 3B).^135^

Furthermore, the synthesis of a perfluorinated derivative *trans*-(C_6_F_5_)_2_TeF_3_OH was achieved by a selective hydrolysis of *trans*-(C_6_F_5_)_2_TeF_4_, which can be prepared through fluorination of (C_6_F_5_)_2_Te using the TCICA/KF oxidation system, as shown in Scheme 3C. Quantum-chemical calculations revealed a higher Brønsted acidity and robustness against fluoride abstraction of the *trans*-(C_6_F_5_)_2_TeF_3_OH compared to the phenyl analogue.

The teflic acid derivative *trans*-(C_6_F_5_)_2_TeF_3_OH (*trans*-(C_6_F_5_)_2_TeF_3_O moiety simplified as OTeF_3_(C_6_F_5_)_2_) was then applied for the syntheses of B­(OTeF_3_(C_6_F_5_)_2_)_3_ and Al­(OTeF_3_(C_6_F_5_)_2_)_3_.[Bibr newref138] The boron Lewis acid was realized by reacting HOTeF_3_(C_6_F_5_)_2_ with BCl_3_ or BCl_3_·SMe_2_ (see Scheme 3D).^136^ This Lewis acid has a high thermal stability up to 300 °C and one of the most sterically encumbered boron centers known in the literature (see Figure 5). The Lewis acidity of this compound, evaluated by theoretical (fluoride ion affinity and global electrophilicity index) and experimental (Gutmann–Beckett, ν­(CN)) methods, was found to be comparable to that of B­(C_6_F_5_)_3_ and slightly lower than that of the related B­(OTeF_5_)_3_. The affinity of B­(OTeF_3_(C_6_F_5_)_2_)_3_ toward pyridine was evaluated using isothermal titration calorimetry (ITC), which confirmed the above-mentioned trend in Lewis acidity. Inspired by the reactivity of B­(OTeF_5_)_3_, the use of B­(OTeF_3_(C_6_F_5_)_2_)_3_ as a ligand-transfer reagent was first demonstrated by the reaction with [NMe_4_]F to form the ammonium salt [NMe_4_]​[OTeF_3_(C_6_F_5_)_2_]. Starting from the corresponding fluoro-compounds and following a similar procedure, the boron Lewis acid was then used in the synthesis of the Au­(III) species [PPh_4_]​[(CF_3_)_3_Au­(OTeF_3_(C_6_F_5_)_2_)], and furthermore in the preparation of a Togni-type hypervalent iodine compound (see Scheme 3D). Teflate-based derivatives and their compounds are gathered in Table 2.

**Figure 5:**
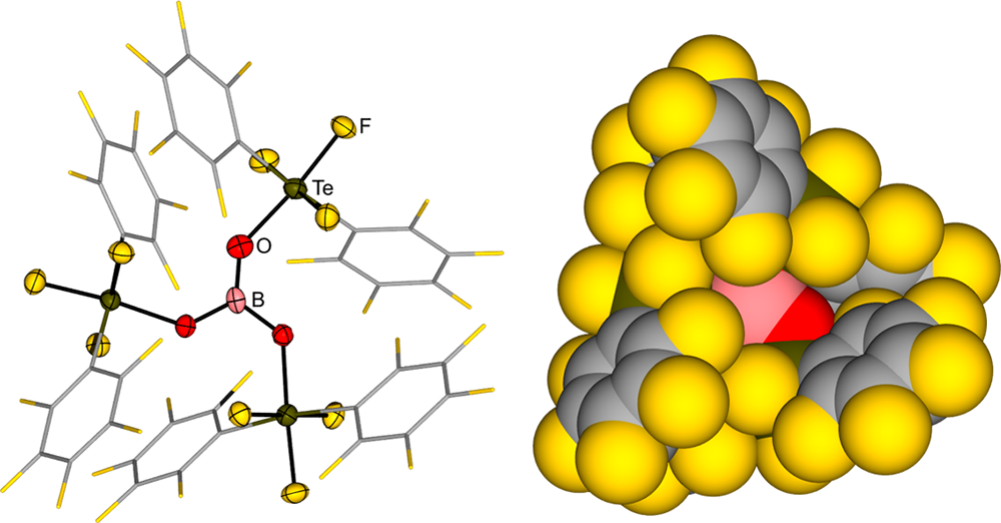
Molecular structure (left, perfluorophenyl substituents are shown as wireframes) and space-filling model (right) of B­(OTeF_3_(C_6_F_5_)_2_)_3_.^136^

**Table 2:** Summary of Teflate-Based Derivatives

Compound	Refs
PhTeF_4_X, Ph_2_TeF_3_X, Ph_3_TeF_2_X (X = OMe, NMe_2_, NEt_2_)	^127^
[cat]​[*trans*-PhTeF_4_O] (cat = Ag, PPh_4_)	^135^
*trans*-PhTeF_4_OR (R = alkyl, allyl, benzyl)	^135^
PhTeF_5_, *cis*-PhTeF_4_OH	^132–134^
*trans*-(C_6_F_5_)_2_TeF_3_OH	^134^
[PPh_4_]​[*cis*-PhTeF_4_O]	^134^
K[OTeF_3_(C_6_F_5_)_2_]	^134^
[NMe_4_]​[OTeF_3_(C_6_F_5_)_2_]	^136^
[PPh_4_]​[OTeF_3_(C_6_F_5_)_2_]	^136^
B(OTeF_3_(C_6_F_5_)_2_)_3_	^136^
Al(OTeF_3_(C_6_F_5_)_2_)_3_	[Bibr newref138]
[PPh_4_]​[(F_3_C)_3_AuOTeF_3_(C_6_F_5_)_2_]	^136^
C_9_H_10_OIOTeF_3_(C_6_F_5_)_2_	^136^

## 3 Group 1 Teflates

Alkali metal teflates MOTeF_5_ (M = Li, Na, K, Rb, Cs) can all be prepared in a similar manner following a method introduced by Sladky et al.^11,137,138^ This reaction relies on the condensation of a 10-fold excess of HOTeF_5_ onto the respective alkali metal chloride MCl and warming the reaction mixture to room temperature, whereby evolution of HCl gas takes place (see eq 3). After removal of the excess of acid under reduced pressure white, crystalline solids are obtained.


3 MCl+10HOTeF 5→−9HOTeF 5MOTeF 5+HClM=Li,Na,K,Rb,Cs

For KOTeF_5_, RbOTeF_5_, and CsOTeF_5_ this reaction is quantitative due to the good solubility of the respective chloride salt in HOTeF_5_. However, in the case of LiOTeF_5_ and NaOTeF_5_ a full conversion can only be achieved by grinding the reaction mixture after each addition of HOTeF_5_ or by performing the synthesis in acetonitrile.^11^ In 1974 Seppelt et al. proposed another route for the synthesis of LiOTeF_5_, as shown in eq 4.^94^ The lithium salt can be obtained by reacting LiOMe with an equimolar amount of HOTeF_5_. Nevertheless, the yield is only 38%, as the formed methanol solvates the product and can only be removed in high vacuum at higher temperatures, leading to the decomposition of the product. The sodium salt, on the other hand, can be obtained in almost quantitative yield by the reaction of Me_3_SiOTeF_5_ with NaOSiMe_3_ (see eq 5). All alkali metal salts are white crystalline solids that do not melt and are thermally stable up to at least 250 °C.^11,137^ They are nonhygroscopic but slowly hydrolyze under moist air or in aqueous solution. All compounds have been characterized by NMR, IR, and Raman spectroscopy, as well as by powder-XRD.^11,138^ In fact, it has been shown that all MOTeF_5_ compounds exhibit the structure of KOsF_6_, and due to identical unit cell volumes in their Cs^+^ salts, it could be concluded that [OTeF_5_]^−^ has a volume identical to that of the isoelectronic [SbF_6_]^−^.^11^


4 LiOMe→−MeOHHOTeF 5LiOTeF 5

5 NaOSiMe3→−(Me3Si)2OMe3SiOTeF 5NaOTeF 5

The respective alkali metal pentafluoro­orthoselenates MOSeF_5_ (M = Li, Na, K, Rb, Cs) are also known. Except for the sodium compound, all can be prepared by the conversion of the alkali metal fluoride with HOSeF_5_. Pure NaOSeF_5_ can be obtained by reacting Me_3_SiOSeF_5_ with NaOSiMe_3_.^88,94^ Group 1 teflates and selenates are gathered in Table 3.

**Table 3:** Overview of Group 1 (Alkali Metal) Teflate and Selenate Compounds

Element	Compound	Refs
Li	LiOTeF_5_	^11,94^
	LiOSeF_5_	^88^

Na	NaOTeF_5_	^11,94^
	NaOSeF_5_	^88,94^

K	KOTeF_5_	^11,137^
	KOSeF_5_	^88,94^

Rb	RbOTeF_5_	^11,138^
	RbOSeF_5_	^88^

Cs	CsOTeF_5_	^11,137,138^
	CsOSeF_5_	^88^

## 4 Group 2 Teflates

So far, no earth alkali metal teflate compounds have been described in the literature.

## 5 Group 13 Teflates

### 5.1 Boron Teflates

Boron tris­(pentafluoro­orthotellurate), B­(OTeF_5_)_3_, was first prepared by Sladky et al. through the neat reaction of BCl_3_ with 3 equiv of HOTeF_5_ at −80 °C.^9^ The compound forms hexagonal colorless crystals and can be sublimed at room temperature *in vacuo*.^139^ B­(OTeF_5_)_3_ has a remarkably high thermal stability up to 140 °C, which is unexpected, as comparable compounds containing fluorine atoms in the β-position to the boron center, such as B­(OCF_3_)_3_ or B­(SCF_3_)_3_, decompose above −20 °C by β-fluoride elimination.^54,63,140^ As mentioned earlier, B­(OTeF_5_)_3_ possesses an especially high FIA value of 506 kJ mol^–1^ and can therefore be considered as a Lewis superacid.^52,54^ In fact, its Lewis acidity was determined calorimetrically to be comparable to that of BCl_3_, and it forms stable adducts with pyridine, tetrahydropyran and acetonitrile.^9^ Anyhow, this compound is mainly used as OTeF_5_ transfer reagent in metathetical reactions with fluorides, in which the driving force is the formation of the volatile BF_3_ compound. B­(OTeF_5_)_3_ enabled the formation of a variety of different teflate compounds containing several different central atoms such as As,^18^ Au,^40^ Bi,^44^ Cl,^27^ F,^141^ Nb,^142^ Sb,^15^ Se,^87^ Ta,^142^ Te^22,28,57^ or Xe^1^. For more reaction details on each formed compound class see the corresponding section.

The corresponding anion [B­(OTeF_5_)_4_]^−^ was in the past considered as a candidate for the least coordinating anion.^143^ Therefore, the possibility to generate weakly coordinated metal and metalloid cations with the aforementioned borate as counterion has been thoroughly studied. The Lewis acid–base reaction of B­(OTeF_5_)_3_ with MOTeF_5_ (M = Ag, Tl) in the weakly coordinating solvents mesitylene (Mes), dichloromethane (DCM), 1,2-dichloroethane (DCE), and 1,1,2-trichloro-1,2,2-trifluoroethane (F-113) generates solutions of [M­(solv)_*n*_]​[B­(OTeF_5_)_4_] (*n* = 0–2) (see eq 6).^144^ The nonsolvated compound Ag­[B­(OTeF_5_)_4_] is isolated from a F-113 solution, whereas the nonsolvated salt Tl­[B­(OTeF_5_)_4_] is isolated from DCM or F-113.^144^ Both salts, Ag­[B­(OTeF_5_)_4_] and Tl­[B­(OTeF_5_)_4_] are thermally unstable, slowly decomposing to the corresponding metal teflate salt and the volatile B­(OTeF_5_)_3_. The molecular structure of Ag­[B­(OTeF_5_)_4_] reveals weak interactions between the Ag^+^ cations and the borate anion through three Ag–O contacts.^144^ In the case of Tl^+^ as counterion, molecular structures in the solid state are described with either 1,2-dichloroethane or mesitylene. In the first example, the solvent acts as a chelating ligand and, consequently, the salt [Tl­(1,2-C_2_H_2_Cl_2_)]​[B­(OTeF_5_)_4_] could be isolated by Strauss et al.^145^ The compound [Tl­(η^6^-Mes)_2_]​[B­(OTeF_5_)_4_] comprises a chain of [Tl­(Mes)_2_]^+^ cations and [B­(OTeF_5_)_4_]^−^ anions connected by weak Tl–F interactions (see Figure 6).^143^


BCl3→−3HCl3HOTeF 5B(OTeF 5)3→MOTeF 5solv[M(solv)n][B(OTeF 5)4]M=Ag,Tl;n=0−2solv=DCE,DCM,F‐113,Mes 6

**Figure 6:**
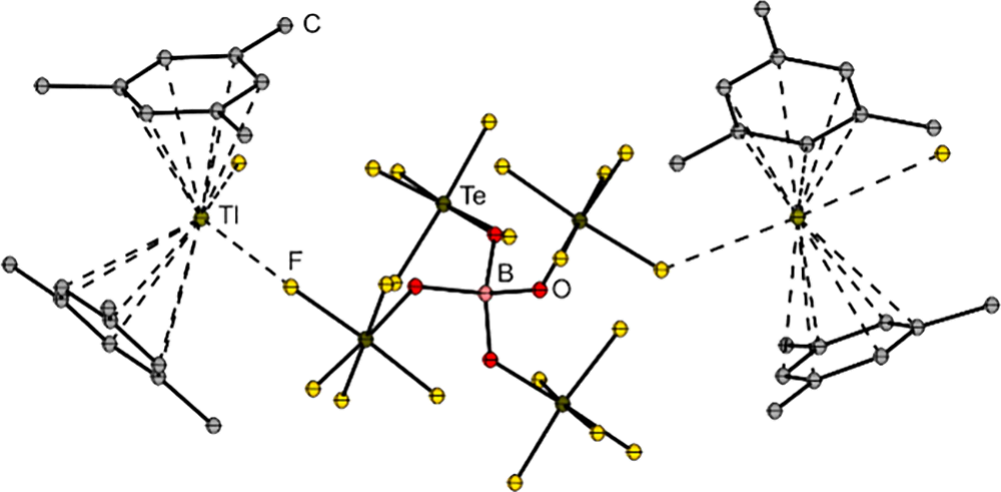
Molecular structure of [Tl­(Mes)_2_]​[B­(OTeF_5_)_4_] in the solid state.^143^

However, attempts to generate the cations [Fe­(OEP)]^+^, [Fe­(TPP)]^+^ or [SiPh_3_]^+^ by the reaction of Ag­[B­(OTeF_5_)_4_] or Tl­[B­(OTeF_5_)_4_] with Fe­(OEP)Cl (OEP = 2,3,7,8,12,13,17,18-octaethylporphyrinate dianion), Fe­(TPP)Cl (TPP = 5,10,15,20-tetraphenylporphyrinate dianion) or Ph_3_SiCl in dichloromethane or mesitylene failed. Instead, an OTeF_5_ abstraction occurs and B­(OTeF_5_)_3_ together with [Fe­(OEP)​(OTeF_5_)], [Fe­(TPP)​(OTeF_5_)] or Ph_3_SiOTeF_5_ are obtained, respectively. The reaction of the tritylium salt [CPh_3_]​[B­(OTeF_5_)_4_] with Ph_3_SiH in dichloromethane was also not successful and produced Ph_3_SiOTeF_5_.^144^

Nevertheless, due to the weakly coordinating character of the [B­(OTeF_5_)_4_]^−^ anion the first isolation of the silver carbonyl complexes [AgCO]^+^ and [Ag­(CO)_2_]^+^ was possible.^146–148^ The nature of these compounds have been comprehensively studied by X-ray crystallography as well as solid-state IR- and multinuclear NMR spectroscopy. Other reactive cations, such as [XeC_6_F_5_]^+^, could also be stabilized through the use of this weakly coordinating anion. This cation has been prepared in a metathesis reaction of [XeC_6_F_5_]​[BF_4_] with the corresponding M­[B­(OTeF_5_)_4_] salt (M = K or Cs),^149^ which itself has been synthesized by reacting MOTeF_5_ with B­(OTeF_5_)_3_.^63^

Quaternary ammonium salts also exist with this counterion. The [NMe_4_]​[B­(OTeF_5_)_4_] salt has been synthesized by the reaction of [NMe_4_]​[OTeF_5_] with B­(OTeF_5_)_3_, and was compared to the isoelectronic C­(OTeF_5_)_4_ species.^150^

### 5.2 Aluminum Teflates

The Lewis superacid Al­(OTeF_5_)_3_ is synthesized by reacting either triethyl aluminum or trimethyl aluminum with teflic acid in *n*-pentane, with the use of AlMe_3_ leading to a purer product. If the reaction temperature is kept at −40 °C, only two of the three methyl groups of AlMe_3_ are substituted by the OTeF_5_ ligand resulting in AlMe­(OTeF_5_)_2_, which is found to be an oxygen-bridged dimer [AlMe­(OTeF_5_)_2_]_2_ in the solid state (see eq 7). When the reaction mixture is allowed to warm up to room temperature, the homoleptic compound Al­(OTeF_5_)_3_ is formed.^56^ The vibrational analysis in combination with quantum-chemical calculations of this Lewis acid suggests that it exists as oxygen-bridged dimer [Al­(OTeF_5_)_3_]_2_.^55^ The dimeric structure can readily be broken up by addition of weakly coordinating solvents such as fluorobenzene or sulfuryl chloride fluoride, forming [Al­(OTeF_5_)_3_(C_6_H_5_F)_2_] and [Al­(OTeF_5_)_3_(SO_2_ClF)_2_], respectively.^56^ Additionally, several adducts of the Lewis acid containing up to three ligands per aluminum center have been described. These are either synthesized by starting from [Al­(OTeF_5_)_3_(SO_2_ClF)_2_] and exchanging the SO_2_ClF with diethyl ether (Et_2_O) or triethylphosphine oxide, or by using the desired ligand as solvent as seen with MeCN and PhCN. Interestingly, the 6-fold coordinated nitrile adducts [Al­(OTeF_5_)_3_(RCN)_3_] (R = Me, Ph) autoionize (see eq 8) in solution and coexist with the ion-pair [Al­(OTeF_5_)_2_(RCN)_4_]​[Al­(OTeF_5_)_4_(RCN)_2_]. However, in the solid state, [Al­(OTeF_5_)_3_(PhCN)_3_] crystallizes as the neutral compound. The autoionization is induced by addition of 2,2′-bipyridine to a [Al­(OTeF_5_)_3_(PhCN)_3_] solution, leading to the salt [Al­(OTeF_5_)_2_(bpy)_2_]​[Al­(OTeF_5_)_4_(bpy)] (see eq 8).


7 2AlMe3+6HOTeF 5→−4CH4[AlMe(OTeF 5)2]2+2HOTeF 5→−2CH4[Al(OTeF 5)3]2

8 AlEt3+3HOTeF 5→−2C2H6RCN[AlMe(OTeF 5)3(RCN)3]⇌12[Al(OTeF 5)2(RCN)4][Al(OTeF 5)4(RCN)2]R=Me,Ph

The toluene adduct of the Lewis acid is accessible by a synthetic detour. First, the protonated methyl benzenium cation and the weakly coordinating [Al­(OTeF_5_)_4_]^−^ anion are formed by treating triethyl aluminum with 4 equiv of teflic acid in toluene, (for further information on those Brønsted acids see below). In a second step, addition of Et_3_SiH leads to the formation of the toluene adduct [Al­(OTeF_5_)_3_(η^1^-C_7_H_8_)], presumably via a cationic silylium species [SiEt_3_]^+^ (see eq 9).


9 AlEt3+4HOTeF 5→−3C2H6toluene[H‐C7H8][Al(OTeF 5)4]→Et3SiH[SiEt3][Al(OTeF 5)4](not isolable)→−Et3SiOTeF 5[Al(OTeF 5)3(η1‐C7H8)]

The Lewis acidity at the Al center of the experimentally observed compounds decreases in the order [Al­(OTeF_5_)_3_]_2_ > [Al­(OTeF_5_)_3_(η^1^-C_7_H_8_)] > [Al­(OTeF_5_)_3_(SO_2_ClF)_2_] > [Al­(OTeF_5_)_3_(C_6_H_5_F)_2_] > [Al­(OTeF_5_)_3_(MeCN)_3_] > [Al­(OTeF_5_)_3_(PhCN)_3_] > [Al­(OTeF_5_)_3_(Et_2_O)_2_]. Among these the FIA values of [Al­(OTeF_5_)_3_]_2_, [Al­(OTeF_5_)_3_(C_7_H_8_)], [Al­(OTeF_5_)_3_(SO_2_ClF)_2_], and [Al­(OTeF_5_)_3_(C_6_H_5_F)_2_] surpass the FIA value of SbF_5_, thus classifying them as Lewis superacids.^53^

Interestingly, [Al­(OTeF_5_)_3_]_2_ can also be utilized to access an anion-doped aluminum chlorofluoride of the type AlCl_0.1_F_2.8_(OTeF_5_)_0.1_. This was achieved by treating a mixture of AlCl_3_ and [Al­(OTeF_5_)_3_]_2_ with CCl_3_F. Further analysis revealed the highly Lewis acidic nature of this material and its potential for catalyzing dehydrofluorination reactions.^151^

The addition of 1 equiv of HOTeF_5_ to the Lewis acid results in the formation of a conjugated Brønsted/Lewis acid HOTeF_5_/Al­(OTeF_5_)_3_ in analogy to the well-known binary systems HF/SbF_5_ and HSO_3_F/SbF_5_.^152^ Based on the basicity of the used solvent, the Brønsted acidity can be tuned, whereas the use of different arenes is reported.^55^ Experiments showed *ortho*-difluorobenzene to be a promising candidate for this purpose by combining a low proton affinity (leading consequently to a high Brønsted acidity of its protonated derivative) and a good solubility even at low temperatures. The Brønsted superacid [H-C_6_H_4_F_2_]​[Al­(OTeF_5_)_4_]_(solv)_ is synthesized according to Figure 7 and can be further reacted with either chloride salts to form corresponding salts of the WCA [Al­(OTeF_5_)_4_]^−^ ^55,153,154^ or with more basic substrates than *ortho*-difluorobenzene to protonate these. It was shown that the protonation of arenes such as benzene and mesitylene,^55^ or several pyridines^55,74^ can be achieved. Remarkably, even white phosphorus^72^ is protonated, forming a [P_4_H]^+^ cation with the proton on a P–P edge of the P_4_ tetrahedron. Additionally, when C_5_F_5_N and C_5_Cl_5_N are used in excess, the corresponding pyridine adducts of the pyridinium cations [(C_5_F_5_N)_2_H]^+^ and [(C_5_F_5_N)_2_H]^+^ are available.

**Figure 7:**
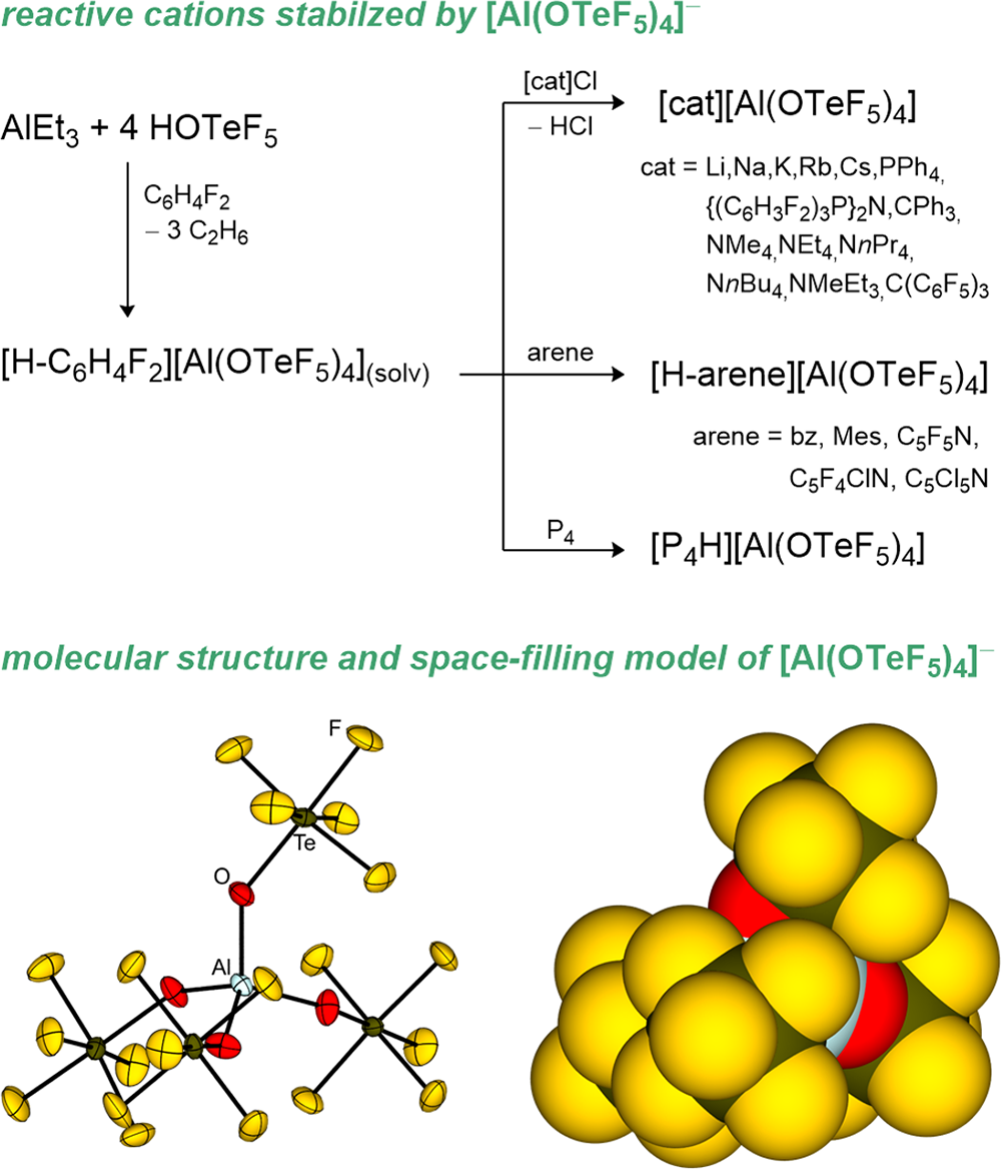
Synthetic route to the acid [H-C_6_H_4_F_2_]​[Al­(OTeF_5_)_4_]_(solv)_ and follow-up chemistry (upper chart). Molecular structure (lower chart) in the solid state of [PPh_4_]​[Al­(OTeF_5_)_4_] (left) and the space-filling representation (right).^55^ Cations are omitted for clarity. Thermal ellipsoids are set at 50% probability.

The *in situ* generation of the Brønsted superacid in chloromethane gives rise to the strong methylation agent [Cl­(Me)_2_]​[Al­(OTeF_5_)_4_]. Hereby, chloromethane is partially protonated and reacts with another equivalent of MeCl to the dimethyl chloronium cation. Several weak bases have been methylated, including MeBr, MeI, PF_3_, P­(CF_3_)_3_, C_5_H_5_P, 2-(Me_3_Si)­C_5_H_4_P, and 2,3,5,6-(Me_3_Si)_4_C_5_HP. The methylated products are depicted in Figure 8.^73,155^

**Figure 8:**
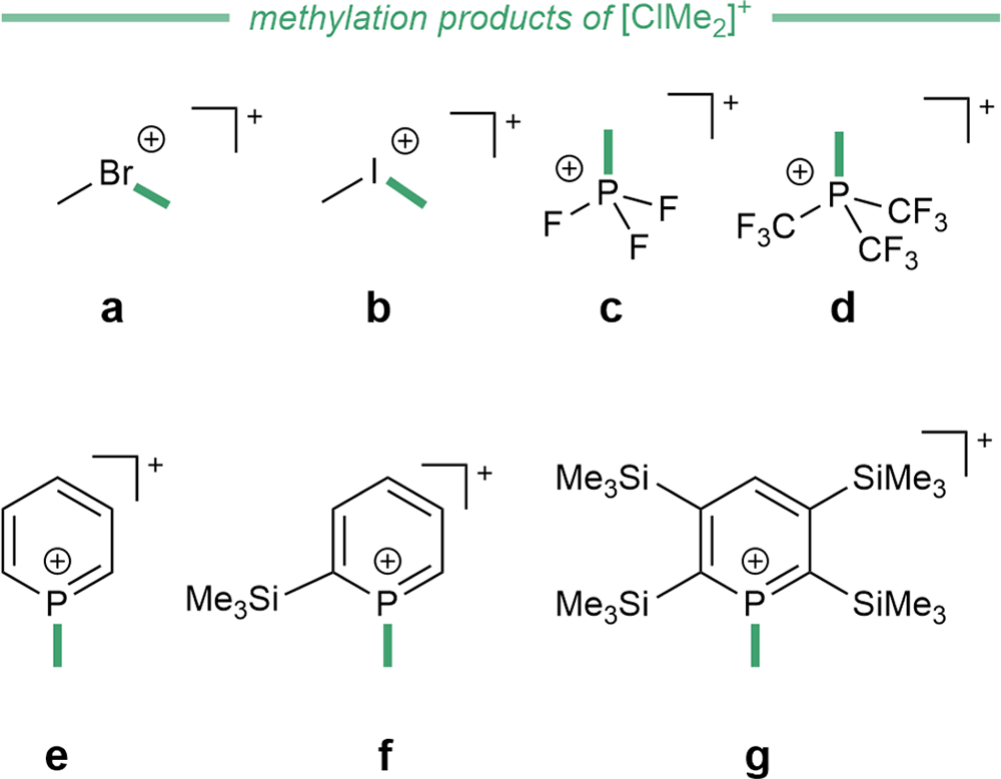
Methylation products of the reaction of [Cl­(CH_3_)_2_]​[Al­(OTeF_5_)_4_] with MeBr (a), MeI (b), PF_3_ (c), P­(CF_3_)_3_ (d), C_5_H_5_P (e), 2-(Me_3_Si)­C_5_H_4_P (f), and 2,3,5,6-(Me_3_Si)_4_C_5_HP (g). The [Al­(OTeF_5_)_4_]^−^ is omitted for clarity.

In order to form the synthetically useful silver salt Ag­[Al­(OTeF_5_)_4_], which has the potential to be used as a halide abstraction or oxidizing agent, the acid [H-C_6_H_4_F_2_]​[Al­(OTeF_5_)_4_]_(solv)_ is reacted with AgF.^51^ Other silver salts, containing weakly coordinating anions, have successfully been used in the synthesis of reactive cations.^156–162^ Furthermore, the strongly oxidizing [NO]^+^, [NOP_4_]^+^, [Xe­(OTeF_5_)​(C_5_H_3_F_2_N)]^+^ and [Xe­(OTeF_5_)​(C_5_F_5_N)]^+^ salts of the WCA have been prepared.^51,70^ In the case of the nitrosonium salt, the metathesis reaction of Li­[Al­(OTeF_5_)_4_] and [NO]​[SbF_6_] is performed. Upon addition of P_4_, [NOP_4_]​[Al­(OTeF_5_)_4_] is formed quantitatively. The xenonium ions are synthesized starting from the Lewis superacid [Al­(OTeF_5_)_3_]_2_ and Xe­(OTeF_5_)_2_, followed by the addition of the respective pyridine (see eq 10). This reaction demonstrates on the one hand the high Lewis acidity of [Al­(OTeF_5_)_3_]_2_ and on the other hand the robustness of the WCA [Al­(OTeF_5_)_4_]^−^ toward strong oxidizers. Upon addition of an excess of CH_2_ClCF_3_ to a mixture of [Al­(OTeF_5_)_3_]_2_ and Xe­(OTeF_5_)_2_ in SO_2_ClF, the chlorine atom of the chloro­fluoro­alkane is oxidized, leading to the fluorinated dialkyl chloronium salt [Cl­(CH_2_CF_3_)_2_]​[Al­(OTeF_5_)_4_].^75^


10 12[Al(OTeF 5)3]2+Xe(OTeF 5)2→1.SO2ClF2.pyF[Xe(OTeF 5)(pyF)][Al(OTeF 5)4]pyF=C 5H3F 2N,C 5F 5N

Interestingly, the tetrahedral coordination sphere of the anion [Al­(OTeF_5_)_4_]^−^ can be further extended. For instance, treatment of [Al­(OTeF_5_)_3_(SO_2_ClF)_2_] with an excess of [NEt_4_]​[OTeF_5_] gives rise to the trigonal bipyramidal dianion [NEt_4_]_2_[Al­(OTeF_5_)_5_].^56^

### 5.3 Gallium Teflates

The Lewis superacid Ga­(OTeF_5_)_3_ is obtained by the neat reaction of GaCl_3_ and ClOTeF_5_ with the release of gaseous Cl_2_, as shown in eq 11.^26^ The thermally stable product is a rare example of an isolable gallium-based hard and soft Lewis superacid as indicated by its high fluoride and hydride ion affinity. The shift difference in the ^31^P NMR spectra upon coordination of OPPh_3_ to the Lewis acid (modified Gutmann–Beckett method) shows that its Lewis acidity is comparable to that of the aluminum-based Lewis superacid Al­(OTeF_5_)_3_. In analogy to this Lewis acid, vibrational analysis in combination with quantum-chemical calculations suggests that Ga­(OTeF_5_)_3_ forms the oxygen-bridged dimer [Ga­(OTeF_5_)_3_]_2_ in the solid state. The high Lewis acidity of this compound allows the formation of adducts with weak nucleophiles like penta­fluoro­pyridine C_5_F_5_N (see Figure 9, left).


11 GaCl3→−3Cl23ClOTeF 5Ga(OTeF 5)3

**Figure 9:**
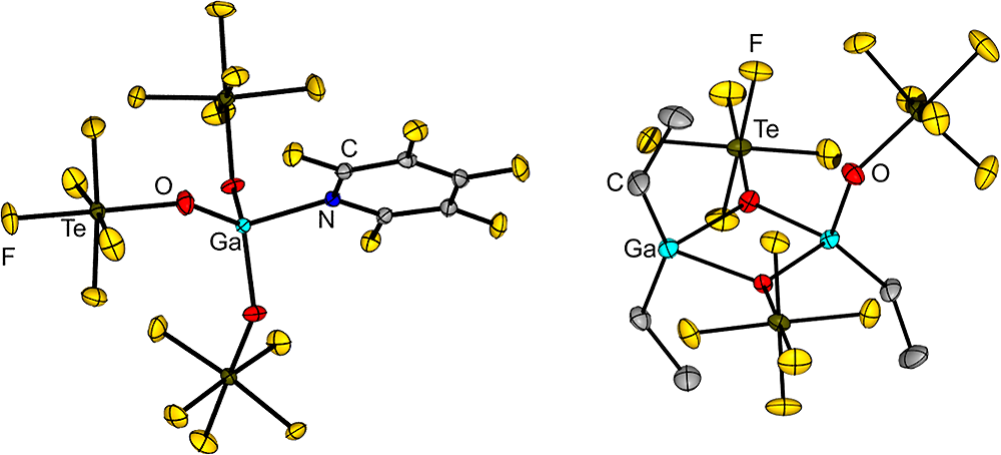
Structures of [Ga­(OTeF_5_)_3_(C_5_F_5_N)] (left) and Ga_2_Et_3_(OTeF_5_)_3_ (right) in the solid state.^26,66^ Thermal ellipsoids are set at 50% probability.

The addition of 1 equiv of HOTeF_5_ leads to the formation of a conjugated Brønsted/Lewis acid HOTeF_5_/Ga­(OTeF_5_)_3_ as already reported for the aluminum analogue. With diethyl ether this acidic system forms the oxonium salt [H­(Et_2_O)_2_]​[Ga­(Et_2_O)_2_(OTeF_5_)_4_] (see eq 12). The high Lewis acidity of Ga­(OTeF_5_)_3_ can further be utilized to access a strong oxidizing system with Xe­(OTeF_5_)_2_ in SO_2_ClF, which is able to oxidize chlorine atoms of chloromethane MeCl to give the dimethyl chloronium salt [ClMe_2_]​[Ga­(OTeF_5_)_4_] (see eq 12a).^26^


12 Ga(OTeF 5)3→HOTeF 5,4Et2O[H(Et2O)2][Ga(Et2O)2(OTeF 5)4]

13 Ga(OTeF 5)3→Xe(OTeF 5)2,MeCl[ClMe2][Ga(OTeF 5)4]

The corresponding weakly coordinating anion [Ga­(OTeF_5_)_4_]^−^ is prepared as the Ag^+^ salt by the reaction of GaCl_3_ with AgOTeF_5_, as shown in eq 13. Subsequent reaction of the silver salt with [PPh_4_]­Cl, and CClPh_3_, led to the formation of the [PPh_4_]^+^ and the [CPh_3_]^+^ salt of [Ga­(OTeF_5_)_4_]^−^.^66^


14 GaCl3→−3AgCl4AgOTeF 5Ag[Ga(OTeF 5)4]→−AgCl[cat]Cl[cat][Ga(OTeF 5)4]cat=PPh4,CPh3

In addition, the partially teflate-substituted anion [GaEt­(OTeF_5_)_3_]^−^ was isolated as several different salts, resulting from the incomplete reaction of GaEt_3_ and HOTeF_5_. Within the latter process, it was possible to isolate and characterize the reactive species Ga_2_Et_3_(OTeF_5_)_3_, which can be described as a contact ion pair between a cationic [GaEt_2_]^+^ and an anionic [GaEt­(OTeF_5_)_3_]^−^ fragment (see Figure 9, right). It does not react further with HOTeF_5_ in contrast to the Al analogue Al_2_Et_3_(OR^F^)_3_ (R^F^ = C­(CF_3_)_3_), in which the ethyl groups can be further substituted by OC­(CF_3_)_3_ substituents.^163^

### 5.4 Thallium Teflates

TlOTeF_5_ has been synthesized by the reaction of TlF with HOTeF_5_.^143,164^ The salt is soluble in toluene and mesitylene and can be crystallized as [TlOTeF_5_(Mes)_2_]_2_·Mes. The molecular structure reveals a η^6^-coordination of mesitylene to the thallium atoms and two bridging OTeF_5_ groups that is comparable with reported structures of AgOTeF_5_.^165^ The neutral Tl­(III) teflate can be synthesized from the reaction of TlCl_3_ and 3 equiv of ClOTeF_5_, or from TlCl_3_ and Xe­(OTeF_5_)_2_ upon formation of Cl_2_ and Xe, respectively. The molecular structure reveals that this compound forms a dimer [Tl­(OTeF_5_)_3_]_2_ in the solid state.^27^ Group 13 teflates are gathered in Table 4.

**Table 4:** Overview of Group 13 Teflate Compounds

Element	Ox	Compound	Refs		Compound	Refs
B	III	B(OTeF_5_)_3_	^9,63,139^		[Tl(DCE)]​[B(OTeF_5_)_4_]	^145^
		B[(OTeF_5_)_3_(py)]	^9^		[Tl(η^6^-Mes)_2_]​[B(OTeF_5_)_4_]	^143^
		B[(OTeF_5_)_3_(THP)]	^9^		[N(*n*Bu)_4_]​[B(OTeF_5_)_4_]	^143^
		B[(OTeF_5_)_3_(MeCN)]	^9,63^		[AgCO]​[B(OTeF_5_)_4_]	^146,147^
		B[(OTeF_5_)_3_(OPEt_3_)]	^136^		[Ag(CO)_2_]​[B(OTeF_5_)_4_]	^147,148^
		B[(OTeF_5_)_3_(OPPh_3_)]	^26^		[NMe_4_]​[B(OTeF_5_)_4_]	^150^
		Cs[B(OTeF_5_)_4_]	^63^		[XeC_6_F_5_]​[B(OTeF_5_)_4_]	^149^
		Ag[B(OTeF_5_)_4_]	^144^		[SeF_3_]​[B(OTeF_5_)_4_]	^150^
		Tl[B(OTeF_5_)_4_]	^144^			

Al	III	[Al(OTeF_5_)_3_]_2_	^56^		[{(C_6_H_3_F_2_)_3_P}_2_N]​[Al(OTeF_5_)_4_]	^154^
		[AlMe(OTeF_5_)_2_]_2_	^56^		[CPh_3_]​[Al(OTeF_5_)_4_]	^55^
		[Al(OTeF_5_)_3_(η^1^-C_7_H_8_)]	^56^		[C(C_6_F_5_)_3_]​[Al(OTeF_5_)_4_]	^71^
		[Al(OTeF_5_)_3_(OPEt_3_)]	^56^		[C_6_H_7_]​[Al(OTeF_5_)_4_]	^55^
		[Al(OTeF_5_)_3_(OPPh_3_)]	^26^		[C_9_H_13_]​[Al(OTeF_5_)_4_]	^55^
		[Al(OTeF_5_)_3_(C_6_H_5_F)_2_]	^56^		[P_4_H]​[Al(OTeF_5_)_4_]	^72^
		[Al(OTeF_5_)_3_(Et_2_O)_2_]	^56^		[C_5_F_5_NH]​[Al(OTeF_5_)_4_]	^74^
		[Al(OTeF_5_)_3_(SO_2_ClF)_2_]	^56^		[C_5_F_4_ClNH]​[Al(OTeF_5_)_4_]	^74^
		[Al(OTeF_5_)_3_(MeCN)_3_]	^56^		[(C_5_F_5_N)_2_H]​[Al(OTeF_5_)_4_]	^74^
		[Al(OTeF_5_)_3_(PhCN)_3_]	^56^		[(C_5_Cl_5_N)_2_H]​[Al(OTeF_5_)_4_]	^74^
		[H-C_6_H_4_F_2_]​[Al(OTeF_5_)_4_]_(solv)_	^55^		[Me_2_X]​[Al(OTeF_5_)_4_] (X = Cl, Br, I)	^73^
		[Ag(C_6_H_4_F_2_)_2_]​[Al(OTeF_5_)_4_]	^51^		[X(CH_2_CF_3_)]​[Al(OTeF_5_)_4_] (X = Cl, I)	^75^
		M[Al(OTeF_5_)_4_] (M = Li, Na, K, Rb, Cs)	^51,55^		[MePF_3_]​[Al(OTeF_5_)_4_]	^73^
		[NO]​[Al(OTeF_5_)_4_]	^51^		[MeP(CF_3_)_3_]​[Al(OTeF_5_)_4_]	^73^
		[P_4_NO]​[Al(OTeF_5_)_4_]	^51^		[C_5_H_5_PMe]​[Al(OTeF_5_)_4_]	^155^
		[Xe(OTeF_5_)​(C_5_F_5_N)]​[Al(OTeF_5_)_4_]	^70^		[2-(Me_3_Si)C_5_H_4_PMe]​[Al(OTeF_5_)_4_]	^155^
		[Xe(OTeF_5_)​(C_5_H_3_F_2_N)]​[Al(OTeF_5_)_4_]	^70^		[2,3,5,6-(Me_3_Si)_4_C_5_HPMe]​[Al(OTeF_5_)_4_]	^155^
		[NR_4_]​[Al(OTeF_5_)_4_] (R = Me, Et, *n*Pr, *n*Bu)	^153^		[NEt_4_]_2_[Al(OTeF_5_)_5_]	^56^
		[NMeEt_3_]​[Al(OTeF_5_)_4_]	^153^		[Al(OTeF_5_)_2_(bpy)_2_]​[Al(OTeF_5_)_4_(bpy)]	^56^
		[PPh_4_]​[Al(OTeF_5_)_4_]	^55^			

Ga	III	Ga(OTeF_5_)_3_	^26^		[H(Et_2_O)_2_]​[Ga(Et_2_O)_2_(OTeF_5_)_4_]	^26^
		Ga[(OTeF_5_)_3_(C_5_F_5_N)]	^26^		[cat]​[Ga(Et)​(OTeF_5_)_3_] (cat = PPh_4_, CPh_3_)	^66^
		Ga[(OTeF_5_)_3_(OPPh_3_)]	^26^		Ga_2_(Et)_3_(OTeF_5_)_3_	^66^
		[cat]​[Ga(OTeF_5_)_4_] (cat = Ag, PPh_4_, CPh_3_, ClMe_2_)	^26,66^			

Tl	I	[Tl(η^6^-Mes)_2_OTeF_5_]_2_	^143,164^			
	III	Tl(OTeF_5_)_3_	^27^			

## 6 Group 14 Teflates

### 6.1 Carbon Teflates

In contrast to the well-explored inorganic compounds bearing the teflate ligand, only a few organic derivatives have been prepared so far. Two classes of compounds were reported, hydrocarbons (C–H) and fluorocarbons (C–F). Furthermore, the two homoleptic carbon teflate compounds [C­(OTeF_5_)_3_]^+^ and C­(OTeF_5_)_4_ will be highlighted.

#### 6.1.1 Hydrocarbons (C_*n*_H_2*n*+2_)

Most synthetic efforts proceed via a unique pathway developed by Fraser et al., which involves the direct reaction of tellurium hexafluoride (TeF_6_) with alcohols (R-OH, R = linear and branched alkyl) (see eq 14 and Figure 10).^166–168^ These derivatives as well as the bis-alkoxy derivatives TeF_4_(OR)_2_ found their place as alkylation reagents in 1979.^169^


15 TeF 6+R‐OH→R‐OTeF 5+HF

**Figure 10:**
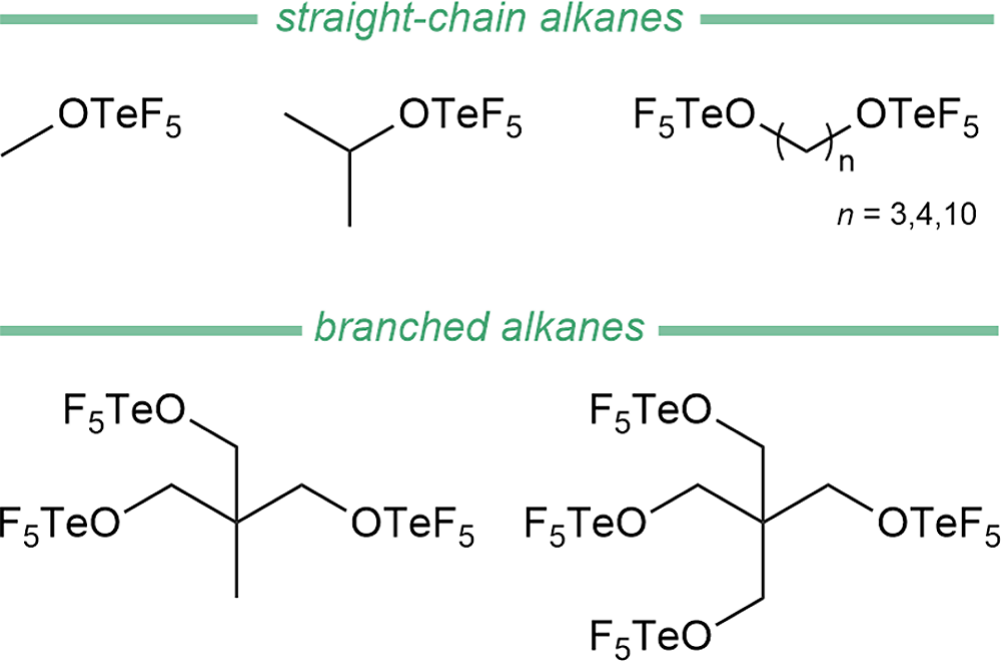
Selected teflate-functionalized hydrocarbons.

This synthetic route enables the formation of alkoxotellurium derivatives in moderate to good yields (up to 75%), with a scope including linear and branched alcohols (C_1_–C_4_). The formed HF is deactivated with NaF, Na_2_CO_3_ or pyridine. These compounds have been observed analytically in similar vibrational or chemical shift ranges in spectroscopic techniques (IR and NMR), indicating minimal influence of the alkyl substituents on the tellurium center. The typical AB_4_ spin system of the teflate group is observed in the ^19^F NMR spectra of these compounds, consistent with other covalently bonded OTeF_5_ groups. Further NMR studies and structural analysis of the MeOTeF_5_ compound were conducted by Seppelt et al.^49^ A recent report on a modern teflate-based reagent (dimethylchloronium salt [ClMe_2_]​[Al­(OTeF_5_)_4_])^73^ also mentions the generation of carbon-based species CH_2_ClOTeF_5_ and CH_2_(OTeF_5_)_2_ by its decomposition. The compounds were not isolated.

Finally, this strategy was extended by using diols, enabling the synthesis of bridged teflates F_5_TeO-(CH_2_)_*n*_-OTeF_5_ (*n* = 3, 4, 10).^170^ Furthermore, only two branched polyols (trimethylolethane and pentaerythritol) have been successfully converted into the desired organic teflate derivatives (see Figure 10). More recently, both CH_2_ClOTeF_5_ and CH_2_(OTeF_5_)_2_ were observed from the decomposition of [Al­(OTeF_5_)_4_]^−^.^171^

#### 6.1.2 Fluorocarbons

In contrast to the hydrocarbon compounds discussed above, the synthesis of fluorocarbon derivatives utilizes various established OTeF_5_ transfer reagents like Xe­(OTeF_5_)_2_ and XOTeF_5_ (X = Cl, F). First, hypohalites were engaged with fluorinated olefins, enabling the synthesis of linear derivatives (C_2_, C_3_ and C_5_) in good yields (60–86%).^172^ The use of fluoroolefins with Xe­(OTeF_5_)_2_ allows the synthesis of fluorinated bis-OTeF_5_ species via a formal 1,2-addition reaction over the double bond and liberation of Xe (see Figure 11).^173^ Christe and Schack successfully prepared cyclic derivatives from the hypofluorite FOTeF_5_ and synthesized an example of an unsaturated bis-teflate compound, as shown in Table 5, which provides an overview of hydro- and fluorocarbon-based teflate compounds.^174^ Finally, fluorinated alkyl chains bearing the OTeF_5_ group were prepared through the direct reaction of the hypochlorite ClOTeF_5_ with fluorocarbon iodides, allowing excess to CF_3_OTeF_5_, C_2_F_5_OTeF_5_ and *n*-C_3_F_7_OTeF_5_.^175^ Several patents from Christe’s work were subsequently filed.^176–178^

**Figure 11:**
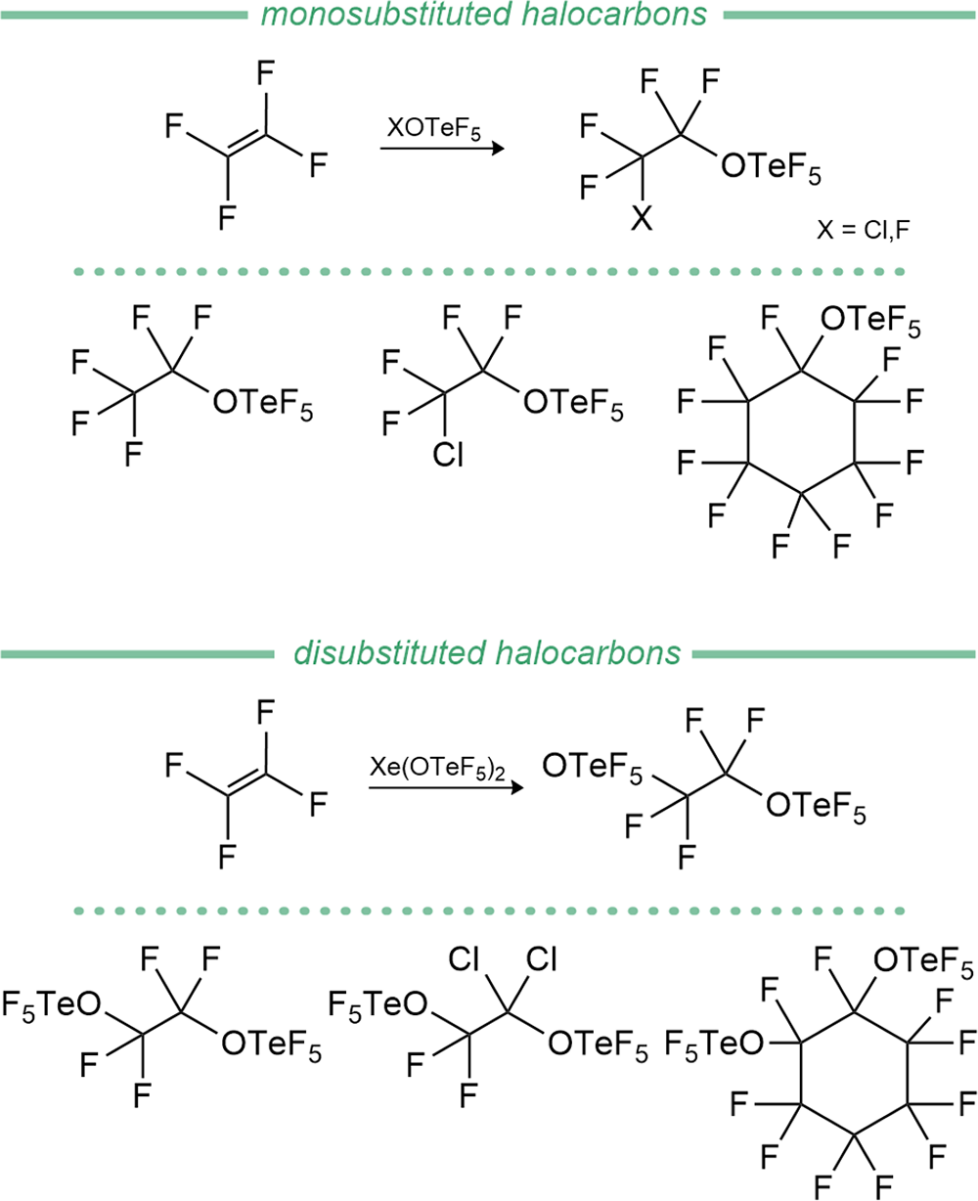
Exemplary synthesis and selected examples of halocarbon-based teflate compounds from hypohalites (upper chart) and from Xe­(OTeF_5_)_2_ (lower chart).

In 2004, Schrobilgen et al. presented the first carbon-based teflate compounds with nonorganic substituents, synthesizing [CBr_*n*_(OTeF_5_)_3‑*n*_]^+^ (*n* = 0–2)^67^ by oxidizing CBr_4_ with stoichiometric amounts of the strong oxidizer, [XeOTeF_5_]​[Sb­(OTeF_5_)_6_], followed by cascade reactions in which the formed [CBr_3_]^+^ reacts further with BrOTeF_5_ that formed during the process (see Scheme 4).

**Scheme 4:**
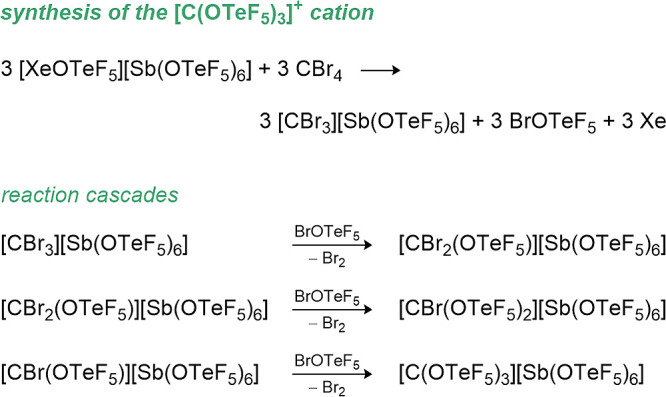
Oxidation of CBr_4_ to [CBr_*n*_(OTeF)_3‑*n*_]^+^ Cations (*n* = 0, 1, 3)

All compounds are stable at 0 °C for several hours. The OTeF_5_ derivatives were confirmed by ^19^F NMR and ^13^C NMR spectroscopy except for [CBr_2_(OTeF_5_)]^+^ since that ^13^C chemical shift could not be detected. The cation [C­(OTeF_5_)_3_]^+^ is isoelectronic and isostructural to B­(OTeF_5_)_3_ and was also characterized in the solid state (see Figure 12, left).^67^

**Figure 12:**
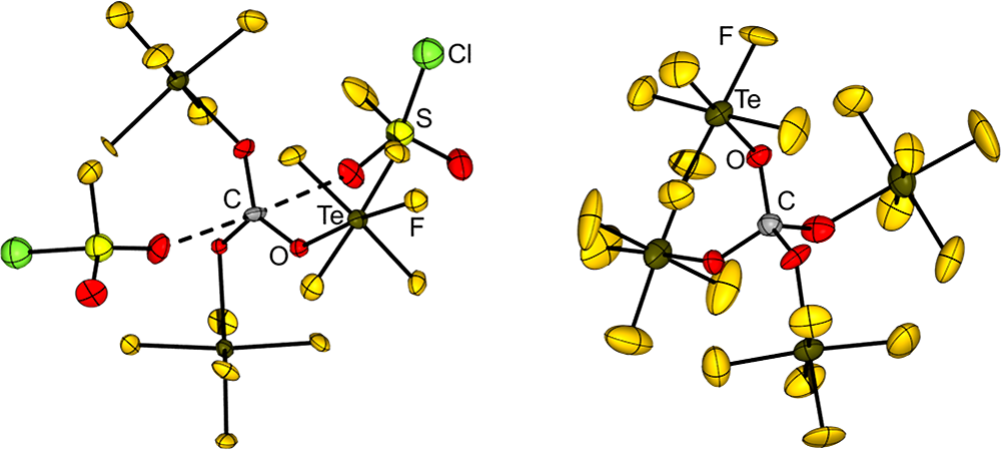
Structures of [C­(OTeF_5_)_3_]​[Sb­(OTeF_5_)_6_]·3SO_2_ClF (left) and C­(OTeF_5_)_4_ (right) in the solid state.^67,150^ Thermal ellipsoids are set at 50% and 30% probability, respectively. The counterion [Sb­(OTeF_5_)_6_]^−^ is omitted for clarity.

The reaction of CBr_4_ with stoichiometric amounts of BrOTeF_5_ yielded the homoleptic C­(OTeF_5_)_4_,^67^ which decomposes at 10 °C to O­(TeF_5_)_2_ and CO_2_ in MeCN.^150^ The formation of C­(OTeF_5_)_4_ and the ^13^C-enriched ^13^C­(OTeF_5_)_4_ was validated by ^13^C NMR, ^19^F NMR, and ^125^Te NMR spectroscopy^67^ as well as by single crystal X-ray diffraction of C­(OTeF_5_)_4_ (see Figure 12, right).^150^ Due to the more covalent C–O bond character in [C­(OTeF_5_)_3_]^+^ the C–O bond lengths in C­(OTeF_5_)_4_ are longer than in the corresponding cation (135(2)–142(2) pm vs 125.8(15)–131.3(16) pm).^150^

Carbon-based OSeF_5_ representatives are CH_3_OSeF_5_, CH_2_(OSeF_5_)_2_, CH­(OSeF_5_)_3_ and C­(OSeF_5_)_4_. Except for CH_2_(OSeF_5_)_2_, all four other compounds can be obtained by the OSeF_5_ transfer reagent Hg­(OSeF_5_)_2_. Surprisingly, this procedure was not successful for CH_2_Cl_2_ and yielded CH_2_(OSeF_5_)_2_ only in traces. However, if CH_2_Br_2_ and ClOSeF_5_ are reacted the desired product is formed.^86^

In a similar way the compounds F_5_SeO-COCl^86^ and F_5_SeO-COCF_3_ ^90^ are synthesized, starting from Hg­(OSeF_5_)_2_ and phosgene or trifluoroacetyl chloride.

Similarly to the pentafluoro­oxosulfate chemistry described in Section 2.5, Cady relied on the hypofluorite (FOSeF_5_) for the direct [2+2] reaction with a fluorinated alkene (i.e., perfluoro­cyclopentene).^98^ This allowed the synthesis of the desired fluorinated cyclic five-member ring C_5_F_9_OSeF_5_ in high yield. Furthermore, the oxidative ability of this reagent was highlighted by its reaction with carbon monoxide affording the corresponding acyl fluoride bearing the pentafluoro­orthoselenate moiety (F_5_SeO-COF).^98^

Lastly, the synthesis of the cyanate (F_5_SeOCN) was achieved by the direct reaction of Xe­(OSeF_5_)_2_ as transfer reagent with hydrogen cyanide.^89^

### 6.2 Silicon Teflates

While a variety of carbon-based teflate compounds is known, the teflate chemistry of the heavier group 14 homologues is largely unexplored. Generally, silicon based teflates are synthesized by reacting an organosilicon chloride with a teflate transfer reagent such as AgOTeF_5_ or HOTeF_5_ resulting in the targeted compound and the formation of volatile or poorly soluble molecules like HCl or AgCl as driving force (see eq 15).


16 SiCl4+4AgOTeF 5→Si(OTeF 5)4+4AgCl

The homoleptic silicon teflate Si­(OTeF_5_)_4_ was briefly mentioned by Sladky et al. in 1973 and its characterization is limited to the melting point of 37 °C.^19^ All other known silicon teflates contain only one teflate group.

The synthesis of Me_3_SiOTeF_5_ was first described in 1973 using Me_3_SiCl and HOTeF_5_ and the compound was characterized by the boiling point. Sladky et al. tested Me_3_SiOTeF_5_ as a teflate transfer reagent in metathesis reactions with PF_5_ and SF_4_.^19^ In those two reactions the tellurium atom was fluorinated, resulting in TeF_6_, Me_3_SiF, POF_3_, and SOF_2_. In the case of TeF_6_ no reaction took place. For the covalent halide AsF_5_ it was observed that F_2_AsOTeF_5_ is obtained, which further dismutates to AsF_3_ and As­(OTeF_5_)_3_.^19^ In 2018 the compound Me_3_SiOTeF_5_ was fully characterized by Riedel et al. and its application as teflate transfer reagent could be expanded to the heteroleptic organo gold­(III) complex [AuF_3_(SIMes)] (SIMes = 1,3-bis­(2,4,6-trimethylphenyl)-4,5-dihydroimidazol-2-yl­idene). Due to the introduced teflate group the Lewis acidity of the Au moiety could be increased in [AuF_2_(OTeF_5_)​(SIMes)].^179^

The compound Et_3_SiOTeF_5_ was observed only as a side product in the reaction of [H-C_7_H_8_]​[Al­(OTeF_5_)_4_] with Et_3_SiH and was not isolated.^56^ Another example of a monoteflate substituted silane is Ph_3_SiOTeF_5_. It was formed by using Ph_3_SiCl and TlOTeF_5_.^144^ Upon reaction of Ph_3_SiH and the hydride abstractor [CPh_3_]​[B­(OTeF_5_)_4_] instead of the expected [SiPh_3_]^+^, Ph_3_SiOTeF_5_ is formed.^144^

For the selenate group only one species was published so far. Contrary to the teflate analogue, Me_3_SiOSeF_5_ was not formed by reacting the corresponding chlorosilane, but rather the silylamine (Me_3_Si)_3_N with the selenate transfer reagent HOSeF_5_.^94^

### 6.3 Germanium, Tin, and Lead Teflates

Me_3_GeOTeF_5_ is the only literature-known germanium teflate to date.^19^ The tin compound Me_3_SnOTeF_5_ is one of only two known tin teflates. According to Sladky et al., contrary to its lighter homologues, its synthesis is possible not only by reacting Me_3_ECl (E = Si, Ge, Sn) with a teflate transfer reagent but also through the reaction of Me_4_Sn with HOTeF_5_.^19^ However, a later study showed that the first approach mainly produces Me_2_SnClOTeF_5_ and Me_3_SnOTeF_5_ is the minor product.^180^ Moreover, the two solvent adducts [Me_3_­Sn­OTeF_5_­((CD_3_)_2_CO)] and [Me_3_SnOTeF_5_(CD_3_CN)] are reported.^180^

No synthesis of teflate-based lead compounds has so far been reported. Group 14 teflates and selenates are gathered in Table 5.^181–183^

**Table 5:**
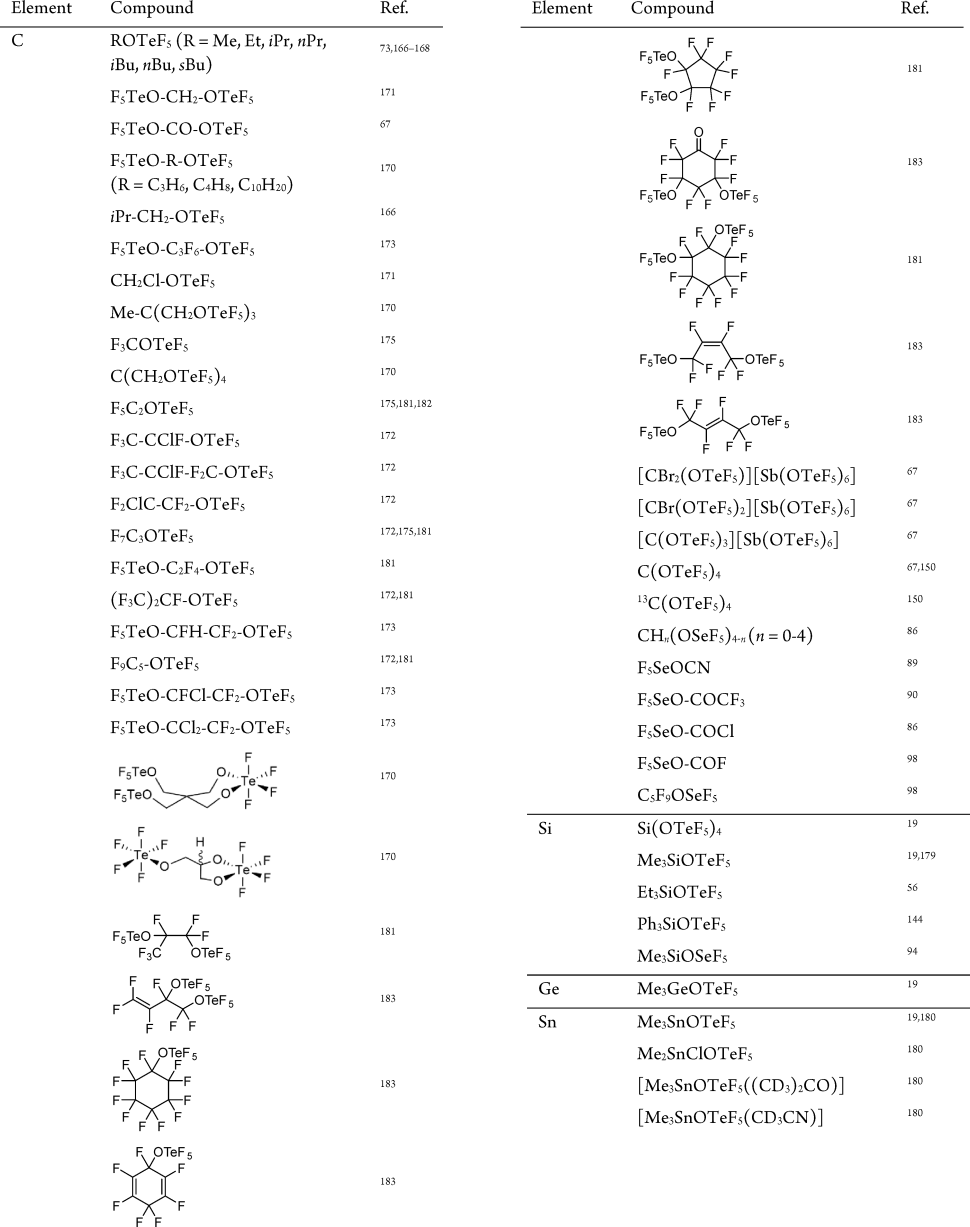
Overview of Group 14 Teflate and Selenate Compounds

## 7 Group 15 Teflates

### 7.1 Nitrogen Teflates

The only reported compound containing the N-OTeF_5_ fragment is the nitrosyl teflate, which is obtained from NOCl and Hg­(OTeF_5_)_2_.^184^ The covalent bond between the nitrogen and the teflate group can only be observed in the gas phase using IR spectroscopy. In the solid state or in solution, the molecule ionizes to form [NO]​[OTeF_5_] (see eq 16). This structural property has also been described for the related selenium compound NO_2_(OSeF_5_).^18,23^


17 Hg(OTeF 5)2+2NOCl→−HgCl22[NO][OTeF 5](s)⇌2NO(OTeF 5)(g)

### 7.2 Phosphorus Teflates

The phosphorus­(III) teflate P­(OTeF_5_)_3_ is prepared from PCl_3_ and Hg­(OTeF_5_)_2_ in decent yields but slowly decomposes at room temperature (see Scheme 5).^15^ The low stability of this compound is attributed to its inner redox potential, which can be observed through the formation of an elemental tellurium mirror over time.

**Scheme 5:**
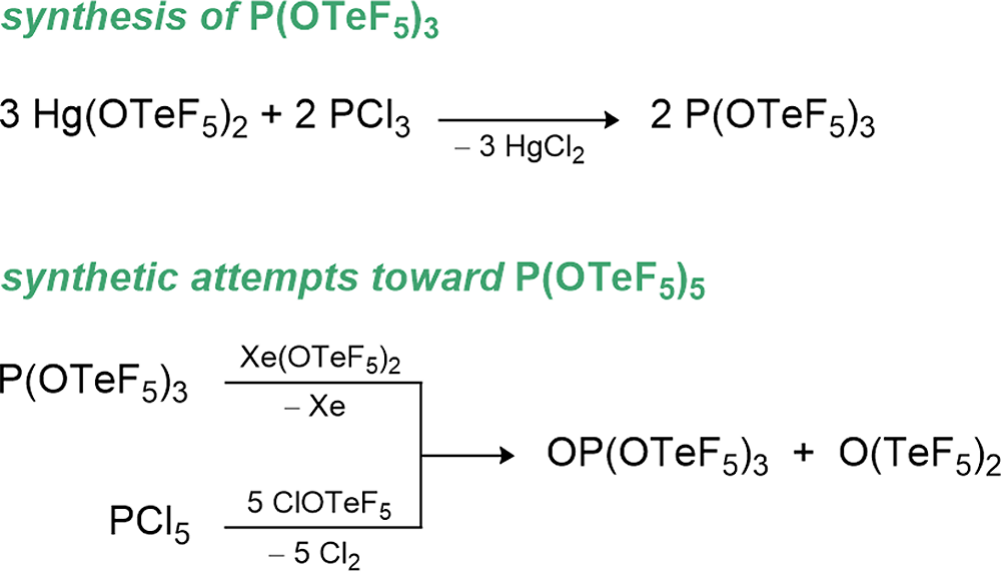
Synthetic Routes for P­(OTeF_5_)_3_ and OP­(OTeF_5_)_3_

All efforts to obtain the phosphorus­(V) teflate using different synthetic pathways such as the oxidation of the P­(III) compound with Xe­(OTeF_5_)_2_ or the ligand exchange of PCl_5_ with ClOTeF_5_ failed. In both cases ligand elimination from P­(OTeF_5_)_5_ could not be suppressed, giving exclusively the phosphorus­(V) oxo-teflate OP­(OTeF_5_)_3_ and O­(TeF_5_)_2_ as the products (see Scheme 5). Mixed fluoro-oxo-teflate P­(V) compounds are selectively synthesized by reacting POF_2_Cl with Hg­(OTeF_5_)_2_ yielding OPF_2_(OTeF_5_).^15^ In an analogous way using Hg­(OSeF_5_)_2_ the lighter OPF_2_(OSeF_5_) can be obtained.^18^

Cationic phosphonium­(V) compounds containing the OTeF_5_ group have been shown to form via the decomposition of [MePF_3_]​[Al­(OTeF_5_)_4_] in SO_2_.^73^ The cationic species [MePF_2_(OTeF_5_)]^+^ and [MePF­(OTeF_5_)_2_]^+^ have been observed by NMR spectroscopy. However, they have not been isolated.

### 7.3 Arsenic Teflates

The first arsenic teflate was prepared in 1973 by the reaction of AsF_3_ with Me_3_SiOTeF_5_ stating that initially, the monosubstituted AsF_2_(OTeF_5_) is formed, which then dismutates upon distillation to AsF_3_ and As­(OTeF_5_)_3_.^19^ The characterization of As­(OTeF_5_)_3_ was then limited to the boiling point only. Further characterization and an improved synthesis of As­(OTeF_5_)_3_ were published a few years later by Seppelt et al. in 1977. The procedure utilizes AsF_3_ and B­(OTeF_5_)_3_ as the teflate transfer reagent to obtain analytically pure As­(OTeF_5_)_3_ (see Scheme 6).^18^ In contrast to its lighter homologue As­(OSeF_5_)_3_, which decomposes over time to [SeF_3_]​[AsF_6_], the dimeric (OSeF_4_)_2_ and further unidentified products, the As­(III) compound As­(OTeF_5_)_3_ is stable over a long period of time. This difference in stability can be attributed to the higher oxidation power of Se­(VI) compared to Te­(VI) leading to intramolecular redox reactions in As­(OSeF_5_)_3_, which do not take place in As­(OTeF_5_)_3_.^18^

**Scheme 6:**
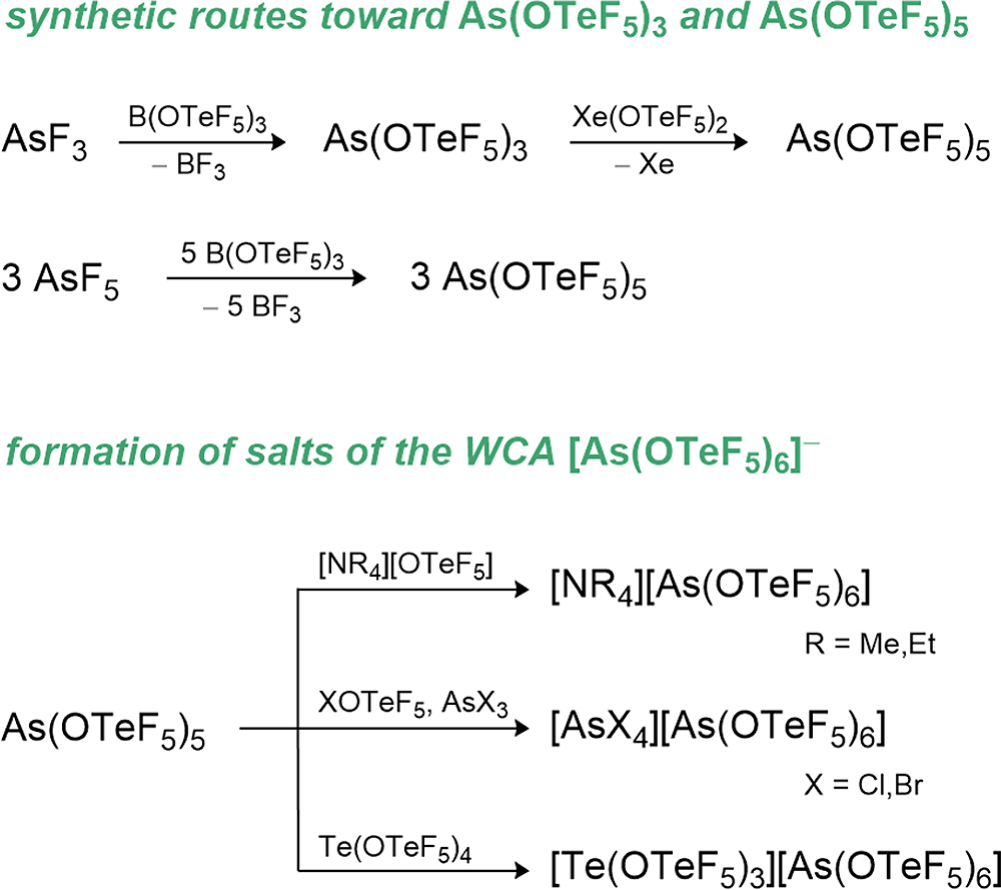
Overview of Arsenic-Based Teflate Compounds

Despite the free electron pair on As­(OTeF_5_)_3_, the strong electron-withdrawing property of the teflate group leads to an increased Lewis acidity comparable with that of AsF_3_. As­(OTeF_5_)_3_ forms adducts with simple Lewis bases like MeCN, Cl^–^ and [OTeF_5_]^−^ exhibiting σ acidity.^185^ Furthermore, As­(OTeF_5_)_3_ binds to low valent transition metals as shown in W­(CO)_5_(As­(OTeF_5_)_3_) exhibiting π acidity.^185^ The Lewis acidity drastically increases by oxidizing the As­(III) center to As­(V). With a FIA value of 580 kJ mol^–1^ for As­(OTeF_5_)_5,_ it is considered the only neutral arsenic-based Lewis superacid,^186^ surpassing other strong arsenic-based Lewis acids like AsF_5_ (439 kJ mol^–1^) and the threshold for Lewis superacidity SbF_5_ (496 kJ mol^–1^).^52,53^

The synthesis of As­(OTeF_5_)_5_ is achieved by oxidation of As­(OTeF_5_)_3_ with Xe­(OTeF_5_)_2_ (see Scheme 6).^15^ This reaction however yields, besides the desired As­(V) compound, unidentified side products, which could not be separated. An improved synthesis starts from AsF_5_ as an As­(V) precursor and stoichiometric amounts of B­(OTeF_5_)_3_ in SO_2_ and results in pure As­(OTeF_5_)_5_ (see Scheme 6).^57^

The understoichiometric use of B­(OTeF_5_)_3_ yields the mixed fluoro-teflate compound AsF­(OTeF_5_)_4_. Both Lewis acids, As­(OTeF_5_)_5_ and AsF­(OTeF_5_)_4_, have been used in the synthesis of the tetrahalopnictogen­(V) salts [AsX_4_]​[As­(OTeF_5_)_6_] and [AsX_4_]​[AsF­(OTeF_5_)_5_] (X = Cl, Br) (see Scheme 6).^58^ In the oxidation reaction of AsX_3_ with the corresponding hypohalite XOTeF_5_, the Lewis acids As­(OTeF_5_)_5_ or AsF­(OTeF_5_)_4_ act as [OTeF_5_]^−^ anion acceptors forming the respective weakly coordinating anions [As­(OTeF_5_)_6_]^−^ or [AsF­(OTeF_5_)_5_]^−^. Further studies on the [OTeF_5_]^−^ anion acceptor properties of As­(OTeF_5_)_5_ in the reaction with Te­(OTeF_5_)_4_ lead to the observation of the tellurium­(IV) salt [Te­(OTeF_5_)_3_]​[As­(OTeF_5_)_6_] in solution (see Scheme 6).^57^ However, no full conversion and isolation of the product could be achieved.

Regarding the stability of the weakly coordinating arsenate anions, the homoleptic [As­(OTeF_5_)_6_]^−^ anion shows the highest resistance against nucleophilic attacks compared to [AsF­(OTeF_5_)_5_]^−^ and [AsF_6_]^−^.^58^

The [As­(OTeF_5_)_6_]^−^ anion has been subject of detailed vibrational and multinuclear NMR spectroscopic studies with the noteworthy clear observation of the quadrupolar ^75^As core.^44,187^ Compared to the neutral As­(OTeF_5_)_5_, which does not produce an observable signal in the ^75^As NMR spectrum, the highly symmetric octahedral environment around the arsenic center in the [As­(OTeF_5_)_6_]^−^ anion, leads to well-resolved ^75^As NMR spectra of the cesium and ammonium salts even depicting the spin coupling to the ^125^Te isotope present in the teflate group as ^125^Te satellites. The studied salts have been synthesized by reaction of the Lewis acid As­(OTeF_5_)_5_ and the corresponding teflate salt [cat]​[OTeF_5_] (cat = Cs^+^, [NMe_4_]^+^).^44,57^

### 7.4 Antimony Teflates

The first antimony teflates were mentioned in 1977 by Seppelt et al.^18^ In his report he describes the reaction of SbF_5_ and B­(OTeF_5_)_3_ with a stoichiometry of 1:2, which does not lead to the desired Sb­(V) compound Sb­(OTeF_5_)_5_ but rather yields the Sb­(III) compound Sb­(OTeF_5_)_3_ via the elimination of the peroxide F_5_TeOOTeF_5_. He later revised this claim after synthesizing analytically pure Sb­(OTeF_5_)_3_ from SbF_3_ and B­(OTeF_5_)_3_.^15^ Similar to the lighter As­(III) homologue, Sb­(OTeF_5_)_3_ has a Lewis acidic character and acts as a [OTeF_5_]^−^ anion acceptor. Upon reaction with ammonium teflates [NR_4_]​[OTeF_5_] the corresponding Sb­(III) salts [NR_4_]​[Sb­(OTeF_5_)_4_] (R = Me, Et) are obtained.^44^

Despite great efforts, the successful synthesis of Sb­(OTeF_5_)_5_, often being considered one of the strongest neutral Lewis acids achievable (FIA value of 623 kJ mol^–1^),^52^ has not been realized. Only mixed fluoro-teflate Sb­(V) compounds SbF_5‑*n*_(OTeF_5_)_*n*_ (*n* = 1, 2) have been reported in stoichiometric reactions of SbF_5_ with B­(OTeF_5_)_3_ (see Scheme 7).^188^

**Scheme 7:**
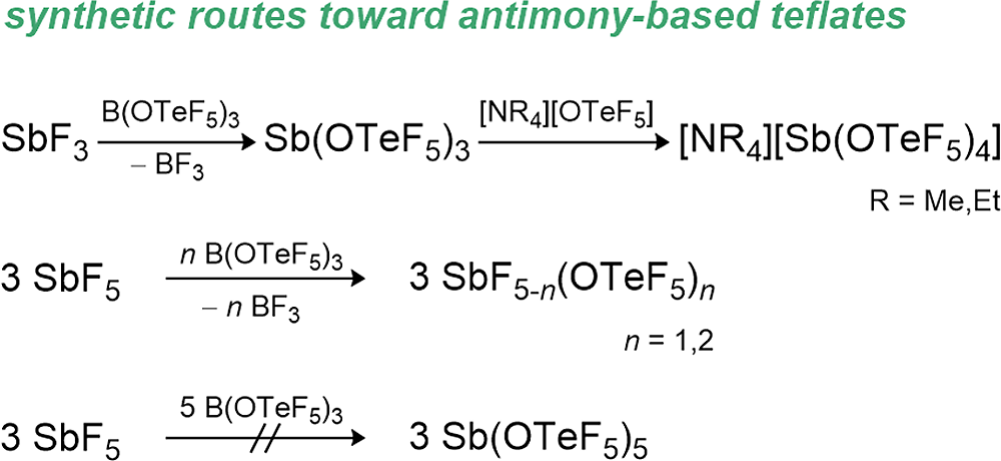
Syntheses of Sb­(OTeF_5_)_3_, the Anion [Sb­(OTeF_5_)_4_]^−^, and the Mixed SbF_5‑*n*_(OTeF_5_)_*n*_ with *n* = 1, 2

It comes as no surprise that the corresponding Sb­(V) teflate anion [Sb­(OTeF_5_)_6_]^−^ has proven itself to be an excellent weakly coordinating anion, resisting and stabilizing highly oxidizing and strongly electrophilic cations. The [Sb­(OTeF_5_)_6_]^−^ anion has been subject to detailed vibrational and multinuclear NMR spectroscopy.^44^

Synthesis of the [Sb­(OTeF_5_)_6_]^−^ anion can be achieved by a ligand exchange reaction starting from a Sb­(V) precursor. Antimony pentachloride SbCl_5_ reacts with AgOTeF_5_ under the elimination of AgCl to give the synthetically useful silver salt Ag­[Sb­(OTeF_5_)_6_] (see Scheme 8).^189^ Via simple metathesis reactions the silver antimonate salt is converted to the cesium salt Cs­[Sb­(OTeF_5_)_6_][Bibr ref190] and the trityl salt [CPh_3_]​[Sb­(OTeF_5_)_6_].^189^ Reactions of Ag­[Sb­(OTeF_5_)_6_] with the elemental chalcogens sulfur and selenium in SO_2_ gave the respective salts [Ag­(S_8_)_2_]​[Sb­(OTeF_5_)_6_][Bibr ref191] or [Ag_2_Se_6_(SO_2_)_2_]​[Sb­(OTeF_5_)_6_]_2_.^192^ The undistorted structure of the [Ag­(S_8_)_2_]^+^ cation in the solid state showcases the weakly coordinating nature of the [Sb­(OTeF_5_)_6_]^−^ anion, as the cation displays significant distortion in the chemically related salt [Ag­(S_8_)_2_]​[SbF_6_], originating from cation–anion interactions. Reactions of the silver antimonate salt with elemental tellurium in the presence of TeBr_4_ led to the dicationic [Te_4_]^2+^ species, while the reaction of Ag­[Sb­(OTeF_5_)_6_] with TeBr_4_ yields the tellurium­(IV) cation [TeBr_3_]^+^ (see Scheme 8).^193^

**Scheme 8:**
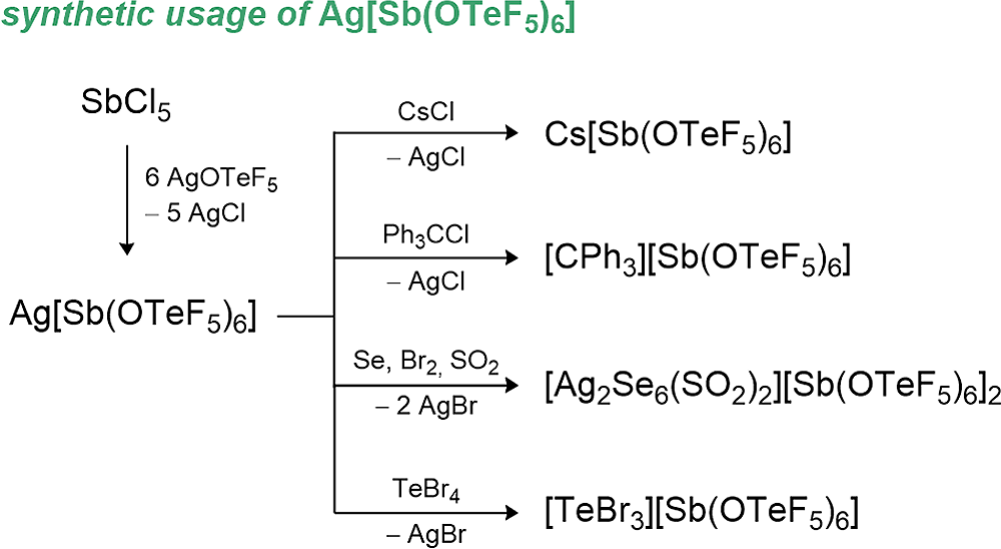
Synthesis and Reactivity of Ag­[Sb­(OTeF_5_)_6_]

Simple ammonium salts [NR_4_]​[Sb­(OTeF_5_)_6_] (R = Me, Et, *n*Bu) are obtained by the metathesis reaction of Ag­[Sb­(OTeF_5_)_6_] with ammonium chlorides [NR_4_]Cl (R = *n*Bu)^189^ or by the oxidation of the Sb­(III) salts [NR_4_]​[Sb­(OTeF_5_)_4_] (R = Me, Et) with Xe­(OTeF_5_)_2_.^44^

Oxidation of the Sb­(III) teflate Sb­(OTeF_5_)_3_ with elemental chlorine or bromine leads to ligand scrambling and the formation of the respective tetrahalostibonium­(V) salts [SbX_4_]​[Sb­(OTeF_5_)_6_] (X = Cl, Br).^194^

The [Sb­(OTeF_5_)_6_]^−^ anion shows a high stability against these strongly oxidizing cations with its salts being indefinitely stable at room temperature. Furthermore, the weakly coordinating nature of the [Sb­(OTeF_5_)_6_]^−^ anion leads to an almost perfectly tetrahedral structure of the [SbX_4_]^+^ cation (X = Cl, Br) in the solid state with the anion and the cation being well separated with only weak interactions (see Figure 13). Compared to the three other anions [Sb_2_F_11_]^−^, [Sb_2_Cl_0.5_F_10.5_]^−^ and [Sb_2_Cl_2_F_9_]^−^, which have been reported to stabilize the [SbCl_4_]^+^ cat­ion,^195–197^ the [Sb­(OTeF_5_)_6_]^−^ anion is the weakest coordinating one in this group.

**Figure 13:**
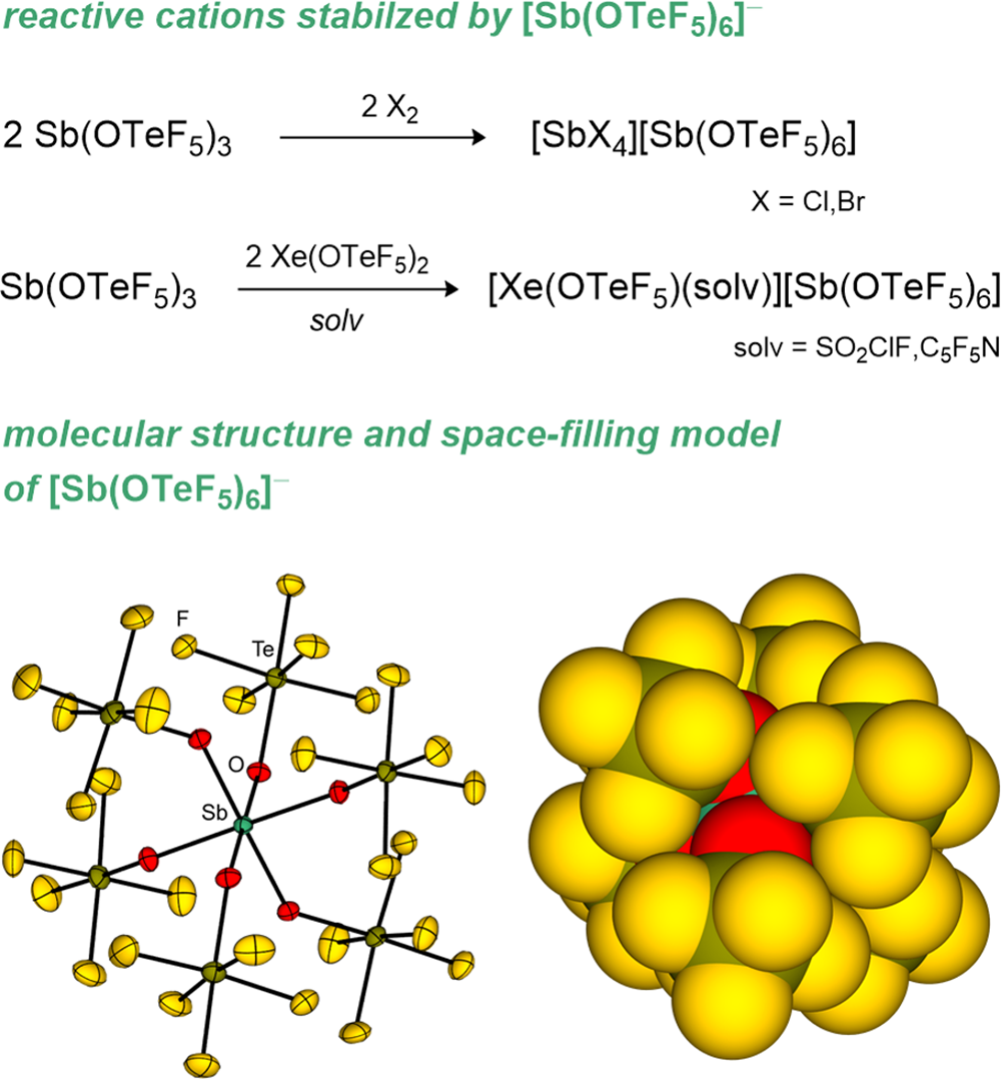
Examples of reactive cations stabilized by the simultaneous formation of [Sb­(OTeF_5_)_6_]^−^ (upper chart). Molecular structure (lower chart) in the solid state of [Sb­(OTeF_5_)_6_]^−^ (left) and the space-filling representation (right).^70^ Cations are omitted for clarity.

Another strongly oxidizing salt, the noble gas compound [Xe­(OTeF_5_)​(SO_2_ClF)]​[Sb­(OTeF_5_)_6_], can be obtained by the oxidation of the Sb­(III) teflate Sb­(OTeF_5_)_3_ with 2 equiv of Xe­(OTeF_5_)_2_.^43,69^

The structure of the salt in the solid state reveals the high Lewis acidity of the xenonium ion as it forms an adduct with the weak Lewis base SO_2_ClF. The SO_2_ClF can be displaced by other oxidation-resistant Lewis bases as shown in the synthesis of [Xe­(OTeF_5_)​(C_5_F_5_N)]​[Sb­(OTeF_5_)_6_] (see Figure 13).^70^ An excess of Xe­(OTeF_5_)_2_ in the synthesis of the xenonium salt and subsequent removal of all SO_2_ClF revealed the formation of [Xe_2_(OTeF_5_)_3_]​[Sb­(OTeF_5_)_6_] as identified by Raman spectroscopy.^69^

Furthermore, the strong oxidizing salt [Xe­(OTeF_5_)​(SO_2_ClF)]​[Sb­(OTeF_5_)_6_] finds application in the synthesis of halogenated carbocations [CX_3_]^+^, [CFX_2_]^+^ (X = Cl, Br), the teflate substituted carbocations [CBr_*n*_(OTeF5)_3‑*n*_]^+^ (*n* = 0, 1, 2) and the chloronium cations [Cl­(R)_2_]​[Sb­(OTeF_5_)_6_] (R = Me, CH_2_CF_3_).^67,68,75^ Oxidation of the hypobromite BrOTeF_5_ with [Xe­(OTeF_5_)​(SO_2_ClF)]​[Sb­(OTeF_5_)_6_] leads to the Br­(III) salt [Br­(OTeF_5_)_2_]​​[Sb­(OTeF_5_)_6_].^67^

### 7.5 Bismuth Teflates

Low oxidation state bismuth teflates are unknown; however, the neutral Bi­(V) teflate Bi­(OTeF_5_)_5_ and the bismuthate anion [Bi­(OTeF_5_)_6_]^−^ have been reported.^44^ The neutral Lewis acid Bi­(OTeF_5_)_5_ is obtained from the reaction of BiF_5_ and B­(OTeF_5_)_3_ (see eq 17). Given that Bi­(OTeF_5_)_5_ acts as a [OTeF_5_]^−^ acceptor, the synthesis of the anionic species is analogous to that of its arsenic counterpart [As­(OTeF_5_)_6_]^−^. The Bi­(V) teflate Bi­(OTeF_5_)_5_ reacts with ammonium teflate salts [NR_4_]​[OTeF_5_] yielding [NR_4_]​[Bi­(OTeF_5_)_6_] (R = Me, Et).^44^


18 BiF 5→−53BF353B(OTeF 5)3Bi(OTeF 5)5→[NR4][OTeF 5][NR4][Bi(OTeF 5)6]R=Me,Et

Comparing the color of the [E­(OTeF_5_)_6_]^−^ salts (E = As, Sb, Bi) the unique property of the bismuth compound is displayed, as [Bi­(OTeF_5_)_6_]^−^ salts are yellow, differing from the colorless [As­(OTeF_5_)_6_]^−^ and [Sb­(OTeF_5_)_6_]^−^ salts. The yellow color, which is also observed for the neutral Bi­(OTeF_5_)_5_, is believed to be a consequence of relativistic effects on the heavy Bi atom. Apart from the color, the [Bi­(OTeF_5_)_6_]^−^ anion also differs in its stability from the lighter homologues. Salts of the [Bi­(OTeF_5_)_6_]^−^ anion are stable solids, that slowly decompose in SO_2_ClF and rapidly decompose in MeCN at room temperature. ^19^F NMR spectroscopy revealed the formation of the oxo-teflate anion [OBi­(OTeF_5_)_4_]^−^ and O­(TeF_5_)_2_ and therefore a similar ligand elimination as reported for P­(OTeF_5_)_5_. Further multinuclear NMR spectroscopic investigation of the [Bi­(OTeF_5_)_6_]^−^ anion led to the observation of the extremely rare spin coupling of the ^209^Bi nuclei to another atom, in this case, the^2^ *J* coupling to the ^125^Te nuclei present in the teflate ligand.^44^ This spin coupling is observable due to the highly symmetric octahedral environment of the quadrupolar ^209^Bi center, which is also found in the [BiF_6_]^−^ anion.^198^ Group 15 teflates and selenates are gathered in Table 6.

**Table 6:** Overview of Group 15 Teflate and Selenate Compounds

Element	Ox	Compound	Refs
N	III	[NO]​[OTeF_5_]/NO(OTeF_5_)	^184^
	V	[NO_2_]​[OSeF_5_]/NO_2_(OSeF_5_)	^23^

P	III	P(OTeF_5_)_3_	^15^
	V	OP(OTeF_5_)_3_	^15^
		OPF_2_(OTeF_5_)	^15^
		OPF_2_(OSeF_5_)	^15^

As	III	As(OTeF_5_)_3_	^18^
		W(CO)_5_(As(OTeF_5_)_3_)	^185^
	V	As(OTeF_5_)_5_	^15,57^
		AsF(OTeF_5_)_4_	^58^
		[AsX_4_]​[As(OTeF_5_)_6_] (X = Cl, Br)	^58^
		[AsX_4_]​[AsF(OTeF_5_)_5_] (X = Cl, Br)	^58^
		Cs[As(OTeF_5_)_6_]	^57^
		[NMe_4_]​[As(OTeF_5_)_6_]	^44^

Sb	III	Sb(OTeF_5_)_3_	^15^
		[NMe_4_]​[Sb(OTeF_5_)_4_]	^44^
		[NEt_4_]​[Sb(OTeF_5_)_4_]	^44^
	V	SbF_4_(OTeF_5_)	^188^
		SbF_3_(OTeF_5_)_2_	^188^
		Ag[Sb(OTeF_5_)_6_]	^189^
		Cs[Sb(OTeF_5_)_6_]	^190^
		[CPh_3_]​[Sb(OTeF_5_)_6_]	^189^
		[Ag(S_8_)_2_]​[Sb(OTeF_5_)_6_]	^191^
		[Ag_2_Se_6_(SO_2_)_2_]​[Sb(OTeF_5_)_6_]_2_	^192^
		[TeBr_3_]​[Sb(OTeF_5_)_6_]	^193^
		[Cl_3_Te-F-TeCl_3_]​[Sb(OTeF_5_)_6_]	^193^
		[Te_4_]​[Sb(OTeF_5_)_6_]_2_	^193^
		[NMe_4_]​​[Sb(OTeF_5_)_6_]	^44^
		[NEt_4_]​​[Sb(OTeF_5_)_6_]	^44^
		[N(*n*Bu)_4_]​​[Sb(OTeF_5_)_6_]	^189^
		[SbX_4_]​​[Sb(OTeF_5_)_6_] (X = Cl, Br)	^194^
		[Xe(OTeF_5_)​(SO_2_ClF)]​​[Sb(OTeF_5_)_6_]	^43,69^
		[Xe(OTeF_5_)​(C_5_F_5_N)]​​[Sb(OTeF_5_)_6_]	^70^
		[Xe_2_(OTeF_5_)_3_]​​[Sb(OTeF_5_)_6_]	^69^
		[CX_3_]​​[Sb(OTeF_5_)_6_] (X = Cl, Br)	^67,68^
		[CFX_2_]​​[Sb(OTeF_5_)_6_] (X = Cl, Br)	^68^
		[C(OTeF_5_)_3_]​​[Sb(OTeF_5_)_6_]	^67,68^
		[CMe_3_]​​[Sb(OTeF_5_)_6_]	^75^
		[CEtMe_2_]​​[Sb(OTeF_5_)_6_]	^75^
		[C_6_H_11_]​[Sb(OTeF_5_)_6_]	^75^
		[Br(OTeF_5_)_2_]​[Sb(OTeF_5_)_6_]	^67^
		[ClMe_2_]​[Sb(OTeF_5_)_6_]	^75^
		[Cl(CH_2_CF_3_)_2_]​[Sb(OTeF_5_)_6_]	^75^
		[I(CH_2_CF_3_)_2_]​[Sb(OTeF_5_)_6_]	^75^
		[F_5_C_5_N(CH_2_CF_3_)]​[Sb(OTeF_5_)_6_]	^75^

Bi	V	Bi(OTeF_5_)_5_	^44^
		[NMe_4_]​[Bi(OTeF_5_)_6_]	^44^
		[NEt_4_]​[Bi(OTeF_5_)_6_]	^44^

## 8 Group 16 Teflates

### 8.1 Oxygen Teflates

The oxo compound F_5_TeOTeF_5_ can be regarded as the anhydride of teflic acid, HOTeF_5_, and is obtained either by fluorination of TeO_2_ ^100,101,199^ or the reaction of (F_5_TeO)_2_SO_2_ with KOTeF_5_ or CsCl (see Scheme 9).^4^ Structural information of F_5_TeOTeF_5_ obtained by electron diffraction measurements show a relatively large Te–O–Te bond angle (145.5°) and remarkably short O–Te bond lengths (183.2 pm).^100,101^ Interestingly, the conformation of the TeF_5_ groups is eclipsed, even though it is sterically the least favored. These structural properties of F_5_TeOTeF_5_ are attributed to a (pd)­π bonding between the tellurium and the oxygen atoms.^100,101^ Furthermore, this (pd)­π bonding is observed in the peroxide F_5_TeOOTeF_5_ leading to the remarkable stability of this compound. The O–O bond is reported to be unusually strong as judged by the high O–O valence frequencies in the Raman spectrum.^5,200^ The peroxide shows no homolytic decomposition under ambient conditions; however, it decomposes to the oxo compound F_5_TeOTeF_5_ if heated above 80 °C.^5^ The selective synthesis of the peroxide is achieved either by thermal or photolytically decomposition of the noble gas compounds XeF­(OTeF_5_), Xe­(OTeF_5_)_2_, Xe­(OTeF_5_)_4_, Xe­(OTeF_5_)_6_ or Kr­(OTeF_5_)_2_ or the photolytical decomposition of ClOTeF_5_ (see Scheme 9).^1,5,16,201,202^ On the other hand, the thermal decomposition of the lighter derivative Xe­(OSeF_5_)_2_ does not yield the peroxide but the anhydride O­(SeF_5_)_2_.^85^ The peroxide F_5_SeOOSeF_5_, however, can be synthesized by fluorination of SeO_2_.^84,99^

**Scheme 9:**
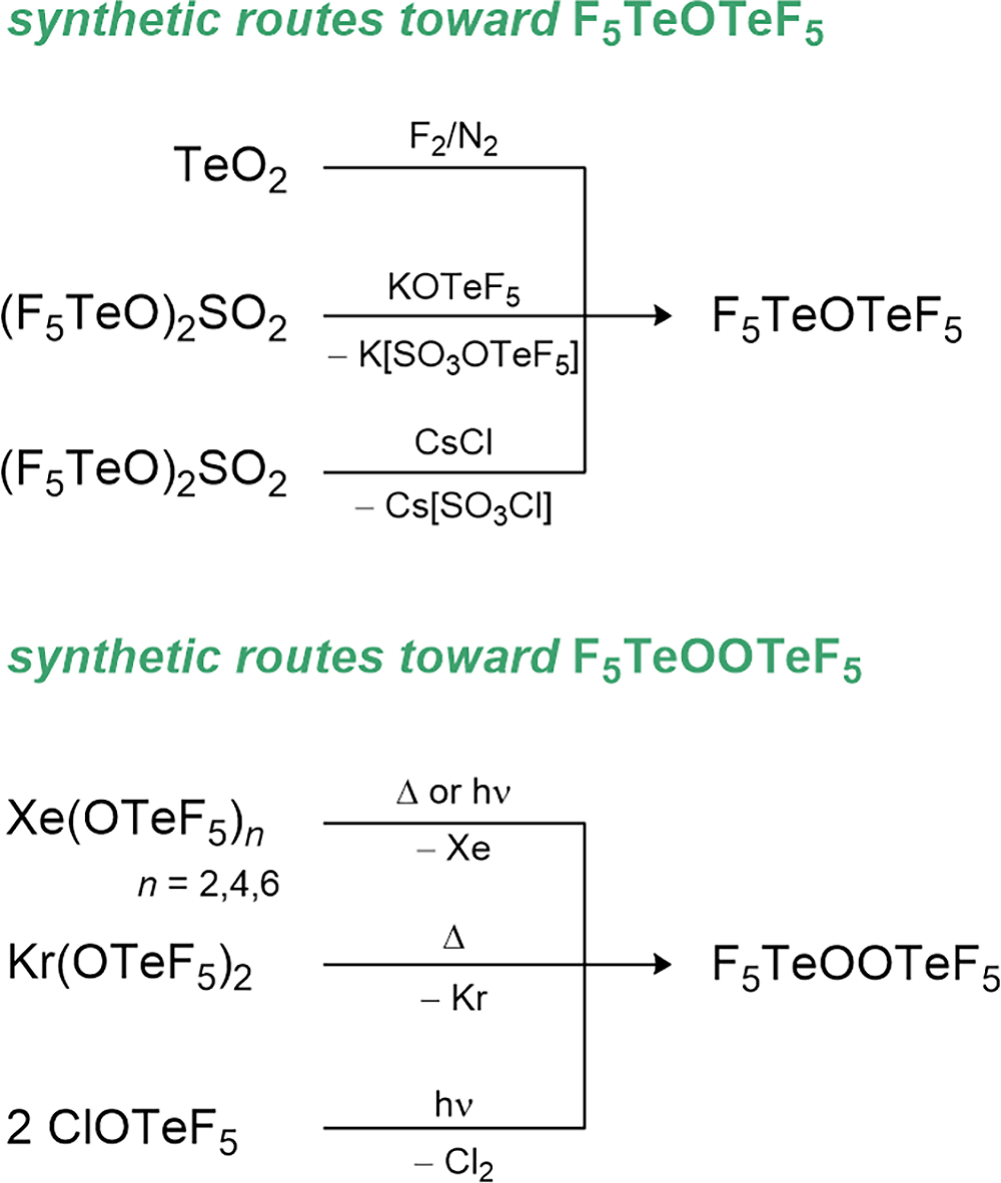
Synthesis of the Anhydride F_5_TeOTeF_5_ and the Peroxide F_5_TeOOTeF_5_

### 8.2 Sulfur Teflates

The earliest mention of sulfur teflate compounds coincides with the first reported synthesis of teflic acid HOTeF_5_. In 1964 Sladky et al. reported on the reaction of barium tellurate, BaTeO_4_, with fluorosulforic acid FSO_3_H yielding, depending on the water content of the barium tellurate, HOTeF_5_ and the three teflate-containing sulfur compounds F_5_TeOSO_2_F, (F_5_TeO)_2_SO_2_, and F_5_TeOSO_3_H in differing ratios.^3^ After their accidental discovery the three sulfur-oxo-teflates were selectively synthesized (see eqs 18–20).^4^


19 HOTeF 5→SO3F 5TeOSO3H

20 F 5TeOSO3H→−H2SO4HSO3XF 5TeOSO2XX=F,Cl

21 F 5TeOSO3H→CsCl(F 5TeO)2SO2

Additionally, F_5_TeOSO_2_Cl was synthesized from F_5_TeOSO_3_H and chlorosulforic acid ClSO_3_H.^4^ Their physical properties and their vibrational and ^19^F NMR spectra have been described in detail in the literature.^3,4,203,204^

The only other reported compound containing a S-OTeF_5_ fragment is the thiazyl-pentafluoro­orthotellurate NS-OTeF_5_, which is obtained by the reaction of the thiazyl salt [NS]​[SbF_6_] and CsOTeF_5_.^205^ Other pathways, like the reaction of NSF with B­(OTeF_5_)_3_, also yield NS-OTeF_5_; however, significant byproducts like the isomerized OSNTeF_5_ and the sulfur diimide S­(NTeF_5_)_2_ are observed.

Sulfur compounds containing the OSeF_5_ group are rare. Examples are F_5_SeO-SO_2_-OSO_2_F, F_5_SeOSF_5_ or *cis*-BrCH_2_-SF_4_-OSeF_5_, which can be prepared starting from SO_3_, SF_4_ or CH_2_SF_4_ and the corresponding hypohalite XOSeF_5_ (X = F, Br).^90,98,102^ Furthermore, the reaction of the two peroxides F_5_SeOOSeF_5_ and S_2_O_6_F_2_ yields the fluorosulfate F_5_SeO-SO_2_F.^99^

### 8.3 Selenium Teflates

Attempts to obtain the homoleptic Se­(IV) teflate Se­(OTeF_5_)_4_ from the stoichiometric reaction of SeF_4_ and B­(OTeF_5_)_3_ lead to the isolation of the oxo-teflate compound OSe­(OTeF_5_)_2_, while the use of substoichiometric amounts of B­(OTeF_5_)_3_ leads to a product mixture of OSeF­(OTeF_5_), F_2_Se­(OTeF_5_)_2_ and [SeF_3_]​[B­(OTeF_5_)_4_].^87^ So far, these are the only reported selenium teflate species. The lighter homologues containing the OSeF_5_ group are only known for the oxo and difluoro selenium compounds OSe­(OSeF_5_)_2_ and F_2_Se­(OSeF_5_)_2_.^23,87^

### 8.4 Tellurium Teflates

The accidental discovery^18^ of *trans*-TeF_2_(OTeF_5_)_4_ gave rise to the systematic synthesis of a great variety of homoleptic tellurium teflate and heteroleptic tellurium fluorido teflate compounds.

The homoleptic Te­(IV) teflate Te­(OTeF_5_)_4_ is obtained by the stoichiometric reaction of TeF_4_ and B­(OTeF_5_)_3_.^22,28,57^ Its structure was assigned via low-temperature ^125^Te NMR spectroscopy to have a pseudo trigonal bipyramidal configuration at the Te center.^32^ Oxidation of Te­(OTeF_5_)_4_ with the noble gas compound Xe­(OTeF_5_)_2_ yields the homoleptic Te­(VI) teflate Te­(OTeF_5_)_6_ (see eqs 21 and 22).^22^


22 3TeF 4→−4BF 34B(OTeF 5)33Te(OTeF 5)4

23 Te(OTeF 5)4→−XeXe(OTeF 5)2Te(OTeF 5)6

The reaction of TeF_4_ with substoichiometric amounts of B­(OTeF_5_)_3_ leads to the formation of the heteroleptic Te­(IV) fluorido teflate F_2_Te­(OTeF_5_)_2_ which can be oxidized with the hypofluoride FOTeF_5_ to obtain *mer*-F_3_Te­(OTeF_5_)_3_.^87^

The *cis*- and *trans*-isomers of F_4_Te­(OTeF_5_)_2_ are obtained as a product mixture by the oxidation of TeF_4_ with Xe­(OTeF_5_)_2_. Separation of the two isomers can be achieved either by fractional crystallization or by crystallization of the *trans*-isomer followed by the distillation of the *cis*-isomer.^22,28^

If Te­(OTeF_5_)_4_ is reacted with XeF_2_ the main product of the reaction is the *cis*-isomer of F_2_Te­(OTeF_5_)_4_, while the reaction with diluted fluorine leads to a *cis*- and *trans*-isomer mixture of F_2_Te­(OTeF_5_)_4_. In both cases, FTe­(OTeF_5_)_5_ is formed as a side product, which can be isolated by fractional crystallization.^22,28^

The Te­(IV) compound Te­(OTeF_5_)_4_ can act as a OTeF_5_ group or fluoride ion acceptor, forming the anionic [Te­(OTeF_5_)_5_]^−^ or [TeF­(OTeF_5_)_4_]^−^.^206^ On the other hand Te­(OTeF_5_)_4_ is able to act as an OTeF_5_ group donor toward strong Lewis acids like AsF_5_, forming the mixed teflate fluoride cations [TeF_*n*_(OTeF_5_)_*n*−3_]^+^ with *n* = 0–3.^57^ Group 16 teflates and selenates are gathered in Table 7.

**Table 7:** Overview of Group 16 Teflate and Selenate Compounds

Element	Ox	Compound	Refs
O	I	F_5_TeOOTeF_5_	^1,5,16,201,202^
	II	O(TeF_5_)_2_	^4,199^
	I	F_5_SeOOSeF_5_	^84,99^
	II	O(SeF_5_)_2_	^85^

S	IV	NS-OTeF_5_	^205^
	VI	F_5_TeOSO_2_F	^3,4^
		F_5_TeOSO_2_Cl	^4^
		(F_5_TeO)_2_SO_2_	^3,4^
		F_5_TeOSO_3_H	^3,4^
		F_5_SeO-SO_2_-OSO_2_F	^90^
		F_5_SeOSF_5_	^98^
		F_5_SeO-SO_2_F	^99^
		*cis*-BrCH_2_-SF_4_-OSeF_5_	^102^

Se	IV	OSe(OTeF_5_)_2_	^87^
		F_2_Se(OTeF_5_)_2_	^87^
		OSe(OSeF_5_)_2_	^23,87^
		F_2_Se(OSeF_5_)_2_	^87^

Te	IV	Te(OTeF_5_)_4_	^22,28,57^
		[NR_4_]​[Te(OTeF_5_)_5_] (R = Me, Et)	^206^
		[NMe_4_]​[TeF(OTeF_5_)_4_]	^206^
		F_2_Te(OTeF_5_)_2_	^87^
		[TeF_*n*_(OTeF_5_)_*n*‑3_] [AsF_*m*_(OTeF_5_)_6‑*m*_] (*n* = 0–3)	^57^
	VI	Te(OTeF_5_)_6_	^22,28,32^
		TeF(OTeF_5_)_5_	^22,28^
		*cis*-F_2_Te(OTeF_5_)_4_	^22,28^
		*trans*-F_2_Te(OTeF_5_)_4_	^18,22,28,207,208^
		*mer*-F_3_Te(OTeF_5_)_3_	^87^
		*cis*-F_4_Te(OTeF_5_)_2_	^22,28^
		*trans*-F_4_Te(OTeF_5_)_2_	^22,28^

## 9 Group 17 Teflates

The whole set of hypohalites of the TeF_5_ moiety, XOTeF_5_ (X = F, Cl, Br, I), was recently investigated from a computational point of view using state-of-the-art methods of quantum chemical topology.^33^ When analyzing the QTAIM charges, it is interesting to point out that the total charge of the TeF_5_ moiety is independent of the atom X. It is in fact the O atom the most affected by the nature of X, therefore acting as a charge buffer between the X atom and the TeF_5_ fragment. This way, the charge at the O atom shifts from −0.37 au in the case of X = F to −1.01 au for X = I. As expected, the charge of the oxygen bound F atom of FOTeF_5_ is negative (−0.14 au). The other halogen atoms of the hypohalite group become increasingly positively charged (Cl = 0.29, Br = 0.40, and I = 0.61 au). The IQA (interacting quantum atoms) analysis also points to both F and OTeF_5_ having similar electron-withdrawing properties in this case. Nevertheless, when comparing this set of compounds with the corresponding XF, a stronger electron-sharing interaction in the bonding is found for the teflate analogues, whereas a stronger electrostatic contribution and a higher charge separation are found in the case of XF. The electronic distribution within the systems was additionally investigated by means of electron distribution functions (EDFs), which together with the QTAIM results, indicate the greater similarity of F and OTeF_5_ from an electronic point of view when they are bonded to less electronegative systems.

### 9.1 Hypofluorite

Synthetic attempts to prepare FOTeF_5_, which exists as a colorless gas at room temperature, can be traced back to a report of Seppelt et al. from 1973.^5^ In this work, UV light treatment of FXeOTeF_5_ and fluorination of Hg­(OTeF_5_)_2_ both failed to achieve the desired hypofluorite. A decade later, the work of Christe et al. showed that the reaction of HOTeF_5_ with [NF_4_]​[HF_2_] produced FOTeF_5_ in low yields. However, yields of approximately 70% could be achieved by reacting an excess of CsOTeF_5_ with fluorine fluorosulfate FOSO_2_F at −45 °C for 9 days (see eq 23).^6^ An improved synthesis enabled a higher yield of 94% in a much shorter reaction time of 1 day by reacting B­(OTeF_5_)_3_ with elemental fluorine in a Monel vessel heated to 115 °C, as shown in eq 24.^141^ The advantage of this method is the ease of isolating FOTeF_5_ from the side products, namely BF_3_ and HOTeF_5_, by fractional condensation.


24 CsOTeF 5+FOSO2F→FOTeF 5+CsSO3F

25 B(OTeF 5)3+3F 2→3FOTeF 5+BF 3

In 1956, Dudley, Cady, and Eggers described the formation of pentafluorosulfur hypofluorite (FOSF_5_) through the reaction of fluorine with SO_2_F in the presence of AgF_2_ at 200 °C.^123^ Later, electron diffraction studies revealed that the sulfur atom in FOSF_5_ is octahedrally coordinated.^209^

Building on this work, Cady et al. turned their attention to the fluorination of SeO_2_ and SeOCl_2_. In 1959, they reported the formation of pentafluoroselenium hypofluorite (FOSeF_5_) in small yields by reacting these selenium compounds with fluorine in the presence of AgF_2_.^84^ This process was later improved in 1970 when Cady et al. described a more efficient synthesis. In this method, SeOF_2_ was reacted with KF to produce KSeOF_3_, which was then fluorinated to yield FOSeF_5_.^98^ With advancements in OSeF_5_ chemistry, Seppelt reported a new approach in 1973 to synthesize FOSeF_5_ by reacting Hg­(OSeF_5_)_2_ with fluorine.^85^

### 9.2 Hypochlorite

The hypochlorite ClOTeF_5_, was quantitatively obtained by reacting Hg­(OTeF_5_)_2_ with chlorine monofluoride ClF (see eq 25) as shown by Seppelt et al. in 1973.^5^ This compound exists as a yellow liquid at room temperature and its hydrolysis products, Cl_2_O and HOTeF_5_, indicate the positive charge character of the chlorine in ClOTeF_5_ (see Figure 14).


26 Hg(OTeF 5)2+2ClF→2ClOTeF 5+HgF 2

**Figure 14:**
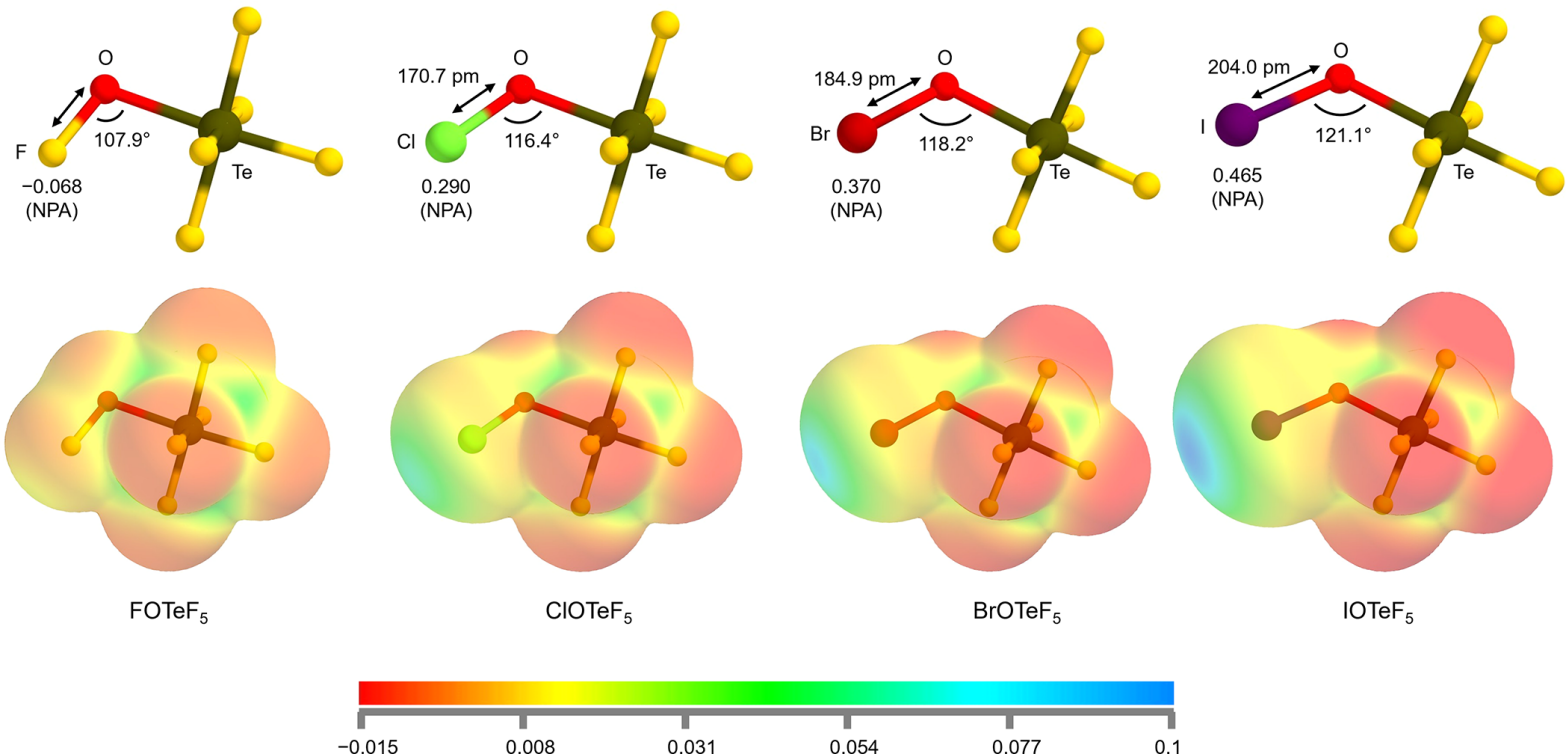
Optimized structures and NPA charges of the hypohalites FOTeF_5_, ClOTeF_5_, BrOTeF_5_, IOTeF_5_ as well as the calculated electrostatic potential plotted on the electron density (BP86/def2-SVP level, isovalue: 0.025 e bohr^–3^) using ORCA 5.0.4,^80^ visualized with Chemcraft.^81^

Another approach is to react B­(OTeF_5_)_3_ with ClF, forming ClOTeF_5_ and the side product BF_3_.^27^ The high volatility of BF_3_ allows for a more straightforward isolation of ClOTeF_5_ (see eq 26). Christe et al. reported that reacting HOTeF_5_ with either ClOSO_2_F or ClF allows direct access to ClOTeF_5_, eliminating the need for teflate transfer reagents used in previous methods, as shown in eq 27.^210^


27 B(OTeF 5)3+3ClF→3ClOTeF 5+BF 3

28 HOTeF 5+ClX→ClOTeF 5+HXX=F,OSO2F

In 1969, Gould et al. and Schack et al. independently reported the formation of pentafluorosulfur hypochlorite (ClOSF_5_) through the reaction of SOF_4_ with ClF in the presence of CsF.^112,211^ Later, Kornath et al. investigated the vibrational spectra and gas-phase structure of ClOSF_5_. They synthesized the compound using a similar method, starting from SOF_2_, which yielded an almost quantitative formation of ClOSF_5_.^212^

In 1973, Seppelt reported the formation of pentafluoro­selenium hypochlorite (ClOSeF_5_) by the reaction of Hg­(OSeF_5_)_2_ with ClF.^85^

### 9.3 Hypobromite and Bromine Teflates

The bromine derivative BrOTeF_5_, which appears as a ruby red liquid at room temperature, can be synthesized by reacting ClOTeF_5_ with bromine (see eq 28).^23^ The compound remains stable when handled at −20 °C and can also be stored in dry CFCl_3_. However, any water present in the solution will hydrolyze BrOTeF_5_, generating HOTeF_5_ and precipitating Br_2_O.^213^ The selenate derivative BrOSeF_5_ is synthesized by reacting elemental bromine with either FOSeF_5_ (62% yield) or Br­(OSeF_5_)_3_ (94% yield).^85^


29 2ClOTeF 5+Br 2→2BrOTeF 5+Cl2

A teflate compound of bromine in an oxidation state higher than +I was reported for the bromonium­(III) cation in the salt [Br­(OTeF_5_)_2_]​[Sb­(OTeF_5_)_6_]. This salt is obtained by reacting [Xe­(OTeF_5_)]​[Sb­(OTeF_5_)_6_] with a stochiometric excess of BrOTeF_5_ in SO_2_ClF at −78 °C, as shown in eq 29.^67^ Seppelt reported a bromine oxidation state of +III for the neutral compound Br­(OSeF_5_)_3_ by reacting BrF_3_ with 3 equiv of HOSeF_5_,^85^ and as an anion in the salt Rb­[Br­(OSeF_5_)_4_] upon reacting the neutral compound with Rb­[OSeF_5_].^23^


30 [Xe(OTeF 5)][Sb(OTeF 5)6]+BrOTeF 5→[Br(OTeF 5)2][Sb(OTeF 5)6]+Xe

A bromine­(V) compound stabilized by a teflate group was prepared from the controlled ozonization of BrOTeF_5_ in CFCl_3_ at low temperatures. This process results in the formation of O_2_Br­(OTeF_5_), which yields colorless crystals upon recrystallization from CH_2_Cl_2_ (see eq 30 and Figure 15).


31 BrOTeF 5+O3→O2BrOTeF 5

**Figure 15:**
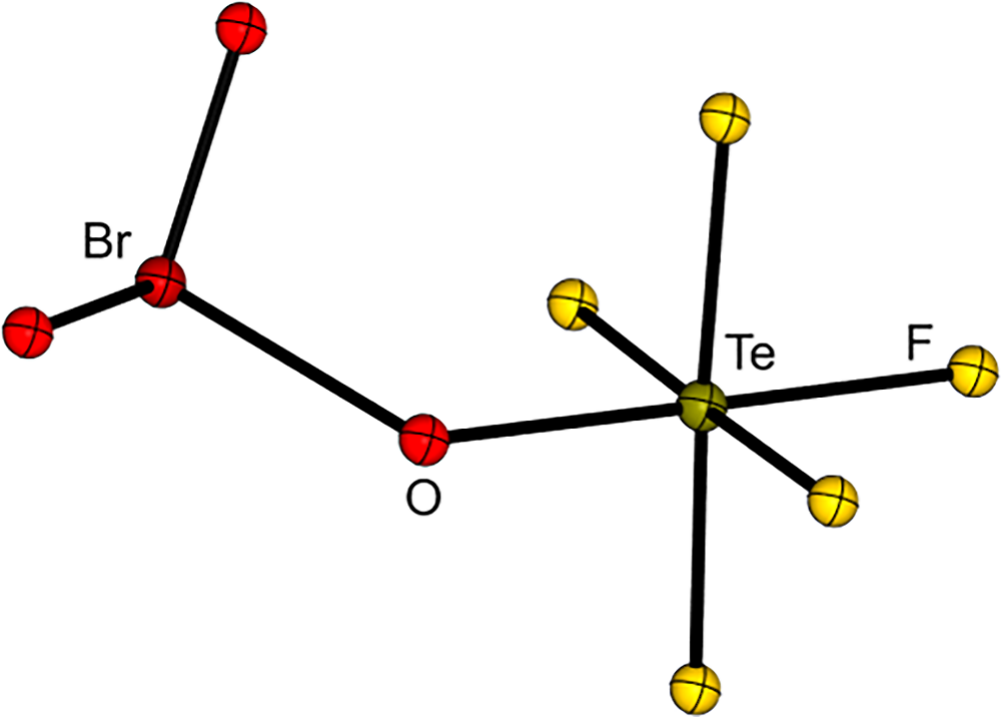
Molecular structure of O_2_Br­(OTeF_5_) in the solid state.^213^

Partial decomposition occurs at temperatures above its melting point of −20 °C, but unlike other BrO_2_ compounds such as Br_2_O_5_ and FBrO_2_, it does not exhibit an explosive behavior.^213^ This increased stability is attributed to the presence of the electron withdrawing OTeF_5_ group.

### 9.4 Iodine Teflates

Before the discovery of iodine compounds with teflate groups, the selenium derivatives IOSeF_5_ and I­(OSeF_5_)_3_ were known to be unstable solids that decompose readily via autoredox pathways.^85^ On the other hand, the greater stability of hexavalent tellurium made it possible to synthesize stable iodine compounds.

The formation of the hypoiodite compound IOTeF_5_ as a brown solid was reported through various methods. However, characterization proved difficult because it readily converts into I­(OTeF_5_)_3_ and other side products, as shown in eqs 31–33.^23^


32 I(OTeF 5)3+I2⇌3IOTeF 5

33 ClOTeF 5+ICl→I(OTeF 5)3→IOTeF 5+ICl 3+Cl2

34 BrOTeF 5+I2→I(OTeF 5)3→IOTeF 5+IBr+Br 2

The neutral iodine­(III) teflate compound, I­(OTeF_5_)_3_, is obtained from the reaction of ICl_3_ with ClOTeF_5_ in CFCl_3_ (see eq 34).^23^ Subsequent crystallization attempts yielded orange crystals identified as the decomposition product ClI­(OTeF_5_)_2_, formed by the slow reaction with CFCl_3_ (see eq 35). The molecular structure of ClI­(OTeF_5_)_2_ reveals a T-shaped coordination at the iodine center, although the {ClIO_2_} skeleton is not planar due to the steric repulsion of the OTeF_5_ groups. ClI­(OTeF_5_)_2_ is also produced by reacting I_2_ with IF_5_ and B­(OTeF_5_)_3_ in CFCl_3_ at room temperature (see eq 36). By occasionally pumping off BF_3_ and slowly removing volatile components, the compound is formed as a red liquid, which crystallizes slowly at room temperature.^214^


35 3ClOTeF 5+ICl3→I(OTeF 5)3+3Cl2

36 I(OTeF 5)3→CFCl3ClI(OTeF 5)2

37 I2+IF 5+exc.B(OTeF 5)3→CFCl3ClI(OTeF 5)2+BF 3

The reaction of I­(OTeF_5_)_3_ with [N­(*n*Bu)_4_]​[OTeF_5_] leads to the formation of the iodine­(III) anion [I­(OTeF_5_)_4_]^−^. In the molecular structure of the salt [N­(*n*Bu)_4_]​[I­(OTeF_5_)_4_] the anion adopts a nearly square planar geometry around the iodine atom (see Figure 16).^214^

**Figure 16:**
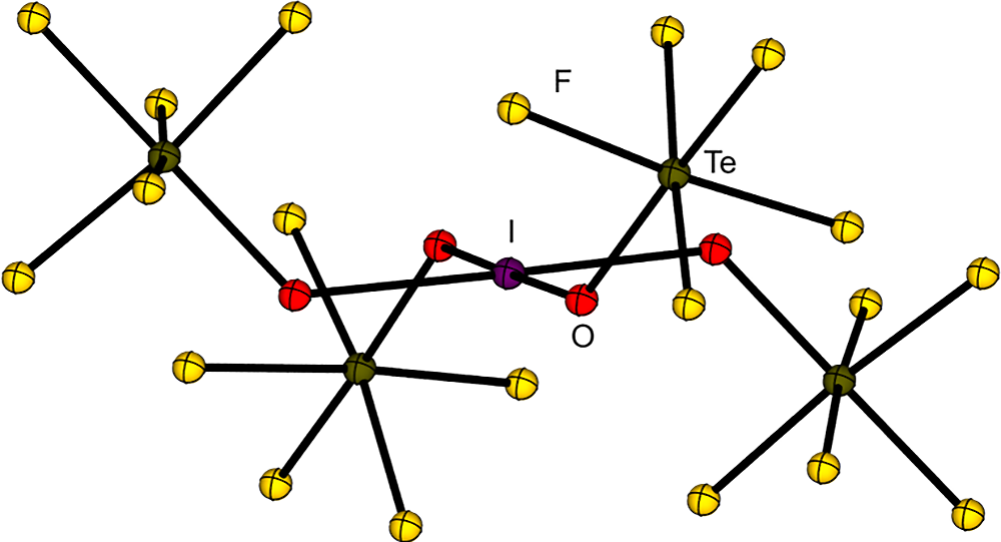
Molecular structure of the [I­(OTeF_5_)_4_]^−^ anion in the solid state.^214^ The [N­(*n*Bu)_4_]^+^ cation is omitted for clarity.

The reaction of fluorocarbon iodides with teflate transfer reagents results in the formation of R^F^I­(OTeF_5_)_2_ (R^F^ = CF_3_, C_2_F_5_, *n*-C_3_F_7_, *i*-C_3_F_7_) species. Naumann et al. reported the ligand exchange reaction of CF_3_IF_2_ with HOTeF_5_, resulting in the formation of CF_3_I­(OTeF_5_)_2_. For the complete conversion, an excess of HOTeF_5_ in MeCN is necessary.^215^ Later, Christe et al. demonstrated that ClOTeF_5_ can also be used to access R^F^I­(OTeF_5_)_2_ species (see eq 37). Except for the trifluoromethyl derivative, these compounds are thermally stable at room temperature.^216^


38 RFIF 2+2ClOTeF 5→RFI(OTeF 5)2+ClFRF=CF3,C2F5,n‐C3F7,i‐C3F7

The teflate transfer reaction of B­(OTeF_5_)_3_ with IF_5_, which is known as a useful synthon for pentavalent iodine­(V) compounds, provides access to the iodine­(V) compound FI­(OTeF_5_)_4_.^21,31^ Attempts to exchange the remaining fluorido ligand with the OTeF_5_ group by adding B­(OTeF_5_)_3_ resulted in the formation of OI­(OTeF_5_)_3_ (see eq 38).^21,31^ The oxo compound OI­(OTeF_5_)_3_ can be isolated as a colorless sublimable solid that is sensitive to water. The fully teflate substituted iodine­(V) compound, I­(OTeF_5_)_5_, can be synthesized by reacting I­(OTeF_5_)_3_ with Xe­(OTeF_5_)_2_ resulting in a colorless sublimable solid, as shown in eq 39.^21,31^ Based on ^19^F NMR spectroscopy, the observation of two inequivalent teflate groups in a 1:4 ratio suggests that the compound I­(OTeF_5_)_5_ has a square pyramidal molecular geometry similar to that of IF_5_. However, I­(OTeF_5_)_5_ quickly undergoes disproportionation, yielding I­(OTeF_5_)_3_.^21^ Reacting I­(OTeF_5_)_5_ with IF_5_ results in mixed substituted fluoro-teflate iodine­(V) compounds F_*n*_I­(OTeF_5_)_5–*n*_ (*n* = 0–5) (see eq 40). The compounds F^*ax*^I­(OTeF_5_)_4_, F^*ax*^F^*eq*^I­(OTeF_5_)_3_, *cis*- and *trans*-F^*ax*^F_2_^*eq*^I­(OTeF_5_)_2_ and F^*ax*^F_3_^*eq*^I­(OTeF_5_) were characterized by ^19^F NMR spectroscopy, where each compound has a fluorine atom occupying the axial position in a square pyramidal molecular structure.^21,32^ Similar substitution was also reported by Seppelt et al. for the selenate derivatives, F_*n*_I­(OSeF_5_)_5–*n*_ (*n* = 0–5), formed by reacting IF_5_ with POF_2_OSeF_5_.^21^


39 IF 5→−43BF 343B(OTeF 5)3FI(OTeF 5)4→−BF 3−TeF 6B(OTeF 5)4OI(OTeF 5)3

40 Xe(OTeF 5)2+I(OTeF 5)3→hvI(OTeF 5)5+Xe

41 I(OTeF 5)5+IF 5⇌FnI(OTeF 5)5−nn=0−5

**Table 8:** Overview of Group 17 Teflates and Selenate Compounds

Element	Ox	Compound	Refs
F	I	FOTeF_5_	^5,6,141,172^
		FOSeF_5_	^84,85,87,98^

Cl	I	ClOTeF_5_	^5,6,210^
		ClOSeF_5_	^85^

Br	I	BrOTeF_5_	^23,58,213^
	III	[Br(OTeF_5_)_2_]​[Sb(OTeF_5_)_6_]	^67^
	V	O_2_BrOTeF_5_	^213^
	I	BrOSeF_5_	^85^
	III	Br(OSeF_5_)_3_	^85^
		Rb[Br(OSeF_5_)_4_]	^23^

I	I	IOTeF_5_	^23^
	III	I(OTeF_5_)_3_	^23^
		ClI(OTeF_5_)_2_	^214^
		R^F^I(OTeF_5_)_2_ (R^F^ = CF_3_, C_2_F_5_, *n*-C_3_F_7_, *i*-C_3_F_7_)	^215,216^
		[N(*n*Bu)_4_]​[I(OTeF_5_)_4_]	^214^
	V	F_*n*_I(OTeF_5_)_5–*n*_ (*n* = 0–5)	^21,31^
		OI(OTeF_5_)_3_	^21,31^
		[N(*n*Bu)_4_]​[OI(OTeF_5_)_4_]	^214^
	III	I(OSeF_5_)_3_	^85^
	V	F_*n*_I(OSeF_5_)_5‑*n*_	^21^

In contrast to the iodine­(III) anion [I­(OTeF_5_)_4_]^−^, attempts to form [N­(*n*Bu)_4_]​[I­(OTeF_5_)_6_] by reacting I­(OTeF_5_)_5_ with [N­(*n*Bu)_4_]​[OTeF_5_] were unsuccessful, even with careful screening of the reaction conditions. Instead, the hydrolyzed product [N­(*n*Bu)_4_]​[OI­(OTeF_5_)_4_] was obtained and characterized in the solid state. The [OI­(OTeF_5_)_4_]^−^ anion was found to have a pyramidal geometry with the oxygen atom at the apical position (see Figure 17).^214^

**Figure 17:**
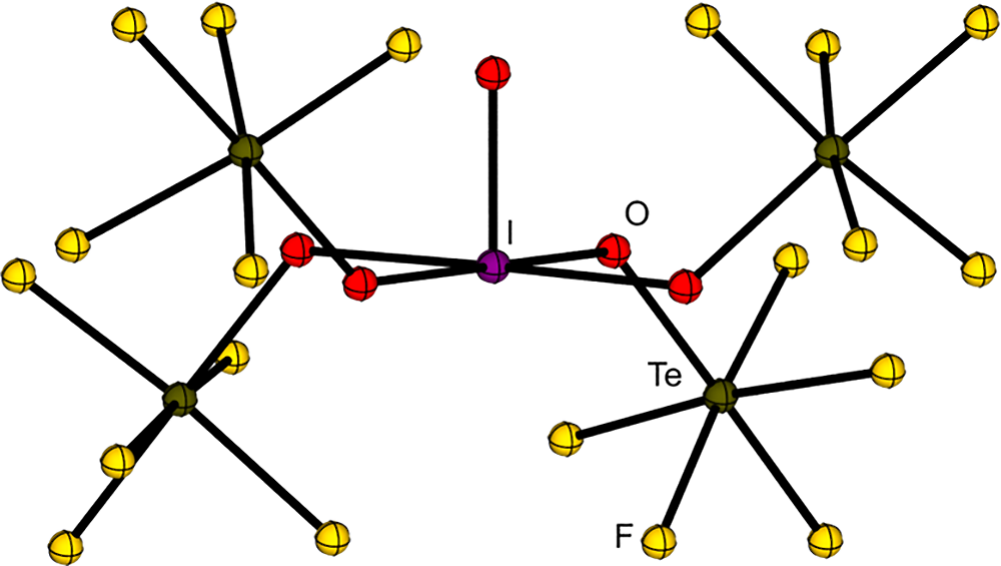
Molecular structure of the [OI­(OTeF_5_)_4_]^−^ anion in the solid state.^214^ The [N­(*n*Bu)_4_]^+^ cation is omitted for clarity.

Efforts to achieve an even higher oxidation state with iodine­(VII) were so far unsuccessful. The reaction of IF_7_ with B­(OTeF_5_)_3_ resulted in the formation of [IF_6_]​[BF­(OTeF_5_)_3_], with the cation [IF_6_]^+^ being identified by Raman spectroscopy.^21^ Group 17 teflates and selenates are gathered in Table 8.

## 10 Group 18 Teflates

For the lighter noble gases (Ng) helium, neon and argon, no teflate compounds are known to date.

### 10.1 Krypton Teflates

In 1979, Seppelt et al. attempted to synthesize the first krypton teflate. However, when KrF_2_ reacted with B­(OTeF_5_)_3_ in ClO_3_F at −100 °C, the formation of krypton and F_5_TeOOTeF_5_ was observed instead.^16^ Another attempt using HOTeF_5_ in ClO_3_F together with KrF_2_ at −30 °C also failed to yield Kr­(OTeF_5_)_2_.^217^

A decade later, Schrobilgen et al. successfully synthesized Kr­(OTeF_5_)_2_ by reacting KrF_2_ with B­(OTeF_5_)_3_ in SO_2_ClF at −110 °C (see eq 41). This marked the first Kr–O bond reported at this time. The compound was characterized by a distinctive AB_4_ pattern in the ^19^F and ^17^O NMR spectra, which diminished as temperature increased to −90 °C, decomposing into krypton and F_5_TeOOTeF_5_. Chemical shift comparisons indicated a more ionic nature for the teflate group in Kr­(OTeF_5_)_2_ compared to analogous compounds like FXeOTeF_5_ and Xe­(OTeF_5_)_2_.^201^


42 3KrF 2+2B(OTeF 5)3→3Kr(OTeF 5)2+2BF 3

### 10.2 Xenon­(II) Teflates

The first noble gas species involving a teflate group were initially reported by Sladky et al. in 1969, including FXeOTeF_5_, Xe­(OTeF_5_)_2_ and [XeOTeF_5_]​[AsF_6_].^218–220^ The monoteflate substituted neutral xenon­(II) compound FXeOTeF_5_ appears as a pale yellow liquid under normal conditions and is produced by HF displacement in the reaction of XeF_2_ with HOTeF_5_ (see eq 42).^218^ Additionally, FXeOTeF_5_ can be obtained in quantitative yields by the equimolar reaction of XeF_2_ with Xe­(OTeF_5_)_2_, as shown in eq 43.^13^ The fully teflate-substituted neutral xenon­(II) compound, Xe­(OTeF_5_)_2_ is synthesized by adding a second equivalent of HOTeF_5_ to the FXeOTeF_5_, which is formed *in situ*.^219^ The presence of HF and HOTeF_5_ forms an equilibrium involving XeF_2_, FXeOTeF_5_ and Xe­(OTeF_5_)_2_, necessitating the freeze–pump–thaw method to eliminate generated HF from the reaction mixture. This process is repeated until solid Xe­(OTeF_5_)_2_ remains upon further addition of HOTeF_5_, indicating that the liquid FXeOTeF_5_ does no longer exist in the reaction mixture.^221^


43 XeF 2⇌HFHOTeF 5FXeOTeF 5⇌HFHOTeF 5Xe(OTeF 5)2

44 XeF 2+Xe(OTeF 5)2→2FXeOTeF 5

An alternative approach to synthesizing Xe­(OTeF_5_)_2_ involves the reagents XeF_2_ and B­(OTeF_5_)_3_ in CFCl_3_, resulting in immediate precipitation of Xe­(OTeF_5_)_2_ free from FXeOTeF_5_ (see eq 44).^1,44^ This approach eliminates the formation and, consequently, the quenching of HF. Xe­(OTeF_5_)_2_ appears as colorless crystals (see Figure 18). In the molecular structure of Xe­(OTeF_5_)_2_ the linear arrangement of O–Xe–O with the measured angle of 180° is consistent with the AX_2_E_3_ VSEPR arrangement typical for xenon­(II) compounds.^12,106^


45 3XeF 2+2B(OTeF 5)2→3Xe(OTeF 5)2+2BF 3

**Figure 18:**
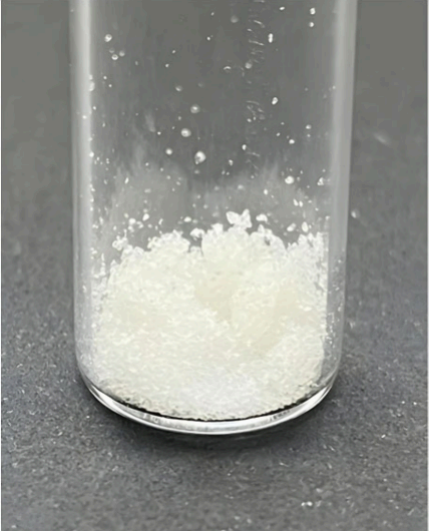
Crystals of Xe­(OTeF_5_)_2_, obtained by the reaction of XeF_2_ with excess HOTeF_5_.

An equimolar reaction of Xe­(OTeF_5_)_2_ with Xe­(OSeF_5_)_2_ in CFCl_3_ results in the formation of the mixed compound, Xe­(OTeF_5_)​(OSeF_5_), as detected by ^19^F NMR spectroscopy. However, these compounds exist only in an equilibrium.^5,107^ Another mixed-ligand derivative, Xe­(OTeF_5_)​(OIOF_4_), is spectroscopically identified during the synthesis of Xe­(OIOF_4_)_2_. This was done by reacting Xe­(OTeF_5_)_2_ with the protic acid HOIOF_4_ in CFCl_3_.^222^

Moreover, formation of a polymeric xenon­(II) species was reported by Seppelt et al. in the reaction of *cis*-(HO)_2_TeF_4_ with XeF_2_ in C_4_F_9_SO_2_F at room temperature.^223^ The compound is formed by HF elimination and exists as a pale yellow solid with the composition (XeO_2_TeF_4_)_*n*_. This polymeric compound is insoluble in all common solvents and decomposes by release of xenon and oxygen at temperatures above 80 °C. In contrast, likely due to its lower acidity compared to the *cis*-isomer, *trans*-(HO)_2_TeF_4_ does not react under the same conditions.^224^

In a follow-up study, reacting *cis*-(HO)_2_TeF_4_ with [Xe_2_F_3_]​[AsF_6_] produced a stable yellow solid, [Xe_2_FO_2_TeF_4_]​[AsF_6_]. This compound is insoluble in chlorofluorocarbons and SO_2_ClF. It decomposes at −70 °C in SO_2_ and at −40 °C in MeCN, yet dissolves readily in HF. When [Xe_2_FO_2_TeF_4_]​[AsF_6_] is kept in HF for extended periods of time at low temperatures, orange crystals of the solvolysis product [HF·HOTeF_4_OXe]​[AsF_6_] form. The molecular structure of this product shows bridging cation–anion interaction between xenon and one fluorine atom of the [AsF_6_]^−^ anion.^224^

The xenon­(II) teflate cation [Xe­(OTeF_5_)]^+^ was initially reported by Sladky et al. as the product of the reaction of FXe­(OTeF_5_) with AsF_5_ (see Scheme 10).^220^ The resulting salt [Xe­(OTeF_5_)]​[AsF_6_] exhibits significant cation–anion interaction through a fluoride bridge, as shown by Raman spectroscopy.^225^ This interaction was further confirmed by the molecular structure of the salt, which shows a short Xe–F contact distance of 224(3) pm.^106^

**Scheme 10:**
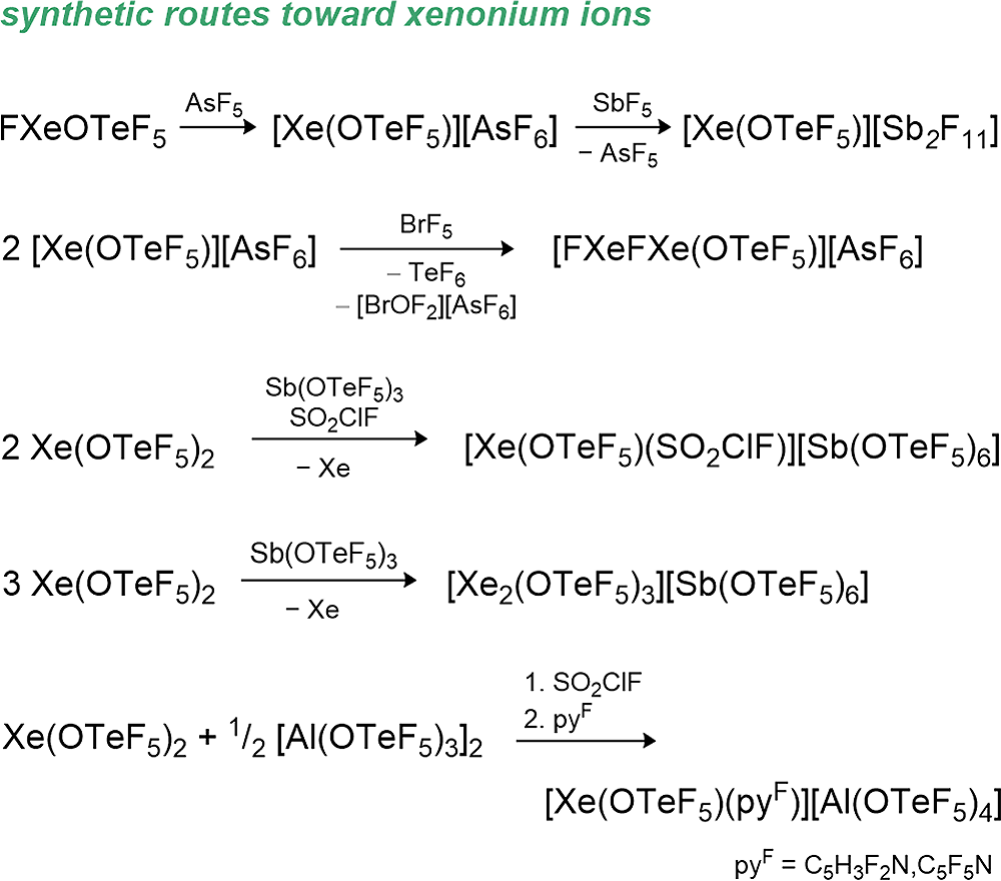
Overview of Teflate-Based Xenonium Ions and Their Synthesis

When [Xe­(OTeF_5_)]​[AsF_6_] is dissolved in liquid SbF_5_, [Xe­(OTeF_5_)]​[Sb_2_F_11_] is formed as a yellow-orange solution due to displacement of AsF_5_ (see Scheme 10). While a fluoride-bridge interaction similar to that in [Xe­(OTeF_5_)]​[AsF_6_] is expected for this salt, the Raman spectrum obtained was too complex to assign specific bands.^225^

In contrast, when [Xe­(OTeF_5_)]​[AsF_6_] is dissolved in BrF_5_ at −48 °C, it forms the cation [FXeFXeOTeF_5_]^+^, characterized by the presence of a teflate group in the ^19^F NMR spectrum and two distinct chemical shifts in the ^129^Xe NMR spectrum (see Scheme 10).^225^

The first fully teflate-substituted salt, [Xe­(OTeF_5_)]​[Sb­(OTeF_5_)_6_], was synthesized during the investigation of Sb­(OTeF_5_)_5_ by oxidatively transferring two teflate groups from Xe­(OTeF_5_)_2_ to Sb­(OTeF_5_)_3_. However, instead of the pentavalent antimony teflate compound, the product of the reaction was the noble gas cation [Xe­(OTeF_5_)]^+^ countered by the weakly coordinating anion [Sb­(OTeF_5_)_6_]^−^ (see Scheme 10).^43^ The salt was later characterized in the solid state, showing that it forms an adduct with the weakly basic SO_2_ClF solvent molecule, rather than being a monocoordinated xenonium center.^43,69^ The strong two-electron oxidizing property of the salt [Xe­(OTeF_5_)​(SO_2_ClF)]​[Sb­(OTeF_5_)_6_] makes it a useful reagent in the preparation of trihalomethyl carbocations.^67^ Additionally, the reaction of 3 equiv of Xe­(OTeF_5_)_2_ with Sb­(OTeF_5_)_3_ in SO_2_ClF generates the [Xe_2_(OTeF_5_)_3_]^+^ cation, as confirmed by Raman spectroscopy of the resulting solid (see Scheme 10). The calculated molecular structure of [Xe_2_(OTeF_5_)_3_]^+^ suggests *C*_2*v*_ symmetry for the free cation, similar to that of the known fluoride analogue [Xe_2_F_3_]^+^.^69^

Considering that the Lewis acidity of the [Xe­(OTeF_5_)]^+^ cation is similar to that of the well-known [XeF]^+^ cation, it is noted, without much detail given, that [Xe­(OTeF_5_)]^+^ forms cationic adducts with nitrogen bases. These adducts include [Xe­(OTeF_5_)​(MeCN)]^+^, [Xe­(OTeF_5_)​(C_5_F_5_N)]^+^ and [Xe­(OTeF_5_)​(C_3_F_3_N_3_)]^+^ (C_3_F_3_N_3_ = cyanuric fluoride), existing as salts of the [Sb­(OTeF_5_)_6_]^−^ anion. Additionally, a selenium derivative, [Xe­(OSeF_5_)​(NSF_3_)]​[AsF_6_], has also been reported.^226^

Recently, Riedel et al. reported that the Lewis superacid Al­(OTeF_5_)_3_ is able to abstract a teflate group from Xe­(OTeF_5_)_2_, resulting in a nonisolable intermediate salt, [Xe­(OTeF_5_)]​[Al­(OTeF_5_)_4_], which forms a yellow solution in SO_2_ClF. This intermediate shows a complex ^19^F NMR spectrum, indicating rapid exchange of teflate groups between the cation and the anion. Upon adding a fluorinated nitrogen base, cationic adducts [Xe­(OTeF_5_)​(py^F^)]^+^ (py^F^ = C_5_F_5_N, C_5_H_2_F_3_N) will be formed, countered by the weakly coordinating [Al­(OTeF_5_)_4_]^−^ anion (see Scheme 10). These compounds were characterized using low-temperature spectroscopic methods like ^19^F NMR, ^129^Xe NMR, IR and Raman spectroscopy. Also, the high oxidation potential of [Xe­(OTeF_5_)​(C_5_F_5_N)]​[Al­(OTeF_5_)_4_] has been experimentally demonstrated by the oxidation of tris­(4-tribromophenyl)­amine, a derivative of “magic blue”, to form the ammoniumyl radical cation.^70^

Furthermore, changing the anion with [Sb­(OTeF_5_)_6_]^−^ facilitated the growth of single crystals for the adduct salt [Xe­(OTeF_5_)​(C_5_F_5_N)]​[Sb­(OTeF_5_)_6_].^70^ This compound represents the second example of the heteroleptic xenonium­(II) teflate cation in the solid state, alongside the previously reported [Xe­(OTeF_5_)​(SO_2_ClF)]​[Sb­(OTeF_5_)_6_]. Both structures confirm a nearly linear alignment at the xenonium­(II) center (see Figure 19).

**Figure 19:**
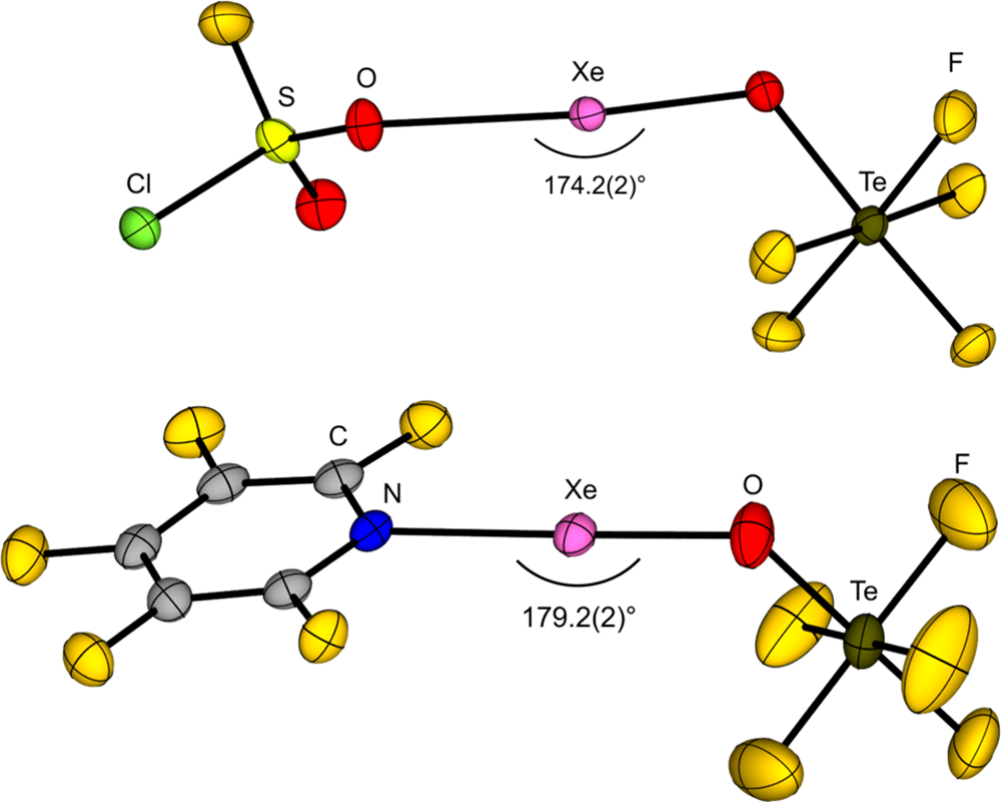
Molecular structures in the solid state of the [Xe­(OTeF_5_)​(SO_2_ClF)]^+^ cation (top) and the [Xe­(OTeF_5_)​(C_5_F_5_N)]^+^ cation (bottom).^43,70^ The [Sb­(OTeF_5_)_6_]^−^ counteranions are omitted for clarity.

The selenate derivatives of xenon­(II) species are FXeOSeF_5_,^106,107^ Xe­(OSeF_5_)_2_,^5,106,107,227^ and the cation [Xe­(OSeF_5_)]^+^ as its [AsF_6_]^−^ salt.^106,226^

### 10.3 Xenon­(IV) Teflates

The chemistry of xenon­(IV) teflate compounds began with the preparation of Xe­(OTeF_5_)_4_ by reacting XeF_4_ with B­(OTeF_5_)_3_ in perfluoro-*n*-hexane at 0 °C, as shown in eq 45.^228^


46 XeF 4+43B(OTeF 5)3→Xe(OTeF 5)4+43BF 3

The resulting yellow crystalline compound can be sublimed under vacuum at 50 °C but is found to be less thermally stable compared to its fluorine analogue XeF_4_. This difference is attributed to the formation of a relatively strong peroxide bond in F_5_TeOOTeF_5_, which is the decomposition product of Xe­(OTeF_5_)_4_, unlike elemental fluorine in the case of XeF_4_. Moreover, Xe­(OTeF_5_)_4_ decomposes slowly under ambient conditions and even at low temperatures in solution.^217^ In its solid state, the molecular structure of Xe­(OTeF_5_)_4_ exhibits a planar XeO_4_ geometry (see Figure 20).^229^

**Figure 20:**
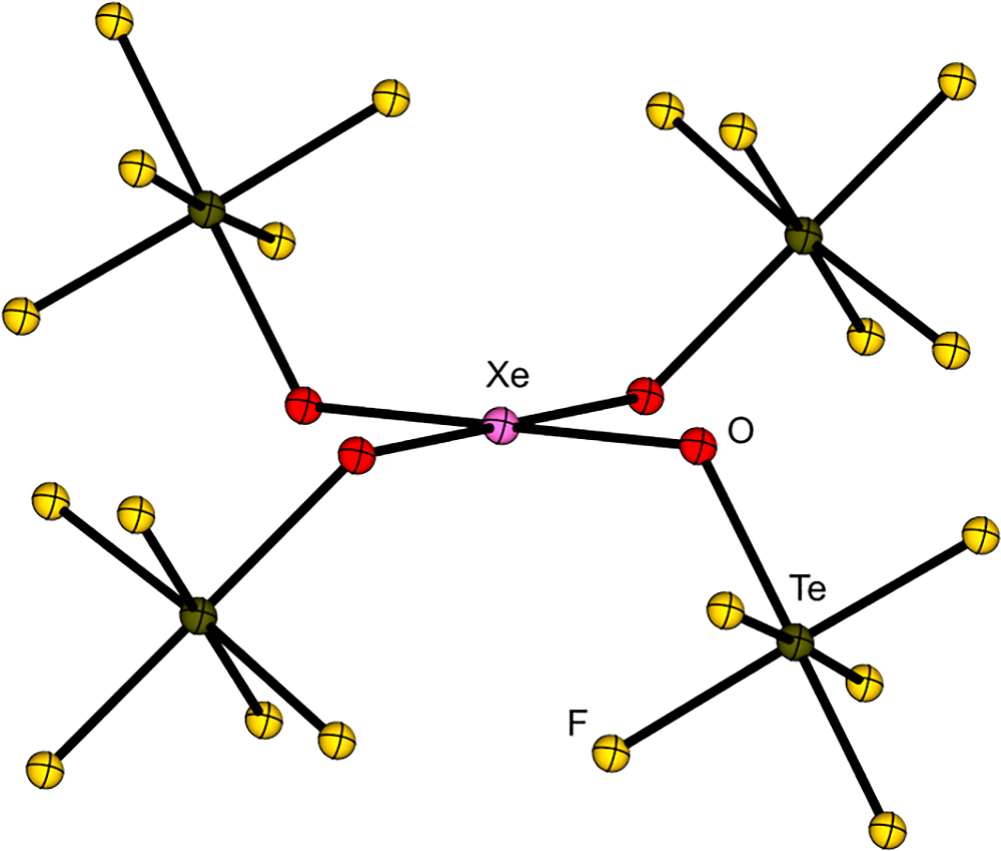
Molecular structure of Xe­(OTeF_5_)_4_ in the solid state.^229^

A series of mixed-ligand species XeF_*n*_(OTeF_5_)_4–*n*_ (*n* = 0–4) are produced by mixing Xe­(OTeF_5_)_4_ with the fluorine analogue XeF_4_ in CFCl_3_, as shown in eq 46. These compounds were identified using ^19^F NMR and ^129^Xe NMR spectroscopy. The findings indicate that the teflate group has a lower effective electronegativity compared to fluorine, as revealed by difference in the ^129^Xe NMR chemical shifts.^230^


47 Xe(OTeF 5)4+XeF 4→2XeFn(OTeF 5)4−nn=0−4

Additionally, when Xe­(OTeF_5_)_4_ is dissolved in SbF_5_, it forms the cationic mixed-ligand species [XeF_*n*_(OTeF_5_)_3–*n*_]^+^ (*n* = 0–2) (see eq 47). The process likely begins with SbF_5_ abstracting one teflate group, generating [Xe­(OTeF_5_)_3_]^+^, followed by a rearrangement of fluorine and teflate ligands. While mixed-ligand cations can be observed at 5 °C, the solutions gradually decompose, indicated by the gas evolution.


48 Xe(OTeF 5)4→SbF 5[XeFn(OTeF 5)3−n][SbF 5−n(OTeF 5)1+n]n=0−2

**Table 9:** Overview of Group 18 Teflate and Selenate Compounds

Element	Ox	Compound	Refs
Kr	II	Kr(OTeF_5_)_2_	^16,201^

Xe	II	FXe(OTeF_5_)	^13,107,218^
		Xe(OTeF_5_)_2_	^1,12,32,106,107,219,221^
		[Xe(OTeF_5_)]​[AsF_6_]	^14,106,218,220,225^
		[Xe(OTeF_5_)]​[Sb_2_F_11_]	^225^
		[Xe(OTeF_5_)]​[Sb(OTeF_5_)_6_]	^67,226,232^
		[Xe(OTeF_5_)​(SO_2_ClF)]​[Sb(OTeF_5_)_6_]	^43,69,226^
		[Xe(OTeF_5_)​(MeCN)]​[Sb(OTeF_5_)_6_]	^226^
		[Xe(OTeF_5_)​(C_3_F_3_N_3_)]​[Sb(OTeF_5_)_6_]	^226^
		[Xe(OTeF_5_)​(C_5_F_5_N)]​[Sb(OTeF_5_)_6_]	^70,226^
		[Xe(OTeF_5_)​(C_5_F_5_N)]​[Al(OTeF_5_)_4_]	^70^
		[Xe(OTeF_5_)​(C_5_H_2_F_3_N)]​[Al(OTeF_5_)_4_]	^70^
		Xe(OTeF_5_)​(OSeF_5_)	^5^
		Xe(OTeF_5_)​(OIOF_4_)	^222^
		(XeO_2_TeF_4_)_*n*_	^223,224^
		[Xe_2_(OTeF_5_)_3_]​[Sb(OTeF_5_)_6_]	^69^
		[FXeFXe(OTeF_5_)]​[AsF_6_]	^225^
		[FXeOTeF_4_OXe]​[AsF_6_]	^224^
	IV	Xe(OTeF_5_)_4_	^32,217,228,229^
		XeF_*n*_(OTeF_5_)_4–*n*_ (*n* = 0–4)	^230^
		[XeF_*n*_(OTeF_5_)_3–*n*_]^+^ (*n* = 0–2)^a^	^231^
	VI	Xe(OTeF_5_)_6_	^16,217^
		OXe(OTeF_5_)_4_	^16,32,217,230^
		OXeF_*n*_(OTeF_5_)_4–*n*_ (*n* = 0–4)	^217,230^
		[OXeF_*x*_(OTeF_5_)_3–*x*_]​[Sb_*n*_F_(5*n*+1)–*m*_(OTeF_5_)_*m*_] (*x* = 0–3, *m* = 0–5, *n* = 1, 2, 3...)	^231^
		O_2_Xe(OTeF_5_)_2_	^229,230^
		O_2_XeF_*n*_(OTeF_5_)_2–*n*_ (*n* = 0–2)	^230^
		[O_2_XeF_*n*_(OTeF_5_)_1–*n*_]^+^ (*n* = 0–1)^a^	^231^
	II	Xe(OSeF_5_)_2_	^87,103–107^
		FXe(OSeF_5_)	^103,106,107^
		[Xe(OSeF_5_)]​[AsF_6_]	^106^

a Cation generated in SbF_5_ solvent; corresponding anion not specified in the reference.

Although the [Xe­(OTeF_5_)_3_]^+^ cation can be observed in SbF_5_, attempts to synthesize [Xe­(OTeF_5_)_3_]​[Sb­(OTeF_5_)_6_] by reacting Xe­(OTeF_5_)_4_ with “Sb­(OTeF_5_)_5_” (formed *in situ* from Xe­(OTeF_5_)_2_ with Sb­(OTeF_5_)_3_ in SO_2_ClF) have been unsuccessful so far.^231^

### 10.4 Xenon­(VI) Teflates

The highest known oxidation state observed for Ng-teflate compounds is xenon­(VI). The homoleptic compound Xe­(OTeF_5_)_6_ was synthesized by reacting XeF_6_ with B­(OTeF_5_)_3_ in perfluoro-*n*-pentane at −40 °C (see eq 48).^16^ This deep red crystalline compound is highly sensitive to temperature, air as well as light and is either insoluble or decomposes in all common solvents. Due to its sensitivity, spectroscopic investigations are very limited. Nevertheless, it could be analyzed through its decomposition products Xe­(OTeF_5_)_4_ and the peroxide F_5_TeOOTeF_5_. Decomposition occurs thermally at −10 °C or when exposed to daylight, even at −230 °C. Its direct UV light decomposition results in the formation of elemental xenon and F_5_TeOOTeF_5_. At room temperature, Xe­(OTeF_5_)_6_ decomposes into Xe­(OTeF_5_)_2_ and F_5_TeOOTeF_5_ within 3 days.^217^


49 XeF 6+2B(OTeF 5)3→Xe(OTeF 5)6+2BF 3

Compared to XeF_6_, the shielding effect of the teflate group provides additional stability to Xe­(OTeF_5_)_6_ against hydrolysis. When hydrolyzed, it forms the colorless compound OXe­(OTeF_5_)_4_. This oxo compound can be synthesized directly by reacting XeOF_4_ with B­(OTeF_5_)_3_ in fluoro-*n*-pentane at −30 °C (see eq 49).^16^


50 XeOF 4→−43BF 343B(OTeF 5)3OXe(OTeF 5)4

When OXe­(OTeF_5_)_4_ and XeOF_4_ are dissolved in F-114 (1,2-dichloro-1,1,2,2-tetrafluoroethane), they undergo ligand scrambling, leading to the formation of various mixed-ligand species labeled as OXeF_*n*_(OTeF_5_)_4–*n*_ (*n* = 0–4), as shown in eq 50. The existence of *cis*- and *trans*-isomers of OXeF_2_(OTeF_5_)_2_ suggests that these species adopt a square pyramidal geometry with the oxygen atom positioned at the apex relative to the center of the base.^217^


51 XeOF 4+OXe(OTeF 5)4→2OXeFn(OTeF 5)4−nn=0−4

Similar to [XeF_*n*_(OTeF_5_)_3–*n*_]^+^, when OXe­(OTeF_5_)_4_ is dissolved in SbF_5_ it forms cationic mixed-ligand oxo compounds [OXeF_*n*_(OTeF_5_)_3–*n*_]^+^ (*n* = 0–2) (see eq 51). This occurs because SbF_5_ abstracts one teflate group, leading to ligand scrambling in the solution.^231^


52 OXe(OTeF 5)4→SbF 5[OXeFx(OTeF 5)3−x][SbnF(5n+1)−m(OTeF 5)m]x=0−3,m=0−5,n=1,2,3...

To prepare dioxo-xenon­(IV) teflate species, XeO_2_F_2_ can be reacted with B­(OTeF_5_)_3_ in SO_2_ClF. When equimolar amounts are used, O_2_Xe­(OTeF_5_)_2_ can be quantitatively obtained. Using an excess of XeO_2_F_2_ produces mixed-ligand compounds like O_2_XeF_*n*_(OTeF_5_)_2–*n*_ (*n* = 0–2). According to ^129^Xe NMR spectroscopic studies, these species exhibit a trigonal bipyramidal molecular geometry with oxygen atoms occupying the equatorial positions around the xenon­(VI) center.^230^ Attempts to crystallize OXe­(OTeF_5_)_4_ have resulted in O_2_Xe­(OTeF_5_)_2_ due to partial decomposition. O_2_Xe­(OTeF_5_)_2_ is stable at room temperature and exhibits a pseudo trigonal bipyramidal or distorted tetragonal geometry in the solid state, influenced by strong steric hindrance from the lone pair, as shown in Figure 21.^229^

**Figure 21:**
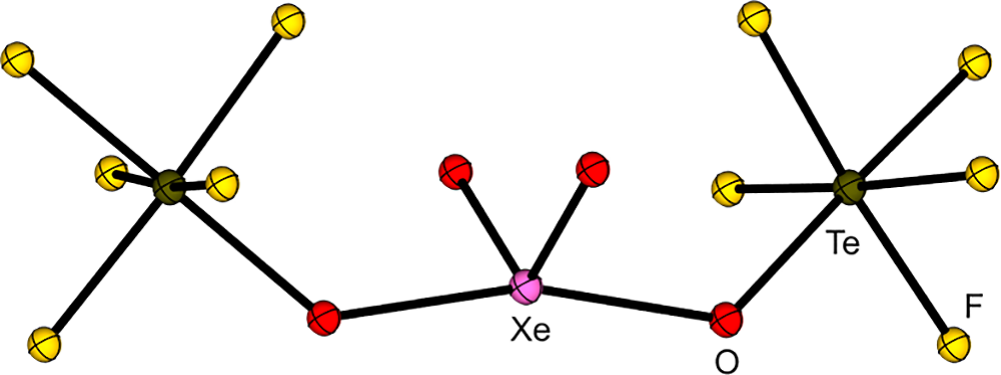
Molecular structure of O_2_Xe­(OTeF_5_)_2_ in the solid state.^229^

Additionally, when OXe­(OTeF_5_)_4_ is dissolved in SbF_5_, it forms cationic xenon­(VI) species [O_2_XeF_*n*_(OTeF_5_)_1–*n*_]^+^ (*n* = 0–1) as decomposition products. Although these cations have been detected via NMR spectroscopy, further decomposition yields O_2_, TeF_6_, F_5_TeOOTeF_5_ and the cationic species [XeF]^+^ and [XeOTeF_5_]^+^.^231^ Group 17 teflates and selenates are gathered in Table 9.

## 11 *d*-Block and *f*-Block Metal Teflates

Since the synthesis of Hg­(OTeF_5_)_2_ in the early 1970s,^5^ the number of compounds containing teflate ligands coordinated to *d*-block metals has increased to find representatives of most of these elements. The main progress took place in the decades of the 1980s and 1990s. However, it has only been recently, that the properties of the teflate group as a ligand in coordination chemistry have been investigated, in order to ascertain its similarity in terms of electronic properties to the fluoride ligand.^34,36^ In comparison, the related ligands OSeF_5_ and OSF_5_ are far less studied. In fact, whereas compounds containing the OSeF_5_ ligand are known but are far more scarce than teflate-based ones, the first OSF_5_ *d*-metal complexes were only reported recently.^110^

To facilitate the analysis of such a diverse class of compounds, they will appear divided in homoleptic and heteroleptic complexes. Homoleptic complexes often entail a synthetic challenge, whereas at the same time they are extremely interesting for the investigation of fundamental properties associated with metal–ligand pairs. In the particular case of the teflate ligand, this has not been an exception and homoleptic metal teflate species constitute a group of compounds of high importance by itself, gathering species of many different characteristics and applications. Herein, we will discuss first the neutral species and afterward the anionic ones. In the case of heteroleptic compounds, it will be shown how teflate is compatible with many different ligands, allowing for coordination spheres with a large number of possibilities.

Regarding *f*-block metal teflates, uranium provides the only example and will be described in a separate section.

### 11.1 Homoleptic *d*-Block Metal Teflates

#### 11.1.1 Neutral Complexes

Neutral metal teflate complexes are known for a handful of elements in various oxidation states. They showcase interesting features, ranging from the suitability of some as teflate transfer reagents, to the acidic properties of others. The most popular *d*-block metal teflate species are Hg­(OTeF_5_)_2_ and AgOTeF_5_ due to their widespread use as teflate transfer reagents, as becomes clear along this review. The Hg­(II) species Hg­(OTeF_5_)_2_ was first reported in 1973 by the group of Seppelt to be prepared by reaction of HgF_2_ and HOTeF_5_ in a Teflon vessel,^5^ leading to a white residue that had to be purified via sublimation at 180 °C^5^ or 200 °C.^11^ An improved synthetic route by Schrobilgen et al. appeared in 2014 to produce Hg­(OTeF_5_)_2_ in high yield and purity without the need for further purification. This approach uses high-purity HgF_2_, which is reacted with a slight excess of HOTeF_5_ at 50 °C.^233^ Based on this highly pure compound, a more thorough characterization by ^19^F NMR and Raman spectroscopy was possible, as well as in the solid state by single crystal X-ray diffraction. The structure of Hg­(OTeF_5_)_2_ consists of oxygen-bridged chains, as opposed to the three-dimensional polymeric structure of HgF_2_. A distorted octahedral coordination sphere at the Hg­(II) center results, with the Hg–O bonds *trans* to each other and the Hg–O and Hg–F contacts *cis* to one another and to the Hg–O bonds. Computationally, both a *gauche*-conformation (*C*_2*h*_ symmetry),^234^ as well as an *anti*-conformation (*C*_2_ symmetry)^233^ have been optimized. Nevertheless, in the optimized *gauche* one, related to that of the crystal structure, there is no intermolecular interaction with other units, leading to a 4-fold coordination with two primary Hg–O bonds and two weak interactions from equatorial F atoms of the teflate ligands.^234^ The calculated gas-phase structure of the unknown trimeric [Hg­(OTeF_5_)_2_]_3_ species retains the *gauche* conformation of the solid state.^233^

The related compound with OSeF_5_ ligands, Hg­(OSeF_5_)_2_, was reported even earlier, in 1972.^88^ A similar synthetic route was used in this case starting from HgF_2_ and an excess HOSeF_5_. The compound was obtained after sublimation at 180 °C as colorless and hydrolysis-sensitive crystals, but still, has been subsequently used for follow-up chemistry.^42,86,87,90^

The Ag­(I) species AgOTeF_5_ was first reported in 1975 by Mayer and Sladky as a white crystalline solid, obtained by reaction of HOTeF_5_ and AgCN in MeCN.^10^ The contact ion-pair formation in the AgOTeF_5_/MeCN system was proved by IR and Raman spectroscopy,^10^ based on which, the compound was better formulated as [Ag­(MeCN)_2_]­OTeF_5_.^11^ Later, it was suggested to have a dimeric structure with bridging teflate ligands.^165^ Similar reactions with AgCN using CH_2_Cl_2_ as a solvent or neat HOTeF_5_ (50 °C) do not take place.^165^ Nevertheless, AgF and HOTeF_5_ react in DCM at room temperature to yield [Ag­(DCM)​(OTeF_5_)]_2_, which can be transformed into AgOTeF_5_ under 4 h of dynamic vacuum,^165,235^ providing a gram-scale synthesis for this compound. The coordination of DCM is reversible, with solid AgOTeF_5_ readily absorbing dichloromethane vapor.^235^ Recrystallization of [Ag­(OTeF_5_)​(DCM)]_2_ in toluene yields [Ag­(OTeF_5_)​(tol)_2_]_2_ (tol = toluene), which exhibits a dimeric structure in the solid state with bridging OTeF_5_ ligands leading to a {Ag_2_O_2_} core.^165^ Two η^2^-coordinated toluene molecules to each silver center result in a pseudo tetrahedral coordination environment at each Ag­(I) center. Additionally, AgOTeF_5_ forms [Ag­(OTeF_5_)​(DCE)]_2_ in the presence of 1,2-dichloroethane. This dimeric compound shows again a {Ag_2_O_2_} core, in which each Ag center is additionally coordinated by one bidentate 1,2-dichloroethane molecule and stabilized by weak contacts with F and Cl atoms from neighboring dimers.^235,236^ A rather unusual silver complex can be observed by dissolving AgOTeF_5_ in 1,2,3-trichloropropane, [Ag­(OTeF_5_)​(1,2,3-C_3_H_5_Cl_3_)]_2_. Here, a chain-like structure is formed by the linkage of Ag_2_(OTeF_5_)_2_ units by 1,2,3-trichloropropane via Ag–Cl bonds.^237^

In the case of silver, the related OSeF_5_ species is not known, but AgOSF_5_ was recently reported, although it was already proposed in the 1960s as an intermediate in fluorination processes.^238^ Compound AgOSF_5_ is synthesized by reaction of AgF with SOF_4_ in MeCN (see eq 52), yet the compound consists in fact of solvated tetrahedral [Ag­(MeCN)_4_]^+^ units.^110^ This explains why acetonitrile solutions of this species are stable at room temperature in standard glassware, yet evaporation of the solvent leads to decomposition. The compound is stable against light and its solutions can be used to transfer the OSF_5_ unit. This way, for instance, the reaction with CuI leads to the related CuOSF_5_ complex (eq 52), that crystallized as [Cu­(MeCN)_4_]​[OSF_5_]. Interestingly, to the best of our knowledge, no Cu teflate species has been reported up to now.


53 AgF+SOF 4→AgOSF 5(solv)→−AgICuICuOSF 5(solv)

Another neutral metal teflate complex of great significance is Au­(OTeF_5_)_3_, as it triggered the first systematic study of the Lewis acidity of a teflate-containing transition metal-based Lewis acid.^24^ The compound is actually dimeric in the solid state, with both Au­(III) centers in a near square planar environment, being the first ever reported teflate-based compound containing bridging teflate ligands (see Figure 22).^40^ As expected, the Au–O_bridge_ distances are longer (223(4) pm and 229(4) pm) than the Au–O_terminal_ bond lengths (178(4) pm and 182(3) pm).

**Figure 22:**
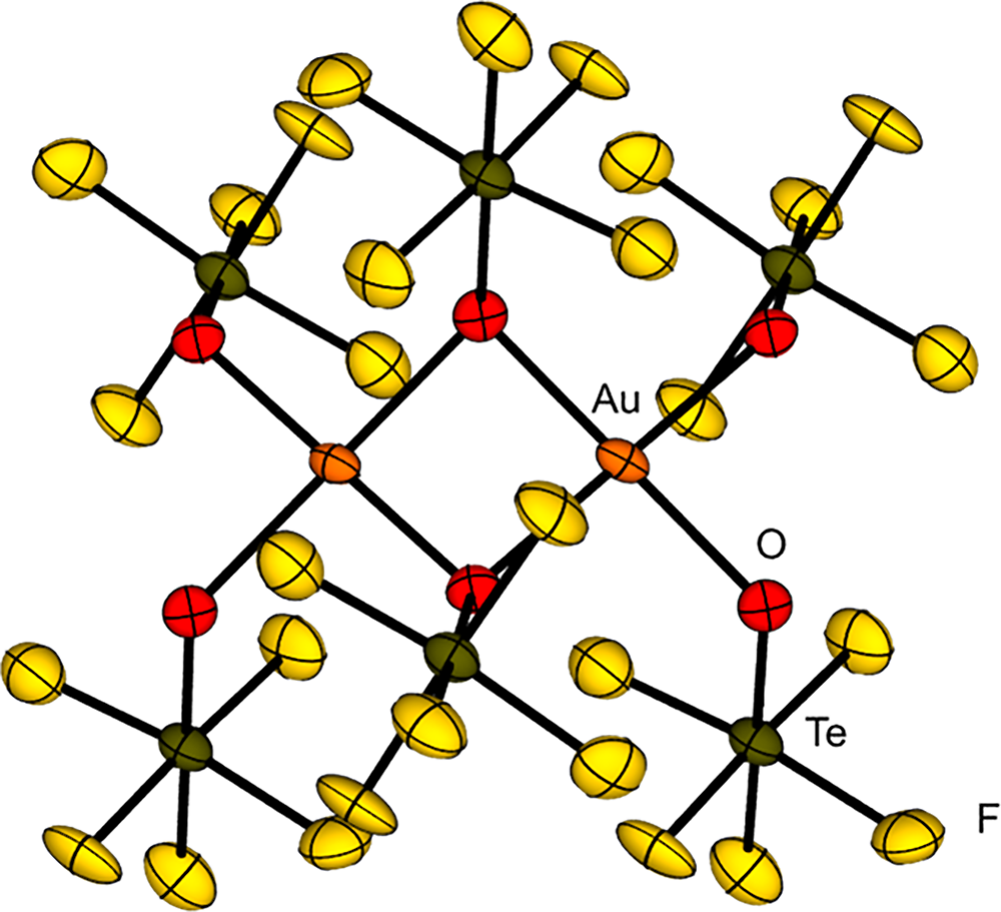
Molecular structure in the solid state of Au­(OTeF_5_)_3_, showing its dimeric nature.^40^ Thermal ellipsoids are set at 30% probability.

The compound Au­(OTeF_5_)_3_, which has been known since 1985,^40^ was first prepared by the group of Seppelt by reaction of AuF_3_ and B­(OTeF_5_)_3_ at 60 °C during 5 days (see eq 53). A newer synthetic method by Riedel et al. relies on the use of commercial AuCl_3_ and neat ClOTeF_5_ (see eq 53a).^24^ Compound Au­(OTeF_5_)_3_ could be classified as a Lewis superacid according to its FIA value (557 kJ mol^–1^ for the monomer, 504 kJ mol^–1^ for the dimer), the Gutmann–Beckett method and the analysis of the C≡N vibration in the CD_3_CN adduct.^24^ Additionally, Au­(OTeF_5_)_3_ is able to abstract a fluoride from [PPh_4_]​[SbF_6_] in SO_2_ClF, yielding a complex mixture arising from the initial abstraction of the fluoride, formation of SbF_5_ and further ligand scrambling. All this underlines the use of the teflate ligand in the synthesis of new Lewis superacids, since the related AuF_3_ does not reach the acidity to belong to this category. Furthermore, Au­(OTeF_5_)_3_ is the only gold-based Lewis superacid after AuF_5_.[Bibr newref242]


54 AuF 3+B(OTeF 5)3→−BF 3Au(OTeF 5)3

55 AuCl3+3ClOTeF 5→−3Cl2Au(OTeF 5)3

The only known neutral homoleptic group 4 complex is Ti­(OTeF_5_)_4_, which was first mentioned in the literature in 1975 as the product of the reaction of TiCl_4_ and Hg­(OTeF_5_)_2_ (see eq 54).^42^ It was described as a crystalline solid and a monomeric structure was assumed according to its high volatility despite its high molecular mass and its solubility in nonpolar solvents. The equimolar reaction of TiCl_4_ and HOTeF_5_ affords TiCl_3_(OTeF_5_), whose dismutation eventually renders also Ti­(OTeF_5_)_4_ (see eq 54a).^239^ The intermediate species TiCl_2_(OTeF_5_)_2_ and TiCl­(OTeF_5_)_3_ were also detected by NMR spectroscopy. Compound Ti­(OTeF_5_)_4_ exhibits Lewis acidic behavior, forming 1:1 and 1:2 adducts with POCl_3_.^239^


56 TiCl4→−2HgCl22Hg(OTeF 5)2Ti(OTeF 5)4

57 TiCl4→−HClHOTeF 5TiCl3(OTeF 5)→Ti(OTeF 5)4

Regarding group 5, the neutral compounds Nb­(OTeF_5_)_5_ and Ta­(OTeF_5_)_5_ were prepared by reaction of MCl_5_ (M = Nb, Ta) with an excess of HOTeF_5_ (see eq 55).^65^ The reaction of the fluorides NbF_5_ and TaF_5_ with B­(OTeF_5_)_3_ was reported to lead to the same five-coordinate species (see eq 55a) and not to oxo derivatives, which is often the case in such processes. These compounds were reported to have a limited solubility, to be nonvolatile, as well as to decompose into viscous oils.^142^


58 MCl5+5HOTeF 5→−5HClM(OTeF 5)5M=Nb,Ta

59 MF 5+53B(OTeF 5)3→−53BF3M(OTeF 5)5M=Nb,Ta

The teflate compounds of the group 6 elements Mo and W are known for oxidation state +VI (Mo­(OTeF_5_)_6_ and W­(OTeF_5_)_6_), but only in the case of tungsten the compound W­(OTeF_5_)_5_ in oxidation state +V was realized. W­(OTeF_5_)_5_ was reported to be prepared as a yellow solid by reaction of WCl_5_ and ClOTeF_5_ in CFCl_3_ between −30 °C and room temperature (see eq 56).^240^ Unfortunately, no single crystal XRD analysis of the molecular structure of this compound was possible, contrary to WO­(OTeF_5_)_4_, which arises from the decomposition of W­(OTeF_5_)_5_ already at −78 °C (see Section 11.2.1). Highly moisture-sensitive W­(OTeF_5_)_6_ was reported to be isolated as a colorless solid by reaction of WCl_5_ and Xe­(OTeF_5_)_2_ in CFCl_3_ (see eq 57)^240^ better than between WF_5_ and B­(OTeF_5_)_3_ in 1,1,2-trichlorotrifluoroethane (F-113), from which a disproportionation process of the pentavalent tungsten intermediate should happen. The corresponding reaction starting from WF_6_ and B­(OTeF_5_)_3_ only led to partial substitution, similarly to the attempts performed from WCl_6_ and Hg­(OTeF_5_)_2_ or ClOTeF_5_.^142^ The Mo­(VI) analogue, Mo­(OTeF_5_)_6_, is prepared by means of the hypochlorite ClOTeF_5_ but starting from MoCl_5_ (see eq 58). This means that, in contrast to the synthesis of W­(OTeF_5_)_5_, in this case the reaction takes place with oxidation of the metal center.


60 WCl5+5ClOTeF 5→W(OTeF 5)5+5Cl2

61 WCl5+exc.Xe(OTeF 5)2→W(OTeF 5)6+Xe+52Cl2

62 MoCl5+exc.ClOTeF 5→Mo(OTeF 5)6+5Cl2

Mo­(OTeF_5_)_6_ could be crystallized and presents a monomeric structure, in which the Mo center exhibits an octahedral {MoO_6_} core (see Figure 23).^240^ Similarly to the tungsten compound, decomposition to the oxo compound MoO­(OTeF_5_)_4_ also occurs, in this case at 0 °C over a period of 1 week.^240^

**Figure 23:**
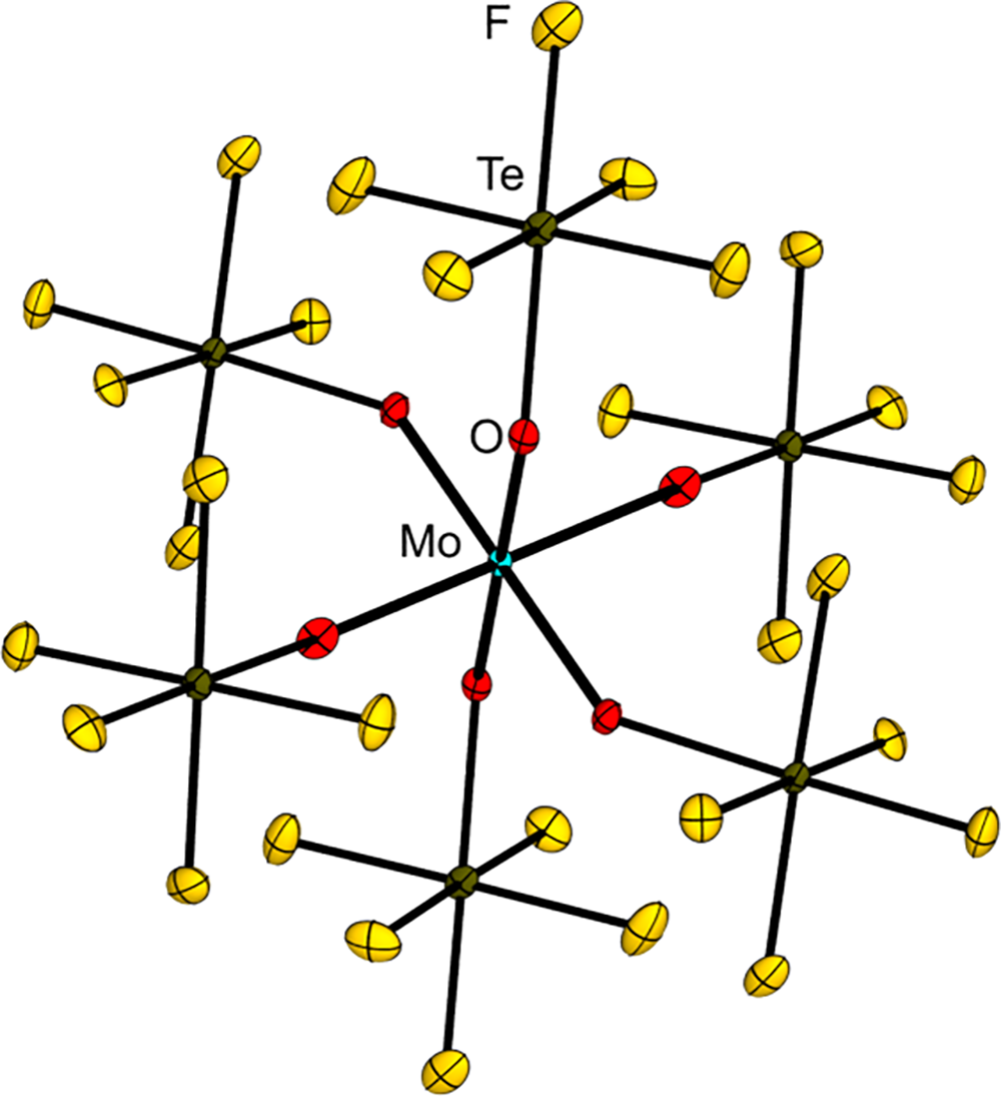
Molecular structure of Mo­(OTeF_5_)_6_ in the solid state. Thermal ellipsoids are set at 50% probability.^240^

Apart from Ti­(OTeF_5_)_4_, the other neutral 3d metal teflate complex known is Fe­(OTeF_5_)_3_, which is formed by reaction of FeCl_3_ with ClOTeF_5_ in SO_2_ClF or C_4_F_9_SO_2_F (see eq 59).^25^ A trigonal planar structure was assumed for this compound based on the large quadrupole splitting in the ^57^Fe Mössbauer spectrum. Nevertheless, crystallization from SO_2_ClF solutions renders the adduct *fac*-[Fe­(OTeF_5_)_3_·3SO_2_ClF], which shows a distorted octahedral {FeO_6_} coordination environment.^25^ Similarly, the adduct *fac*-[Fe­(OTeF_5_)_3_·3SO_2_] can be crystallized from SO_2_ solutions.^27^ The compound is extremely reactive and is only stable toward alkanes and perfluoroalkanes. Attempts of coordinating P­(OEt)_3_ in SO_2_ClF only led to the formation of [P­(OEt)_4_]​[Te_2_Cl_9_] and iron fluorides.^25^


63 FeCl3+3ClOTeF 5→Fe(OTeF 5)3+3Cl2

Despite the success with early transition metals, the attainment of late transition metal teflate species in high oxidation states has not been possible yet. Although reactions between IrF_*x*_ (*x* = 5, 6) or PtF_*x*_ (*x* = 4, 6) and B­(OTeF_5_)_3_ were attempted, they were reported to be unsuccessful.^142^ Furthermore, Hg­(IV) teflate species, namely Hg­(OTeF_5_)_4_ and *cis*- and *trans*-HgF_2_(OTeF_5_)_2_ have been calculated to be thermochemically unstable, yet might be synthetically accessible if the activation barriers for the reductive elimination leading to decomposition are sufficiently high. In this way, suggestions for experimental preparation of such complexes are provided based on the use of the known Hg­(OTeF_5_)_2_ and a suitable oxidizer and/or teflate transfer reagent, such as e.g. Xe­(OTeF_5_)_2_, Xe­(OTeF_5_)_4_ or [XeOTeF_5_]​[Sb­(OTeF_5_)_6_].^234^

#### 11.1.2 Anionic Complexes

A variety of anionic teflate complexes are known, but only recently additional 3d metal complexes of Mn, Co and Ni have been reported and finally used to study the ligand properties of the OTeF_5_ group in transition metal complexes. Group 4 [M­(OTeF_5_)_6_]^2–^ dianions (M = Ti, Zr, Hf) were prepared by Strauss et al. with the objective of accessing stable, soluble and easy-to-prepare WCAs. They were obtained as their silver salts from the metal chlorides MCl_4_ and AgOTeF_5_ (see eq 60).^64,189^ In the case of Ti, F-113 was used as a solvent, whereas for Zr and Hf, 1,2-dichloroethane or dichloromethane and higher temperatures were required. In the case of Ag_2_[Ti­(OTeF_5_)_6_], the reaction with [N­(*n*Bu)_4_]­Cl in dichloromethane yielded [N­(*n*Bu)_4_]_2_[Ti­(OTeF_5_)_6_] (see eq 61), whereas by simply dissolving it in DCM and concentrating the solution, crystals of [Ag­(CH_2_Cl_2_)_3_]_2_[Ti­(OTeF_5_)_6_] were obtained, which readily lose the DCM at room temperature (see eq 62). The anion shows an octahedral structure, and each Ag­(I) ion is coordinated in a bidentate manner by the three CH_2_Cl_2_ molecules. The anion and the cation only interact through two weak Ag–F contacts. Compound Ag_2_[Ti­(OTeF_5_)_6_] is also able to reversibly absorb CO gas to yield the [Ag­(CO)_*n*_]_2_[Ti­(OTeF_5_)_6_] salts (*n* = 1, 2, eq 63), as occurs also with Ag_2_[Zn­(OTeF_5_)_4_] and Ag_2_[B­(OTeF_5_)_4_].^147,148^ Finally, Cs_2_[Ti­(OTeF_5_)_6_] can also be prepared by reaction of Ti­(OTeF_5_)_4_ with 2 equiv of CsOTeF_5_ in HOTeF_5_ as solvent.^239^


64 MCl4→−4AgCl6AgOTeF 5Ag2[M(OTeF 5)6]M=Ti,Zr,Hf

65 Ag2[Ti(OTeF 5)6]→−2AgCl2[N(nBu)4]Cl[N(nBu)4]2[Ti(OTeF 5)6]

66 Ag2[Ti(OTeF 5)6]⇌DCM[Ag(CH2Cl2)3]2[Ti(OTeF 5)6]

67 Ag2[Ti(OTeF 5)6]⇌CO[Ag(CO)n]2[Ti(OTeF 5)6]n=1,2

Contrary to the [M­(OTeF_5_)_6_]^2–^ species (M = Ti, Zr, Hf), which do not perfectly fit the criteria of an ideal WCA because of their double negative charge,^62^ the [Nb­(OTeF_5_)_6_]^−^ anion can be properly considered as such. It can be prepared under the same conditions as the [Sb­(OTeF_5_)_6_]^−^ and [Ti­(OTeF_5_)_6_]^2–^ anions, starting from NbCl_5_ and AgOTeF_5_ in F-113 (see eq 64).^64^ Dissolution of Ag­[Nb­(OTeF_5_)_6_] in CH_2_Br_2_ also results in the related [Ag­(CH_2_Br_2_)_3_]​[Nb­(OTeF_5_)_6_] (see eq 65), which consists of separate [Ag­(CH_2_Br_2_)_3_]^+^ cations (also with bidentate CH_2_Br_2_ ligands) and octahedral [Nb­(OTeF_5_)_6_]^−^ anions, but with no significant interaction between them.^189^ Also this niobium-based WCA can stabilize the previously mentioned silver carbonyl cations [Ag­(CO)_*n*_]^+^ (*n* = 1, 2, eq 66).^147^ Additionally, at high pressures of CO, Ag­[Nb­(OTeF_5_)_6_] can take even a third equivalent of CO, both in the solid state and in F-113 to form [Ag­(CO)_3_]​[Nb­(OTeF_5_)_6_], from which the CO ligands can be removed under vacuum at room temperature but not at −78 °C.^241^ A metathesis reaction with [N­(*n*Bu)_4_]Cl and CClPh_3_ in dichloromethane yields [N­(*n*Bu)_4_]​[Nb­(OTeF_5_)_6_] and [CPh_3_]​[Nb­(OTeF_5_)_6_], respectively (see eq 67).^189^ Nevertheless, the [Nb­(OTeF_5_)_6_]^−^ anion does not seem to be as useful as a WCA compared to [Sb­(OTeF_5_)_6_]^−^, since contrary to the latter it is not stable in acetonitrile or in the presence of nucleophiles such as [OTeF_5_]^−^ or [PF_6_]^−^. By means of isotope scrambling experiments with H^18^OTeF_5_ and Ag^18^OTeF_5_, it was concluded that the [Nb­(OTeF_5_)_6_]^−^ anion is more stable toward electrophiles than [Ti­(OTeF_5_)_6_]^2–^ or [B­(OTeF_5_)_4_]^−^.^64,189^


68 NbCl5→−5AgCl6AgOTeF 5Ag[Nb(OTeF 5)6]

69 Ag[Nb(OTeF 5)6]→CH2Br 2[Ag(CH2Br2)3][Nb(OTeF 5)6]

70 Ag[Nb(OTeF 5)6]⇌CO[Ag(CO)n][Nb(OTeF 5)6]n=1,2,3

71 Ag[Nb(OTeF 5)6]→−AgCl[cat]Cl[cat][Nb(OTeF 5)6]cat=N(nBu)4,CPh3

Both the [Nb­(OTeF_5_)_6_]^−^ and the [Ta­(OTeF_5_)_6_]^−^ anions have also been prepared as their Cs^+^, [NEt_4_]^+^ and [N­(*n*Bu)_4_]^+^ salts by reaction of the neutral M­(OTeF_5_)_5_ complexes (M = Nb, Ta) and the corresponding [cat]​[OTeF_5_] salt (cat = Cs, [NEt_4_], [N­(*n*Bu)_4_]) in HOTeF_5_ as the solvent.^65^ This synthetic method for the [Nb­(OTeF_5_)_6_]^−^ anion is not as straightforward as the one using AgOTeF_5_ ^189^ (see eq 64) and requires an overall larger amount of HOTeF_5_.^65^ Nevertheless, it provides access to the [Ta­(OTeF_5_)_6_]^−^ anion, which could not be obtained as the Ag^+^ salt by using the first procedure (see eq 64).^189^ The electrochemical behavior of the ammonium salts of [Nb­(OTeF_5_)_6_]^−^ and [Ta­(OTeF_5_)_6_]^−^ was also investigated, showing in both cases a quasi-reversible reduction of the transition metal from the oxidation state +V to +IV at −0.69 V (DCM) and −0.60 V (MeCN) in the case of Nb and at −1.52 V (DCM) and −1.42 V (MeCN) in the case of Ta. Additionally, at −1.9 V (DCM) and −2.3 V (MeCN), the 2-electron reduction of the Te atom of the teflate ligands occurs.^65^ All in all, it was concluded by comparison to the analogous fluoro- and chlorometallates, that the teflate is better in stabilizing high oxidation states than the chloride, but not as good as the fluoride.^65^

Four-coordinate teflate dianions have also been demonstrated to stabilize highly unstable cations. The [Zn­(OTeF_5_)_4_]^2–^ dianion was prepared as its Ag^+^ salt^242^ and reacts with CO reversibly, similar to the case of Ag­[B­(OTeF_5_)_4_] to render the [Ag­(CO)_*n*_]_2_[Zn­(OTeF_5_)_4_] salts (*n* = 1, 2), depending on the pressure of CO.^147,148^ The square planar [Pd­(OTeF_5_)_4_]^2–^ anion in Ag_2_[Pd­(OTeF_5_)_4_] was prepared by reaction of PdCl_2_ and AgOTeF_5_ in dichloromethane or 1,2-dichloroethane.^235,243^ The Ag^+^ cation reversibly coordinates dichloromethane or 1,2-dichloroethane. In fact, these compounds were useful to show that both dichloromethane and 1,2-dichloroethane can actually behave as coordinating solvents.^235^ Both salts [Ag­(CH_2_Cl_2_)_2_]_2_[Pd­(OTeF_5_)_4_] and [Ag­(1,2-C_2_H_4_Cl_2_)_2_]_2_[Pd­(OTeF_5_)_4_] were crystallized and their molecular structures analyzed by XRD methods. The {PdO_4_} core exhibits a planar coordination, in which two oxygen atoms in *cis* are bridged by a [Ag­(solv)_2_]^+^ unit (solv = CH_2_Cl_2_, 1,2-C_2_H_4_Cl_2_), or alternatively, it can be seen as the {PdO_4_} core bridging two of the [AgL_2_]^+^ units. Therefore, each Ag­(I) cation is coordinated by two O atoms and four Cl atoms. When 2 equiv of AgOTeF_5_ and 2 equiv of [N­(*n*Bu)_4_]​[OTeF_5_] are used, the compound [N­(*n*Bu)_4_]_2_[Pd­(OTeF_5_)_4_] is obtained.^235^

The [M­(OTeF_5_)_4_]^2–^ anions (M = Ni, Co, Cu) were mentioned, among others, as potential WCAs already in 1993,^59^ yet their use as such has not been demonstrated. The syntheses of the [M­(OTeF_5_)_4_]^2–^ anions (M = Ni, Co) were reported only recently and the Cu species is still unknown. The salts [NEt_4_]_2_[Ni­(OTeF_5_)_4_] and [NEt_4_]_2_[Co­(OTeF_5_)_4_] are prepared by reaction of the corresponding [NEt_4_]_2_[MCl_4_] salt (M = Ni, Co) and neat ClOTeF_5_ as the teflate transfer reagent (see eq 68) accompanied by the release of Cl_2_, which is removed from the reaction mixture under vacuum.^34,36^ Interestingly, with these homoleptic 3d metal complexes in hand, the properties of the teflate ligand in coordination chemistry have been now systematically investigated (see Section 2.3.3).


72 [NEt4]2[MCl4]→−4Cl2exc.ClOTeF 5[NEt4]2[M(OTeF 5)4]M=Ni,Co

Both compounds exhibit a pseudo tetrahedral structure at the metal center, yet more distorted for the Ni (τ_4_ = 0.75) than for the Co (τ_4_ = 0.88) compound.^34,36^ [Note: The four-coordinate geometry index is used to describe the distortion of a four-coordinate complex and is defined as τ_4_ = [360° – (α + β)]/141°, where α and β are the two largest angles in the four-coordinate species. The values of τ_4_ range from 1.00 for a perfect tetrahedral geometry, to 0.00 for a perfect square planar geometry.^244^] In both cases, they represent analogues of the polymeric [MF_4_]^2–^ species (M = Ni, Co) but with monomeric structures (see Figure 24, left), therefore rendering better solubility in organic solvents. They are similarly stable under inert conditions and start decomposing above 220 and 248 °C, respectively. However, at ambient conditions, the high sensitivity of the Ni compound is in contrast to the highly stable Co species. Additionally, [NEt_4_]_2_[Ni­(OTeF_5_)_4_] dissociates all the teflate ligands when dissolved in acetonitrile, yielding [Ni­(MeCN)_6_]​[OTeF_5_]_2_.^34^

**Figure 24:**
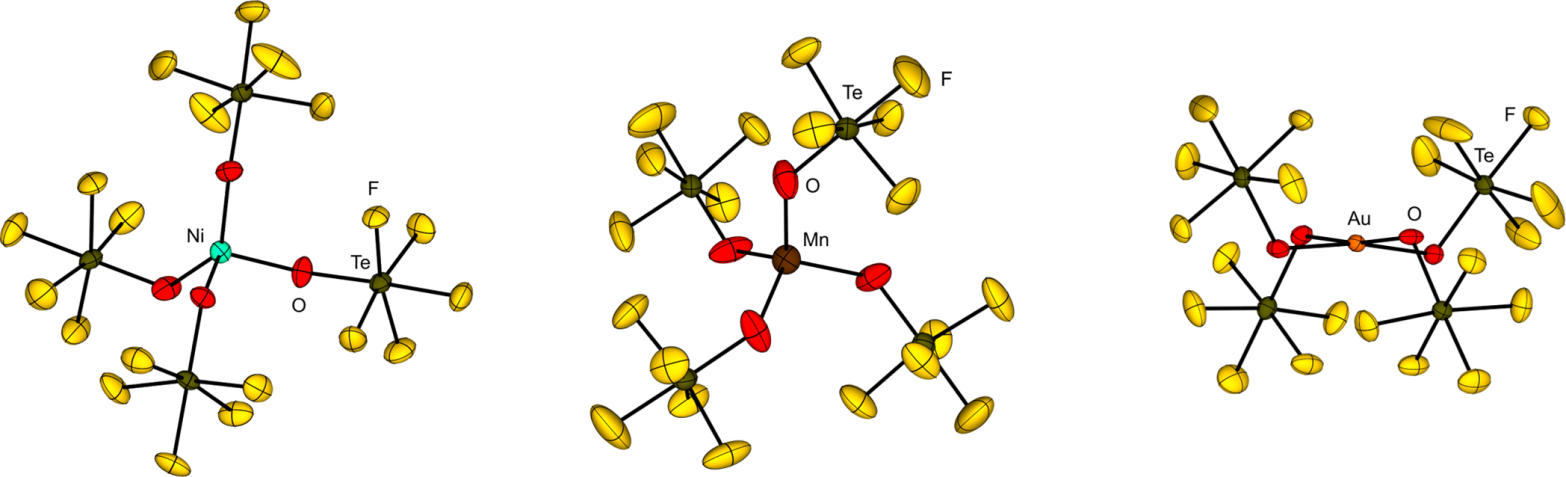
Molecular structure of [NEt_4_]_2_[Ni­(OTeF_5_)_4_] (left), [PPh_4_]_2_[Mn­(OTeF_5_)_4_] (middle), [NEt_3_Me]​[Au­(OTeF_5_)_4_] (right) in the solid state.^24,34,35^ The cations have been omitted for clarity. Thermal ellipsoids set at 50% probability.

Complex [NEt_4_]_2_[Ni­(OTeF_5_)_4_] was determined to be a high-spin Ni­(II) complex with a tetrahedral structure and weak-field/medium-field ligands, whose effective magnetic moment allowed to infer this character for the teflate group (see Section 2.3.3).^34^ Similarly, the presence of three unpaired electrons at the Co­(II) center of [NEt_4_]_2_[Co­(OTeF_5_)_4_] could be derived from the measured effective magnetic moment, and was further supported by electronic spectroscopy and computational calculations.^36^ Interestingly, [NEt_4_]_2_[Co­(OTeF_5_)_4_] is the only group 9 metal teflate prepared thus far.^36^

More recently, some unusual geometries in homoleptic Mn species have been reported.^35^ The high-spin complex [NEt_4_]_2_[Mn­(OTeF_5_)_4_] was prepared by reaction of [NEt_4_]_2_[MnCl_4_] and AgOTeF_5_ (see Scheme [Fig newsch11]).

**11 newsch11:**
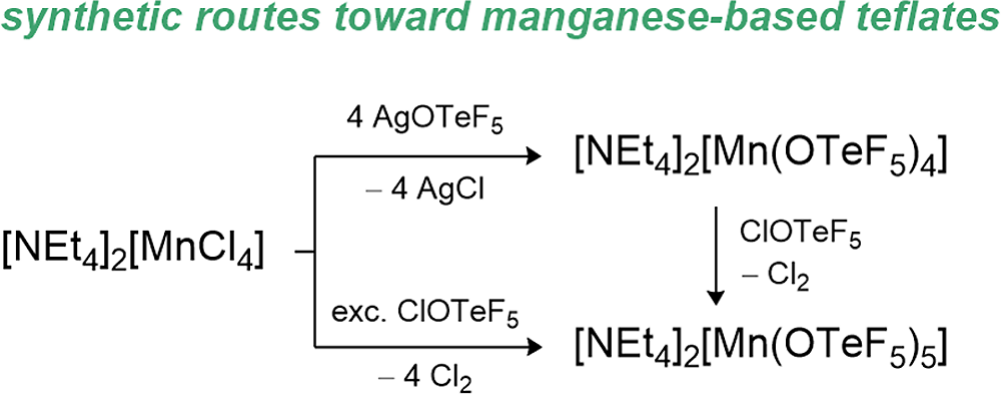
Syntheses of High-Spin Complexes [NEt_4_]_2_[Mn­(OTeF_5_)_4_] and [NEt_4_]_2_[Mn­(OTeF_5_)_5_]

The anion [Mn­(OTeF_5_)_4_]^2–^, which is the first homoleptic mononuclear Mn­(II) center with a {MnO_4_} core and monodentate O-donor ligands, exhibits a tetrahedral structure (τ_4_ = 0.96), as found out by X-ray diffraction analysis of the [PPh_4_]^+^ salt (see Figure 24, middle) and belongs to the scarce number of structurally characterized tetrahedrally coordinated teflate dianionic complexes, together with [Ni­(OTeF_5_)_4_]^2–^, [Co­(OTeF_5_)_4_]^2–^ and [Hg­(OTeF_5_)_4_]^2–^. This compound can be oxidized with neat ClOTeF_5_ to yield [NEt_4_]_2_[Mn­(OTeF_5_)_5_], a salt that can also be prepared starting from [NEt_4_]_2_[MnCl_4_] and an excess of ClOTeF_5_ (see Scheme [Fig newsch11]). A high-spin d^4^ configuration was found for this square pyramidally coordinated Mn­(III) complex anion, which is again the first homoleptic example with all monodentate O-donor ligands. As Mn­(II) and Mn­(III) complexes with O-donor ligands are normally heteroleptic mononuclear species or polynuclear species, these two compounds showcase unique coordination environments thanks to the combination of the similar electronic properties of the teflate and the fluoride ligands, together with the lower tendency of the teflate group to bridge metal centers (note the polymeric nature of the related fluoromanganates).^35^

Unlike the corresponding Lewis acid, the Au anion [Au­(OTeF_5_)_4_]^−^ was only recently synthesized by reaction of different [cat]​[AuCl_4_] salts (cat = Cs, [NMe_4_], [NEt_3_Me]) with neat ClOTeF_5_.^24^ The reaction proceeds via a stepwise substitution of the chloride ligands, as proven not only by quantum-chemical calculations, but also by the crystallization of the [AuCl_3_(OTeF_5_)]^−^ anion when the reaction is performed in DCM (see Section 11.2.1). The [Au­(OTeF_5_)_4_]^−^ anion gives an unusual pattern in the ^19^F NMR spectrum with collapsed signals for the typical AB_4_ spin system of the teflate group and exhibits a distorted square planar structure (τ_4_ = 0.13), as found in the molecular structure in the solid state of the [NEt_3_Me]^+^ salt (see Figure 24, right). Targeting the stabilization of a Au­(V) teflate species, both the reactions of Cs­[AuF_6_] with B­(OTeF_5_)_3_ or ClOTeF_5_ as the teflate transfer reagents were reported to be unsuccessful.^24^ Another approach involving the oxidation of [NMe_4_]​[Au­(OTeF_5_)_4_] with strong oxidizers like Xe­(OTeF_5_)_2_ or even diluted F_2_ (10% in Ar) did not lead to any Au­(V) species.^24^

Finally, a handful of teflate anions of Hg­(II) with a variety of structural motifs have been reported by the group of Schrobilgen. They were prepared using Hg­(OTeF_5_)_2_ and [cat]​[OTeF_5_] ([cat] = NMe_4_, NEt_4_, Cs) as starting materials in different molar ratios in SO_2_ClF (see eqs 70–73).^245^ This way, the discrete tetrahedrally distorted [Hg­(OTeF_5_)_4_]^2–^ anion (τ_4_ = 0.79) of the [NEt_4_]^+^ salt is isolated, whereas in compound Cs_2_[Hg­(OTeF_5_)_4_]·Hg­(OTeF_5_)_2_ the tetrahedron is flattened (τ_4_ = 0.53) due to the interactions of the anion with neutral Hg­(OTeF_5_)_2_, resulting in fact in a chain structure. The [Hg­(OTeF_5_)_5_]^3–^ anion was the first teflate trianion to be isolated, both with [NMe_4_]^+^ and [NEt_4_]^+^ as counterions. A square pyramidal geometry was observed for the [NMe_4_]^+^ salt (τ_5_ = 0.09), whereas for the [NEt_4_]^+^ salt the geometries for the two crystallographic distinct anions are intermediate (τ_5_ = 0.52 and 0.26) between a square pyramid and a trigonal bipyramid, which is the optimized structure in the gas phase (τ_5_ = 0.91). [Note: The five-coordinate geometry index is used to describe the distortion of a five-coordinate complex and is defined as τ_5_ = (β – α)/60°, where α and β are the two largest angles in the five-coordinate species. The values of τ_5_ range from 1.00 for a perfect trigonal bipyramidal geometry, to 0.00 for a perfect square pyramidal geometry.^246^] Long Hg–F intraionic contacts *trans* to the apical oxygen were suggested to favor the observed square pyramidal geometry. The synthesis of monomeric [Hg­(OTeF_5_)_3_]^−^ anion was not possible, yet the dimer [Hg_2_(OTeF_5_)_6_]^2–^ could be prepared as the [NMe_4_]^+^ salt. In the solid state it contains Hg­(OTeF_5_)_2_ units bridged by two teflate groups, therefore rendering an overall butterfly shaped {HgO_μ_}_2_ core. Finally, the [Hg_2_(OTeF_5_)_7_]^3–^ anion in the crystal of {Cs_3_[Hg_2_(OTeF_5_)_7_]·Hg­(OTeF_5_)_2_}·4SO_2_ClF was formulated as two [Hg­(OTeF_5_)_3_]^−^ anions bridged by one teflate anion. This compound possesses a chain structure, where Hg­(OTeF_5_)_2_ entities alternate with [Hg_2_(OTeF_5_)_7_]^3–^ anions. In all nonmonomeric structures, Hg centers are stabilized through additional interionic contacts with O and F atoms. The NBO analyses performed on the monomeric species [Hg­(OTeF_5_)_4_]^2–^ and [Hg­(OTeF_5_)_5_]^3–^ concluded that the Hg–O bonds are more polar than in neutral Hg­(OTeF_5_)_2_.^245^ Homoleptic transition metal compounds are gathered in Table 10.


73 Hg(OTeF 5)2+n[cat][OTeF 5]→[cat]n[Hg(OTeF 5)2+n]n=2,cat=NEt4n=3,cat=NEt4,NMe4

74 2Hg(OTeF 5)2+2[NMe4][OTeF 5]→[NMe4]2[Hg2(OTeF 5)6]

75 2Hg(OTeF 5)2+2CsOTeF 5→Cs2[Hg(OTeF 5)4]·Hg(OTeF 5)2

76 3Hg(OTeF 5)2+3CsOTeF 5→Cs3[Hg2(OTeF 5)7]·Hg(OTeF 5)2

**Table 10:** Overview of Homoleptic Transition Metal Compounds

Element	Ox	Compound	Refs
Ti	IV	Ti(OTeF_5_)_4_	^42,239^
		[cat]_2_[Ti(OTeF_5_)_6_] (cat = Ag, Ag(DCM)_3_, Ag(CO), Ag(CO)_2_, Cs, N(*n*Bu)_4_)	^64,147,148,189,239^

Zr	IV	Ag_2_[Zr(OTeF_5_)_6_]	^189^

Hf	IV	Ag_2_[Hf(OTeF_5_)_6_]	^189^

Nb	V	Nb(OTeF_5_)_5_	^65,142^
		[cat]​[Nb(OTeF_5_)_6_] (cat = Ag, Ag(CH_2_Br_2_)_3_, Ag(CO), Ag(CO)_2_, Ag(CO)_3_, CPh_3_, Cs, N(*n*Bu)_4_, NEt_4_)	^64,65,147,189,241^

Ta	V	[Ta(OTeF_5_)_5_]	^65,142^
		[cat]​[Ta(OTeF_5_)_6_] (cat = Cs, NEt_4_, N(*n*Bu)_4_)	^65,142^

Mo	VI	Mo(OTeF_5_)_6_	^240^

W	V	W(OTeF_5_)_5_	^240^
	VI	W(OTeF_5_)_6_	^142,240^

Mn	II	[cat]_2_[Mn(OTeF)_4_] (cat = NEt_4_, PPh_4_)	^35^
	III	[NEt_4_]_2_[Mn(OTeF_5_)_5_]	^35^

Fe	III	Fe(OTeF_5_)_3_	^25^

Co	II	[NEt_4_]_2_[Co(OTeF_5_)_4_]	^36^

Ni	II	[NEt_4_]_2_[Ni(OTeF_5_)_4_]	^34^

Pd	II	[cat]_2_[Pd(OTeF_5_)_4_] (cat = Ag(DCM)_2_, Ag(DCE)_2_, N(*n*Bu)_4_)	^235,243^

Cu	I	CuOSF_5__(solv)_	^110^

Ag	I	AgOTeF_5_	^10,165,235^
		AgOSF_5__(solv)_	^110^

Au	III	Au(OTeF_5_)_3_	^24,40^
		[cat]Au(OTeF_5_)_4_ (cat = Cs, NMe_4_, NEt_3_Me)	^24^

Zn	II	[cat]​[Zn(OTeF_5_)_4_] (cat = Ag, Ag(CO), Ag(CO)_2_)	^147,178,242^

Hg	II	Hg(OTeF_5_)_2_	^5,11,233^
		[NEt_4_]_2_[Hg(OTeF_5_)_4_]	^245^
		[cat]_3_[Hg(OTeF_5_)_5_] (cat = NMe_4_, NEt_4_)	^245^
		[NMe_4_]_2_[Hg_2_(OTeF_5_)_6_]	^245^
		Cs_2_[Hg(OTeF_5_)_4_]·Hg(OTeF_5_)_2_	^245^
		Cs_3_[Hg_2_(OTeF_5_)_7_]·Hg(OTeF_5_)_2_	^245^

### 11.2 Heteroleptic *d*-Block Metal Teflates

#### 11.2.1 Metal Teflate Complexes Containing Oxo Ligands

By attempting to synthesize homoleptic metal teflates using the versatile approach of reacting metal fluorides with B­(OTeF_5_)_3_, several heteroleptic metal teflate complexes containing an additional oxo ligand were discovered. The first among these compounds, MoOF_3_(OTeF_5_), was synthesized by the neat reaction of MoF_6_ with B­(OTeF_5_)_3_ at 80 °C. Initially, MoF_4_(OTeF_5_)_2_ is formed, followed by the elimination of TeF_6_ through an internal fluorination process, resulting in the formation of the oxofluoride complex (see eq 74). Starting with molybdenum oxytetrafluoride (MoOF_4_) instead of MoF_6_, a direct synthesis of MoOF_3_(OTeF_5_) was achieved by reaction with B­(OTeF_5_)_3_ (see eq 74a).^247^


MoF 6→−BF3B(OTeF 5)3MoOF 3(OTeF 5) 77

MoOF 4→−BF3B(OTeF 5)3MoOF 3(OTeF 5) 78

The addition of F-114 as a solvent, along with a decrease of the reaction temperature to 50 °C, leads to the formation of MoO­(OTeF_5_)_4_ from MoF_6_ (see Scheme 12). This reactivity differs from the behavior of the heavier homologue WF_5_, which, under similar conditions, forms the homoleptic W­(OTeF_5_)_6_ (see Section 11.1).^142^ The tetragonal pyramidal coordination sphere observed around the central molybdenum atom in MoO­(OTeF_5_)_4_ is surprising when compared to the trigonal bipyramidal structure of the analogous MoO­[OC­(CF)_3_]_4_.^142,248^ This unexpected structural difference may be attributed to an intermolecular Mo–F interaction that complete the octahedral coordination.^142^ Furthermore, MoO­(OTeF_5_)_4_ is the decomposition product obtained when storing Mo­(OTeF_5_)_6_ above −30 °C.

**Scheme 12:**
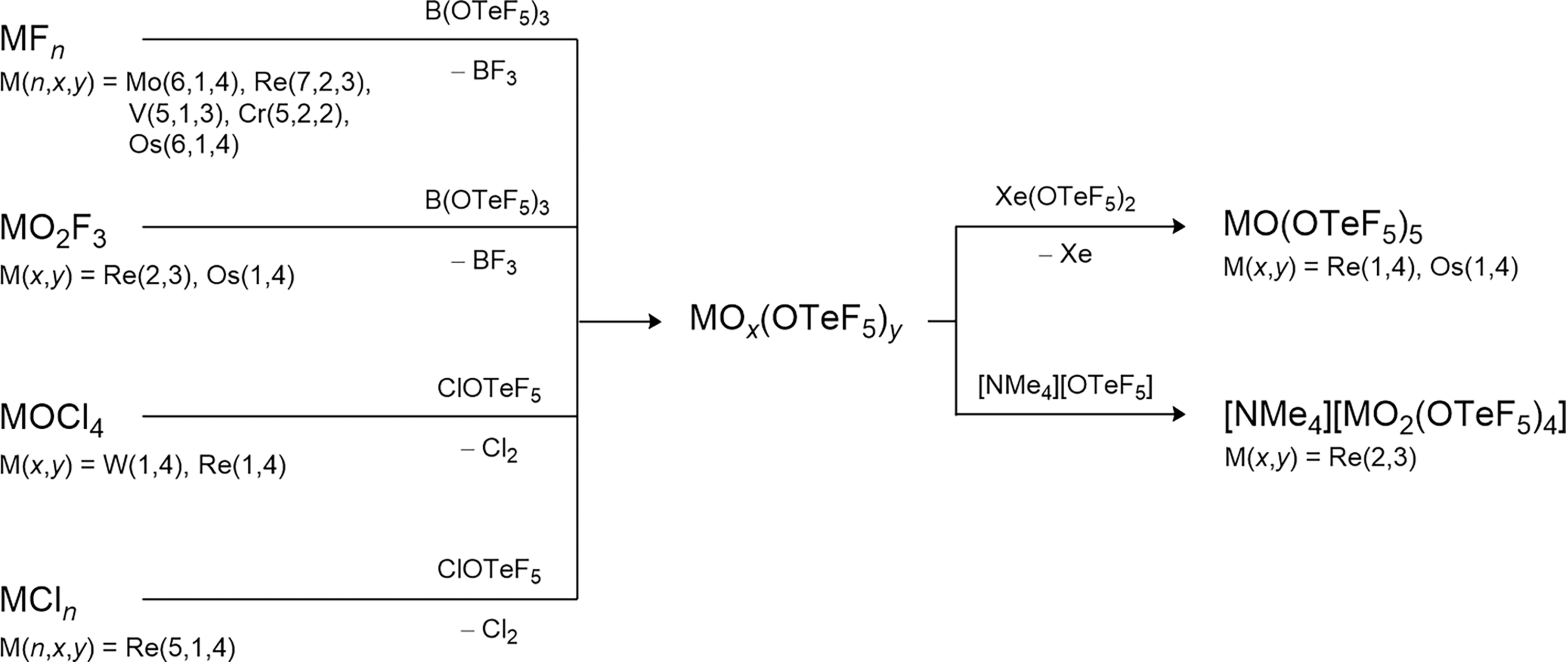
Syntheses of Various Metal Teflate Complexes Containing Oxo Ligands

Likewise, W­(OTeF_5_)_5_ decomposes over time, even at −78 °C, to yield the structurally similar compound WO­(OTeF_5_)_4_. Nevertheless, a more efficient method for obtaining the latter involves reacting WOCl_4_ with ClOTeF_5_.^240^

Similarly, when reacting ReF_7_ or ReF_6_ with B­(OTeF_5_)_3_ in CFCl_3_, the desired homoleptic rhenium complexes could not be obtained; instead, the yellow liquid ReO_2_(OTeF_5_)_3_ was formed (see Scheme 12).^142^ Pure ReO_2_F_3_ also produces the aforementioned compound in a reaction with B­(OTeF_5_)_3_ in F-114 (see Scheme 12).^249^ The molecular structure of this compound in the solid state could not be determined, but the teflate groups were shown to be fluxional on the NMR time scale, indicative of a trigonal bipyramid where the oxygen atoms occupy the equatorial plane. Additionally, recorded vibrational spectra are in agreement with the proposed structure. The acceptor ability of the rhenium center was probed by addition of [NMe_4_]​[OTeF_5_], leading to the formation of [NMe_4_]​[ReO_2_(OTeF_5_)_4_] (see Scheme 12). The structure exhibits an approximately octahedral coordination around the rhenium, exclusively adopting the *cis*-dioxo isomer both in solution and in the solid state (see Figure 25).^249^ Both ReCl_5_ and ReOCl_4_ react with ClOTeF_5_ to form ReO­(OTeF_5_)_4_ (see Scheme 12), a compound structurally similar to MoO­(OTeF_5_)_4_. ReO­(OTeF_5_)_4_ can be further oxidized by Xe­(OTeF_5_)_2_ to yield the Re­(VII) species ReO­(OTeF_5_)_5_.^250^ The structure of this compound exhibits the anticipated octahedral coordination and serves as another example of a bonding scenario that can otherwise only be achieved with fluorido ligands.

**Figure 25:**
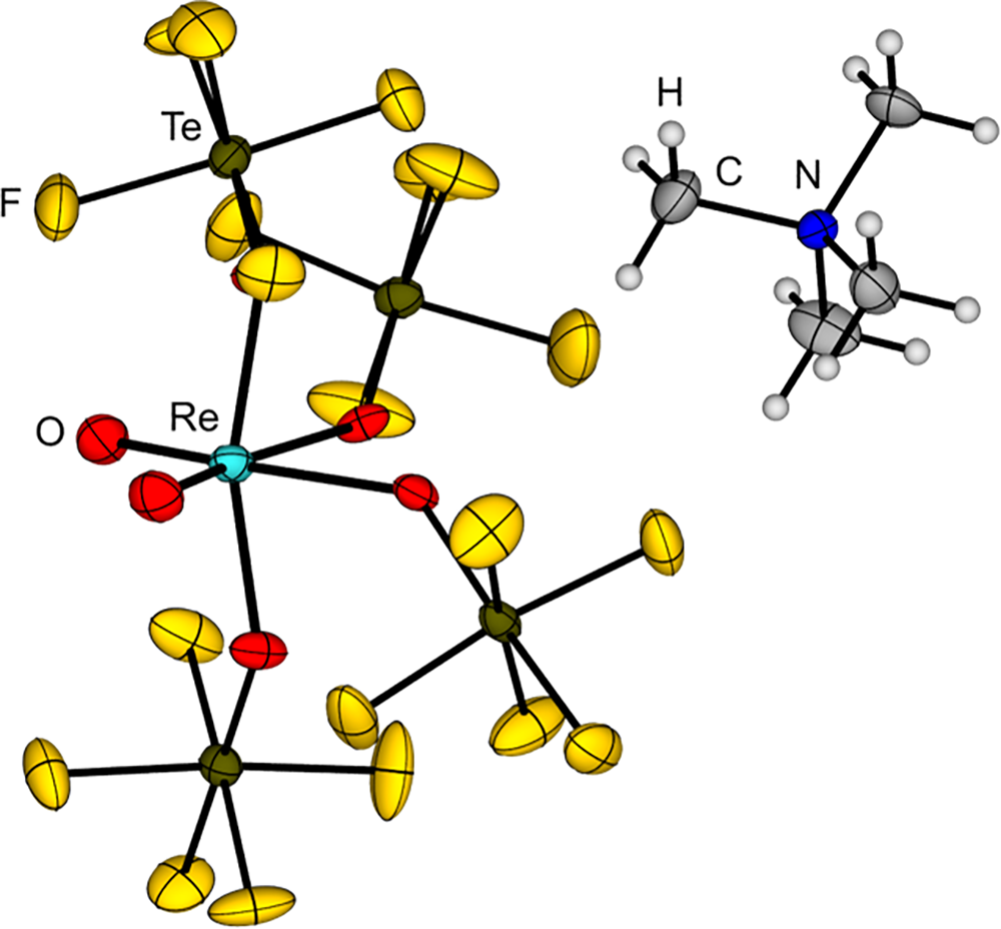
Molecular structure in the solid state of [NMe_4_]​[ReO_2_(OTeF_5_)_4_].^249^ Thermal ellipsoids are set at 25% probability.

For osmium, a preferred coordination number of 5 seems evident, as OsF_6_ and OsO_2_F_3_ both react with B­(OTeF_5_)_3_ to render OsO­(OTeF_5_)_4_ (see Scheme 12). However, the similar reaction of octavalent OsO_3_F_2_ only leads to the formation of OsO_4_. In the molecular structure in the solid state of OsO­(OTeF_5_)_4_, similarities with MoO­(OTeF_5_)_4_ and ReO­(OTeF_5_)_4_ are observed. Nevertheless, TeF_4_ cocrystallizes, providing a bridging fluorine atom to complete the octahedral coordination at the Os center. OsO­(OTeF_5_)_4_ can be oxidized by Xe­(OTeF_5_)_2_, similarly to the related Re species, likely resulting in the OsOF_5_ analogue OsO­(OTeF_5_)_5_, although isolation of the latter has been elusive (see Scheme 12).^142,250^

The early transition metal pentafluorides of vanadium (VF_5_) and chromium (CrF_5_) react with B­(OTeF_5_)_3_ to form the corresponding oxo compounds VO­(OTeF_5_)_3_ and CrO_2_­(OTeF_5_)_2_, exhibiting a more stable coordination number of 4 (see Scheme 12). Both complexes are obtained in solution, colorless VO­(OTeF_5_)_3_ being in contrast with the dark red color of CrO_2_(OTeF_5_)_2_.^142^ The lighter chalcogen homologues of these two compounds, VO­(OSeF_5_)_3_ and CrO_2_(OSeF_5_)_2_, can be synthesized by reacting the respective chloride precursors with Hg­(OSeF_5_)_2_.^42^

#### 11.2.2 Metal Teflate Complexes Containing Halide Ligands

Another compound class that was predominantly observed as the main or side product during attempts of synthesizing homoleptic metal teflates are the mixed metal teflate halide complexes, as for instance TiCl_4–*n*_(OTeF_5_)_*n*_ (*n* = 1–3) and W­(OTeF_5_)_*n*_X_6–*n*_ (*n* = 1, 2, 4, 5; X = F, Cl). The first example of such complexes was observed during the reaction of WCl_6_ with Hg­(OTeF_5_)_2_, targeting the homoleptic W­(OTeF_5_)_6_, in a reaction analogous to that of TiCl_4_ (see eq 54). However, the reaction resulted in WCl­(OTeF_5_)_5_, a viscous, distillable liquid (see eq 75). At this time, this compound represented the first complex with more than four pentafluoro­orthochalcogenate ligands.^42^ The usage of ClOTeF_5_ as a teflate transfer reagent instead of Hg­(OTeF_5_)_2_ yields the same product.^142^ The reaction of the fluorinated starting material WF_6_ with B­(OTeF_5_)_3_ results, depending of the stoichiometry of the reaction, in the formation of either the one or the two times substituted compounds WF_5_(OTeF_5_) or WF_4_(OTeF_5_)_2_ (see eq 76).^251^ A second addition of B­(OTeF_5_)_3_ to WF_4_(OTeF_5_)_2_ yields WF_2_(OTeF_5_)_4_ (see eq 76).^142^


79 WCl6→−Cl2orHgCl2ClOTeF 5orHg(OTeF 5)2WCl(OTeF 5)5

80 WF 6→−BF 3B(OTeF 5)3WF 6−n(OTeF 5)nn=1,2,4

The mixed titanium teflate compound TiCl_3_(OTeF_5_) can be synthesized in a unimolecular reaction between TiCl_4_ and HOTeF_5_ with a conversion of 30%. The removal of formed HCl and the addition of new teflic acid leads to the complete conversion of the starting material (see eq 77). In an attempt to remove TiCl_4_ via distillation from the reaction mixture, dismutation equilibria result in the homoleptic Ti­(OTeF_5_)_4_ (see eq 54).^239^ In the case of gold, [AuCl_3_(OTeF_5_)]^−^ (see eq 78) was observed in the reaction of [AuCl_4_]^−^ with ClOTeF_5_ in CH_2_Cl_2_, which underlines the stepwise ligand substitution in the formation of [Au­(OTeF_5_)_4_]^−^ (see Section 11.1).^24^


81 TiCl4→−HClHOTeF 5TiCl3(OTeF 5)

82 [NMe4][AuCl4]→−Cl2ClOTeF 5[NMe4][AuCl3(OTeF 5)]

Furthermore, related to this kind of species, NgF_2_ adducts (Ng = Kr, Xe) of teflate complexes have been also reported. The first complex with a bridging KrF_2_ moiety was reported by Schrobilgen et al. to be [Hg­(OTeF_5_)_2_·1.5 KrF_2_], which was observed by reacting the neutral Hg­(OTeF_5_)_2_ with KrF_2_ at low temperatures in SO_2_ClF (see eq 79).


83 Hg(OTeF 5)2+1.5NgF 2→[Hg(OTeF 5)2·1.5NgF 2]Ng=Kr,Xe

The Hg­(OTeF_5_)_2_ units have *gauche* conformations with intramolecular Hg–O and Hg–F contacts leading to a chain-like structure due to the bridging KrF_2_ units (see Figure 26). An isostructural complex is formed by using XeF_2_ instead of KrF_2_, and shows shorter Hg–F contacts due to the greater ionic character of the Xe–F bond.^233^ It is surprising that the strong oxidizer KrF_2_ is not able to oxidize the mercury further to its highest oxidation state +IV, as shown by isolated HgF_4_ in cryogenic rare gas matrices.^252^

**Figure 26:**
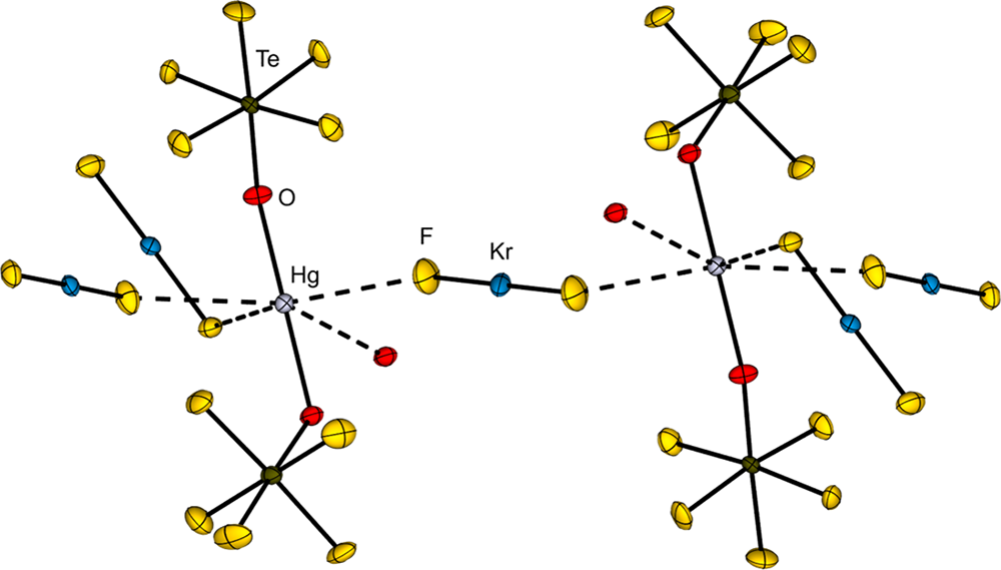
Molecular structure of [Hg­(OTeF_5_)_2_·1.5 KrF_2_] in the solid state.^233^ Thermal ellipsoids are set at 50% probability.

#### 11.2.3 Metal Teflate Complexes Containing O-, N- and P-Donor Ligands

Metal teflate complexes containing additionally O-, N- or P-donor ligands are only known for middle and late *d*-block metals. For iron there are two different types of porphyrin complexes known, namely [Fe­(OEP)​(OTeF_5_)] and [Fe­(TPP)​(OTeF_5_)].^253^ They are synthetically accessible as reported by Strauss et al. through the reaction of either [Fe­(TPP)]_2_O or [Fe­(OEP)]_2_O with teflic acid, whereby water is eliminated (see Scheme 13). Additionally, through treatment of [Fe­(TPP)​(OTeF_5_)] with tetrahydrofuran (THF) the complex [Fe­(TPP)​(OTeF_5_)​(THF)] could be observed, which is a rare example of a six-coordinate high-spin Fe­(III) porphyrin complex. The temperature-dependent ^1^H NMR spectra of the five-coordinate Fe complexes were studied and compared to related chlorido compounds.^254^

**Scheme 13:**
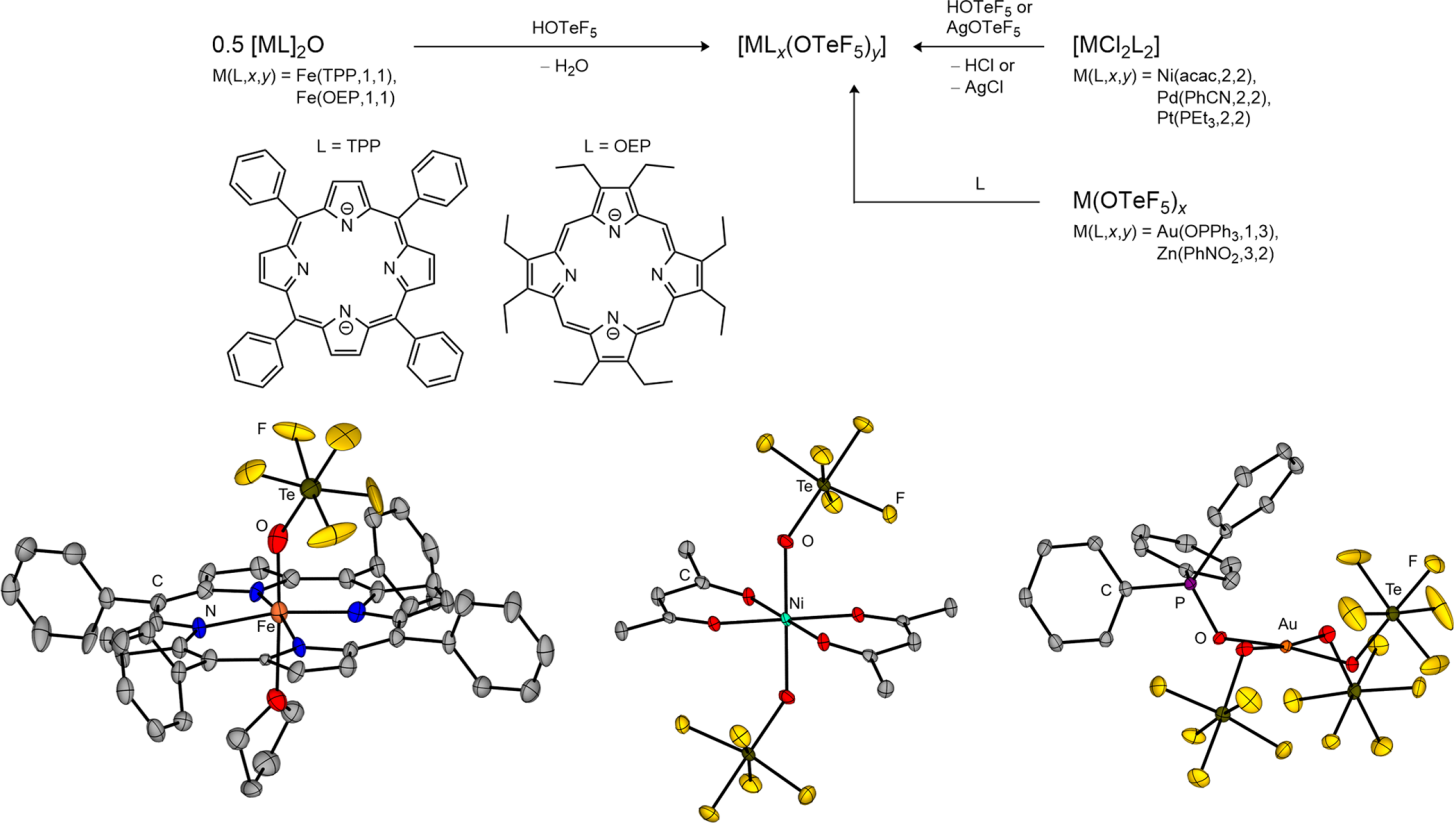
Syntheses of Various Metal Teflate Complexes Containing O-, N- and P-Donor Ligands

For group 10 metals, the first of such complexes, [Pd­(PhCN)_2_(OTeF_5_)_2_], was published in 1987 and prepared by reaction of [PdCl_2_(PhCN)_2_] with AgOTeF_5_ (see Scheme 13). In this work, the incompatibility of the teflate and triphenylphosphine ligands was discussed, arising from the oxidation of PPh_3_ to OPPh_3_ and further decomposition to P–F species and elemental tellurium.^255^ However, it was shown that the incompatibility is only in the case of PPh_3_, since the reaction of *cis*- or *trans*-[PtCl_2_(PEt_3_)_2_] with HOTeF_5_ yielded the corresponding mixed *cis*- or *trans*-[Pt­(OTeF_5_)_2_(PEt_3_)_2_] (see Scheme 13).^256^ Additionally, the PPh_3_ incompatibility seems to be metal specific, since [Re­(CO)_4_(OTeF_5_)​(PPh_3_)] could be isolated (see Section 11.2.4) from the reaction between [Re­(CO)_4_Me­(PPh_3_)] and HOTeF_5_.^257^

The first nickel teflate complex, [Ni­(Hacac)_2_(OTeF_5_)_2_], was prepared by Riedel et al. by reaction of Ni­(acac)_2_ (acac = acetylacetonate) with HOTeF_5_ (see Scheme 13). Both teflate ligands can be easily replaced by stronger coordinating agents, e.g. MeCN, and already small amounts of water lead to the bridged compound [{Ni­(Hacac)​(H_2_O)​(OTeF_5_)​(μ-OTeF_5_)}_2_].^258^ Further nickel­(II) complexes containing the teflate ligand were observed after treatment of the homoleptic [Ni­(OTeF_5_)_4_]^2–^ with either diethyl ether or 2,2′-dimethyl-6,6′-bipyridine (bpyMe_2_) to form [*trans*-Ni­(Et_2_O)_2_(OTeF_5_)_4_]^2–^ or [Ni­(bpyMe_2_)​(OTeF_5_)_3_]^−^, respectively (see eqs 80 and 81). Notably, using 2,2′-bipyridine does not lead to the desired similar product, but forms instead a mixture of species, which could not be separated, and which included the [Ni­(bpy)_3_]^2+^ dicationic complex. This underlines the easiness of dissociation of the weakly coordinated teflate groups in the homoleptic species.^34^


84 [NEt4]2[Ni(OTeF 5)4]→Et2O[NEt4][Ni(OTeF 5)4](OEt2)2]

85 [NEt4]2[Ni(OTeF 5)4]→−[NEt4]OTeF 5bpyMe2[NEt4][[Ni(OTeF 5)3](bpyMe2)]

As part of the investigation of the Lewis acidity of Au­(OTeF_5_)_3_ the Gutmann–Beckett method was applied by addition of OPPh_3_ to the Lewis acid (see Scheme 13). This led to the selective formation of [Au­(OPPh_3_)​(OTeF_5_)_3_], which allowed the first structural investigation of an Au­(OTeF_5_)_3_ adduct in the solid state via XRD.^24^ Interestingly, the molecular structure in the solid state shows that the coordination around the gold­(III) center is slightly distorted from the typical square planar geometry (τ_4_ = 0.10).

The only mixed teflate compounds for group 12 can be prepared as reported by Strauss et al. by reacting ZnEt_2_ with teflic acid in toluene and subsequent treatment with PhNO_2_ or MeNO_2_ (see Scheme 13). The initially formed [Zn­(OTeF_5_)_2_(tol)_*n*_] (*n* = 1.5–2.0) rapidly exchanges the toluene ligands by PhNO_2_ or MeNO_2_ units. The molecular structure in the solid state with PhNO_2_ shows either the dimer [Zn­(PhNO_2_)_2_(OTeF_5_)_2_]_2_ or the monomer [Zn­(PhNO_2_)_3_­(OTeF_5_)_2_]. Interestingly, the first example shows that PhNO_2_ can act as either a bidentate (O,O′) or monodentate (O) ligand.^259^

Using Hg­(OTeF_5_)_2_, donor–acceptor adducts are formed at 0 °C between the Lewis acidic Hg­(II) center and NSF_3_ yielding [Hg­(OTeF_5_)_2_·NSF_3_]_∞_, [Hg­(OTeF_5_)_2_·2NSF_3_]_2_, and [Hg_3_(OTeF_5_)_6_·4NSF_3_], as reported by Schrobilgen et al. At room temperature, a nucleophilic attack of a teflate group at the sulfur­(VI) atom under elimination of TeF_6_ occurs and leads to the structurally related F_2_OSN derivatives. These complexes provide the first examples of NSF_3_ coordination to mercury.^260^

Mixed metal complexes of the lighter teflate homologue pentafluoro­oxosulfate have also been reported. The addition of 2,2′-bipyridine or 4,4′-di-*tert*-butyl-2,2′-bipyridine (bpy­(*t*Bu)_2_) to an AgOSF_5_ solution in MeCN leads to the formation of [Ag­(bpy)_2_(OSF_5_)] or [Ag­(bpy­(*t*Bu)_2_)_2_(OSF_5_)]. In contrast to the aforementioned AgOSF_5_ solution in MeCN, the bipyridine complexes are stable at room temperature under inert conditions. Similarly, the addition of 2,2′-bipyridine to CuOSF_5_ leads to the stable complex [Cu­(bpy)_2_(OSF_5_)]. In a metathesis reaction starting from divalent metal chlorides (CuCl_2_ and NiCl_2_) and AgOSF_5_ under the addition of 2,2′-bipyridine, the respective complexes [M­(bpy)_2_(OSF_5_)_2_] (M = Cu, Ni) are formed. [Ni­(bpy)_2_(OSF_5_)_2_] can also be synthesized by the addition of OSF_4_ to a solution of NiF_2_ and 2,2′-bipyridine in MeCN. The NMR analysis of the metal OSF_5_ complexes indicates that the [OSF_5_]^−^ anion acts as a weakly coordinated ligand, which readily dissociates in MeCN. The molecular structure in the solid state of [Cu­(bpy)_2_(OSF_5_)_2_] shows two different types of OSF_5_ moieties: one is attached to the five-coordinate copper center via its oxygen atom, while the other lies in the cavities of the [Cu­(bpy)_2_(OSF_5_)]^+^ units and shows several H–F contacts for stabilization. The five-coordinate copper center shows a rather rare geometry for Cu­(II) compounds and can be considered as a distorted trigonal bipyramid (τ_5_ = 0.68).^110^

#### 11.2.4 Organometallic Teflate Complexes

There are metal teflate complexes with five different organometallic ligands known: cyclopentadienyl (Cp), CO, N-heterocyclic carbene (NHC), alkene and alkyl ligands. Complexes bearing Cp and teflate ligands are accessible via halide-teflate metathesis reactions of the chloride (M = Ti, Zr, Hf, Mo, W) or fluoride (M = Ti) precursors with an excess of teflic acid, yielding the corresponding M­(Cp)_2_(OTeF_5_)_2_ (M = Ti, Zr, Hf, Mo, W) species (see Scheme 14). Since the molybdenum and tungsten fluoride complexes M­(Cp)_2_F_2_ (M = Mo, W) are unknown, Mo­(Cp)_2_(OTeF_5_)_2_ and W­(Cp)_2_(OTeF_5_)_2_ represent additional examples of metal teflate complexes without metal fluoride counterparts. The high-frequency chemical shifts observed for the Cp protons suggest a strong M–Cp interaction, which together with the significant differences in the p_π_-d_π_ (pseudo)­halide–metal interaction between the teflate and fluoride ligands could explain the failure to isolate the fluoride analogues.^261^ The mixed compound [FeCp­(CO)_2_(OTeF_5_)] can be prepared in the reaction of [FeCp­(CO)_2_Me] with teflic acid under the formation of CH_4_ (see Scheme 14).^262^ A second method to prepare [FeCp­(CO)_2_(OTeF_5_)] involves the reaction of the bromide precursor [FeCp­(CO)_2_Br] with AgOTeF_5_ as the teflate transfer reagent. However, this route is not superior neither in terms of yield nor of purity in comparison to the first method. The more abundant carbonyl complexes can be produced in the same manner with [M­(CO)_5_Me] as starting material in the cases of manganese and rhenium yielding [M­(CO)_5_(OTeF_5_)] (M = Mn, Re) (see Scheme 14).^262,263^ The recorded IR spectra of these compounds show that the metal–oxygen bond has a large degree of ionic character. This high ionic character of the teflate ligands can be confirmed by the long Mn–O bond in the molecular structure of [Mn­(CO)_5_(OTeF_5_)] (see Figure 27).

**Figure 27:**
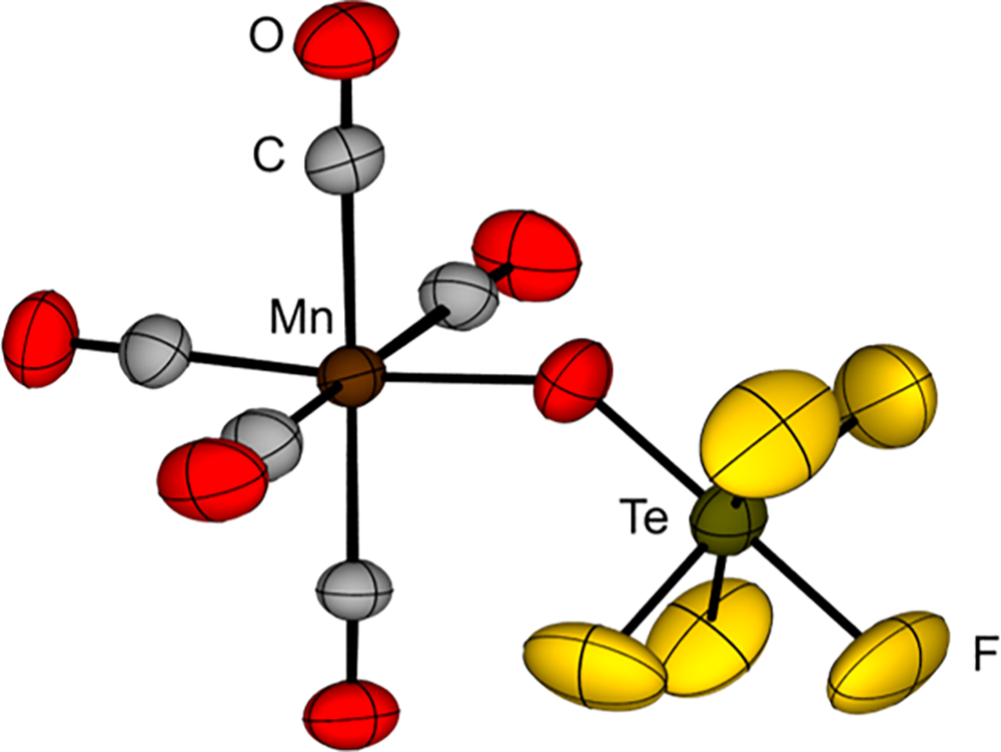
Molecular structure in the solid state of [Mn­(CO)_5_(OTeF_5_)].^263^ Thermal ellipsoids are set at 25% probability.

**Scheme 14:**
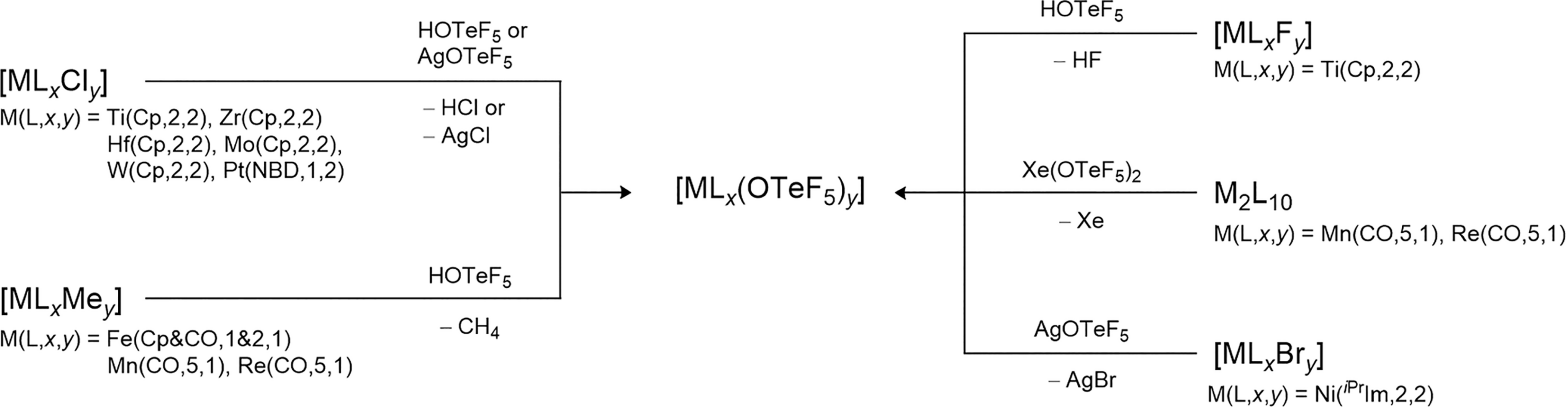
Syntheses of Various Metal Teflate Complexes Containing Organometallic Ligands

Additionally, [M­(CO)_5_(THF)] precursors (M = Mo, W) react with [N­(*n*Bu)_4_]​[OTeF_5_] to form [N­(*n*Bu)_4_]­[M­(CO)_5_(OTeF_5_)], which were only stable for short periods of time in THF. [Mn­(CO)_5_(OTeF_5_)], [Re­(CO)_5_(OTeF_5_)] and [FeCp­(CO)_2_(OTeF_5_)] are stable indefinitely in the solid state but [Mn­(CO)_5_(OTeF_5_)] slowly releases CO in a dichloromethane solution.^262^ The synthesis of [M­(CO)_5_(OEF_5_)] (M = Mn, Re; E = Se, Te) complexes is also possible by oxidation of M_2_(CO)_10_ with Xe­(OEF_5_)_2_ at room temperature in dichloromethane (see Scheme 14).^263^ Here, similar reactions with XeF_2_ result in an uncharacterizable mixture of products for manganese and in the mixed valence [Re­(CO)_5_(μ-F)­ReF_5_] complex for rhenium, which shows the better bridging properties of fluoride in comparison to the teflate ligand. However, this route is not applicable for other low-valent metal complexes like Ru_3_(CO)_12_, Os_3_(CO)_12_, Ir_4_(CO)_12_ or M­(CO)_3_(PPh_3_)_2_ (M = Ru, Os).^108^

As mentioned above, in several cases the addition of HOTeF_5_ to a metal methyl complex results in the formation of the corresponding metal teflate upon release of CH_4_. However, *cis*-[Os­(CO)_4_Me_2_] in combination with HOTeF_5_ does not lead to *cis*-[Os­(CO)_4_(OTeF_5_)_2_], but to the formation of *cis*-[Os­(CO)_4_(OTeF_5_)­Me] (see eq 82).^257^ This underlines the boundaries of the introduction of teflate ligands with this method. Interestingly, teflic acid can be used not only for the introduction of teflate groups to the coordination sphere of a metal compound, but also for the protonation of metal complexes. This way, [HM­(CO)_3_(PPh_3_)_2_]​[OTeF_5_] (M = Fe, Ru, Os) could be obtained from their [M­(CO)_3_(PPh_3_)_2_] precursors and showed the first use of HOTeF_5_ as a protic acid (see eq 83).^264^


86 cis‐[Os(CO)4Me2]→−CH4HOTeF 5cis‐[Os(CO)4(OTeF 5)Me]

87 [M(CO)3(PPh3)2]→HOTeF 5[HM(CO)3(PPh3)2][OTeF 5]M=Fe,Ru,Os

The abundance of compounds of the other aforementioned organometallic metal teflate complex classes is scarce. For the alkene ligands only one complex bearing two teflates and a norbonadienyl (NBD) ligand, namely [Pt­(NBD)​(OTeF_5_)_2_], was synthesized by reacting [PtCl_2_(NBD)] with AgOTeF_5_ (see Scheme 14).

Complexes with NHC and teflate ligands could be prepared for the late *d*-block metals nickel and gold. The nickel NHC complex was obtained by reaction of *trans*-[NiBr_2_(^*i*Pr^Im)_2_] (^*i*Pr^Im = 1,3-diisopropylimidazolium) with AgOTeF_5_ upon precipitation of AgBr (see Scheme 14). In contrast to the starting complex with the bromide ligands *trans* to each other, *cis*-[Ni­(^*i*Pr^Im)_2_(OTeF_5_)_2_] was formed and is a rare example of Ni­(II) NHC complexes with the two carbene ligands coordinated in *cis*-position to each other.^258,265^ Another relevant NHC complex with a teflate ligand is *trans*-[AuF_2_(OTeF_5_)​(SIMes)], which is formed in a reaction of [AuF_3_(SIMes)] with Me_3_SiOTeF_5_ upon elimination of Me_3_SiF (see eq 84). Here, due to the *trans* influence of the NHC ligand, the Au–F bond in *trans* position to the carbene is elongated and thus only this fluoride is replaced by a teflate ligand, which is therefore transferred in *trans* position to SIMes.^179^ For the case of *d*-block metal complexes containing only teflate and alkyl ligands only one example is known, i.e., HgMe­(OTeF_5_), which is selectively formed by reaction of HgMe_2_ and HOTeF_5_ (see eq 85). The addition of an excess of HOTeF_5_ or a reaction temperature up to 140 °C does not lead to Hg­(OTeF_5_)_2_, similarly to the formation of *cis*-[Os­(CO)_4_(OTeF_5_)­Me].^11^ Heteroleptic transition metal compounds are gathered in Table 11.


88 [AuF 3(SIMes)]→−TMSFTMSOTeF 5[AuF 2(OTeF 5)(SIMes)]

89 HgMe 2→−CH4HOTeF 5HgMe(OTeF 5)

**Table 11:** Overview of Heteroleptic Transition Metal Teflates

Element	Ox	Compound	Refs		Element	Ox	Compound	Refs
Ti	IV	TiCp_2_(OTeF_5_)_2_	^261^		Os	±0	[HOs(CO)_3_(PPh_3_)_2_]​[OTeF_5_]	^264^
		TiCl_4–*n*_(OTeF_5_)_*n*_ (*n* = 1–3)	^239^			I	[Os(PPh_3_)​(CO)_4_(OTeF_5_)]	^257^
						II	[OsMe(CO)_4_(OTeF_5_)]	^257^
Zr	IV	ZrCp_2_(OTeF_5_)_2_	^261^			VI	OsO(OTeF_5_)_4_	^142^
						VII	OsO(OTeF_5_)_5_	^250^
Hf	IV	HfCp_2_(OTeF_5_)_2_	^261^					
					Ni	II	[Ni(Hacac)_2_(OTeF_5_)_2_]	^258^
V	V	VO(OTeF_5_)_3_	^142^				[Ni(^iPr^Im)_2_(OTeF_5_)_2_]	^258^
		VO(OSeF_5_)_3_	^42^				[NEt_4_]_2_[Ni(Et_2_O)_2_(OTeF_5_)_4_]	^34^
							[NEt_4_]​[Ni(bpyMe_2_)​(OTeF_5_)_3_]	^34^
Cr	VI	CrO_2_(OTeF_5_)_2_	^142^				[Ni(bpy)_2_(OSF_5_)_2_]	^110^
		CrO_2_(OSeF_5_)_2_	^42^					
					Pd	II	[Pd(OTeF_5_)_2_(PhCN)_2_]	^255^
Mo	±0	[N(*n*Bu)_4_]​[Mo(CO)_5_(OTeF_5_)]	^262^					
	IV	MoCp_2_(OTeF_5_)_2_	^261^		Pt	II	[Pt(NBD)​(OTeF_5_)_2_]	^255^
	VI	MoOF_3_(OTeF_5_)	^247^				[Pt(OTeF_5_)_2_(PEt_3_)_2_]	^256^
		MoO(OTeF_5_)_4_	^142,240^					
					Cu	I	[Cu(MeCN)_4_]​[OSF_5_]	^110^
W	±0	[N(*n*Bu)_4_]​[W(CO)_5_(OTeF_5_)]	^262^				[Cu(bpy)_2_(OSF_5_)]	^110^
	IV	WCp_2_(OTeF_5_)_2_	^261^			II	[Cu(bpy)_2_(OSF_5_)_2_]	^110^
	VI	WF_6–*n*_(OTeF_5_)_*n*_ (*n* = 1–5)	^142,251^					
		WCl(OTeF_5_)_5_	^42^		Ag	I	[Ag(1,2,3-C_3_H_5_Cl_3_)​(OTeF_5_)]	^237^
		WO(OTeF_5_)_4_	^240^				[Ag(bpy(*t*Bu)_2_)_2_(OSF_5_)]	^110^
							[Ag(bpy)_2_(OSF_5_)]	^110^
Mn	I	[Mn(CO)_5_(OTeF_5_)]	^108,263^					
		[Mn(CO)_5_(OTeF_5_)​(THF)]	^108,263^		Au	III	[Au(OTeF_5_)_3_(OPPh_3_)]	^24^
		[Mn(CO)_5_(OSeF_5_)]	^108^				[NMe_4_]​[AuCl_3_(OTeF_5_)]	^24^
							[AuF_2_(OTeF_5_)​(SIMes)]	^179^
Re	I	[Re(CO)_5_(OTeF_5_)]	^108^					
		[Re(CO)_5_(OSeF_5_)]	^108^		Zn	II	[Zn(PhNO_2_)_3_(OTeF_5_)_2_]	^259^
	VI	ReO(OTeF_5_)_4_	^250^				[Zn(PhNO_2_)_2_(OTeF_5_)_2_]_2_	^259^
	VII	ReO(OTeF_5_)_5_	^250^					
		ReO_2_(OTeF_5_)_3_	^142,249^		Hg	II	HgMe(OTeF_5_)	^11^
		[NMe_4_]​[ReO_2_(OTeF_5_)_4_]	^249^				[Hg(OTeF_5_)_2_]·1.5XeF_2_]	^233^
							[Hg(OTeF_5_)_2_]·1.5KrF_2_]	^233^
Fe	±0	[HFe(CO)_3_(PPh_3_)_2_]​[OTeF_5_]	^264^				[Hg(OTeF_5_)_2_·NSF_3_]_∞_	^260^
	II	[Fe(Cp)​(CO)_2_(OTeF_5_)]	^262^				[Hg(OTeF_5_)_2_·2NSF_3_]_2_	^260^
	III	Fe(OEP)​(OTeF_5_)	^253^				[Hg_3_(OTeF_5_)_6_·4NSF_3_]	^260^
		Fe(TPP)​(OTeF_5_)	^253,254^				[Hg(OTeF_5_)​(NSOF_2_)·NSF_3_]_∞_	^260^
		[Fe(TPP)​(OTeF_5_)​(THF)]	^253,254^				[Hg_3_(OTeF_5_)_5_(NSOF_2_)·2NSF_3_]_2_	^260^
	
Ru	±0	[HRu(CO)_3_(PPh_3_)_2_]​[OTeF_5_]	^264^		U	VI	UF_6–*n*_(OTeF_5_)_*n*_ (*n* = 1–5)	^17^

### 11.3 *f*-Block Metal Teflates

Whereas no lanthanide teflate species are known thus far, uranium provides the only example of an isolated and characterized actinide teflate compound and was reported by Seppelt.^17,266^ The homoleptic neutral U­(OTeF_5_)_6_ is a very water-sensitive yellow crystalline solid, which melts at 160 °C with slow decomposition. In the solid state, it appears as discrete molecules with the central uranium atom octahedrally bonded to the six teflate ligands, which are also almost octahedral, leading to an overall spherically shaped molecule (see Figure 28),^266^ quite similar to Te­(OTeF_5_)_6_.^22^ The negligible intermolecular forces between the different molecules account for the volatility of U­(OTeF_5_)_6_, which can be sublimed at 60 °C despite its high molecular weight. A recent theoretical study assigned an oxidation state of +VI to the U center in this compound.^267^

**Figure 28:**
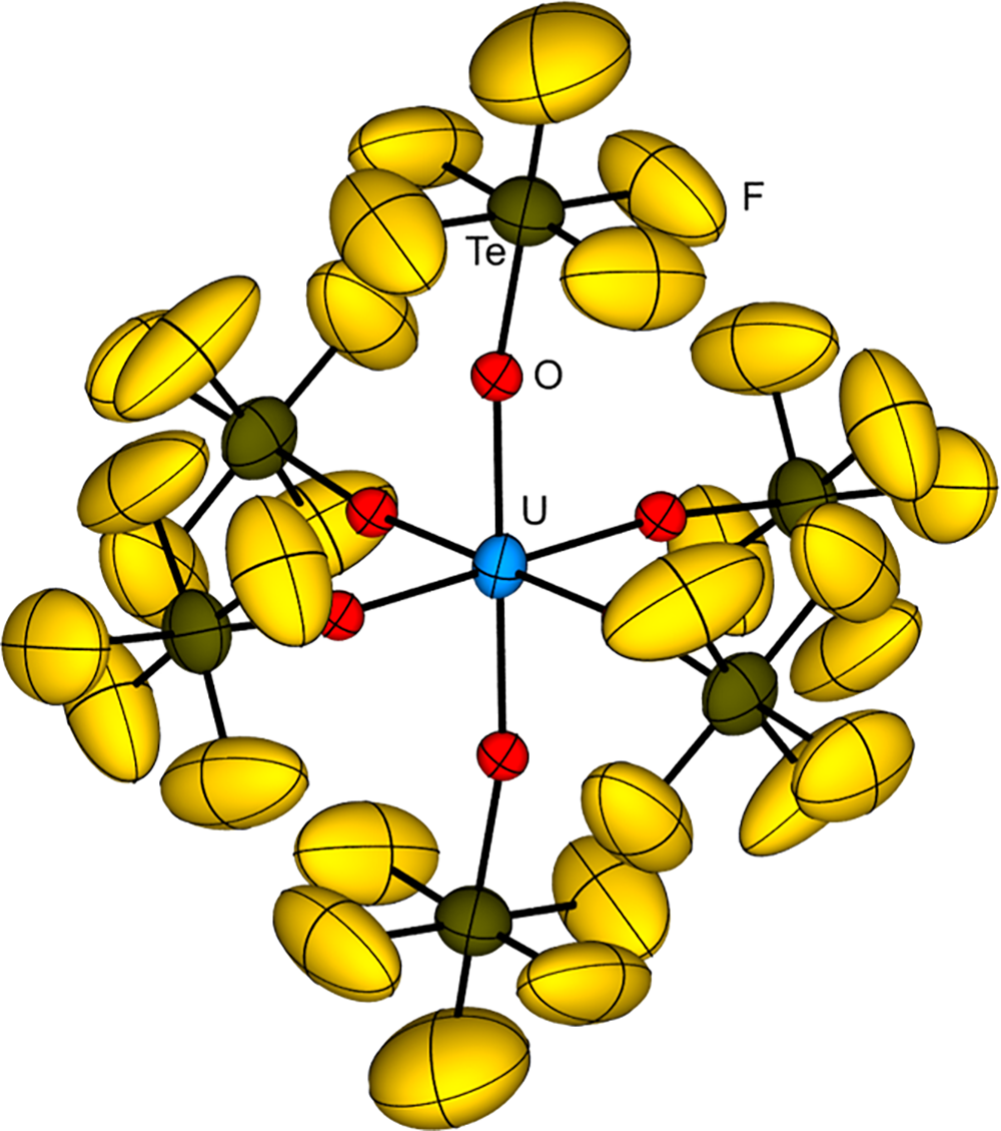
Molecular structure of U­(OTeF_5_)_6_ in the solid state.^266^ The O atoms are depicted isotropically, the thermal ellipsoids of the U, Te and F atoms are set at 25% probability.

Compound U­(OTeF_5_)_6_ can be prepared by using two different teflate transfer reagents, B­(OTeF_5_)_3_ and F_3_SiOTeF_5_, in both cases starting from UF_6_ and with yields higher than 95% (see eqs 86 and 86a).^17^ Both reactions take place stepwise and all the compounds with the formula F_*n*_U­(OTeF5)_6–*n*_ (*n* = 0–6), including all possible isomers, could be detected by NMR spectroscopy when an excess of UF_6_ was used. Nevertheless, none of these intermediates could be isolated from the obtained orange liquid mixture.

UF 6+2B(OTeF 5)3→−2BF 3U(OTeF 5)6 90

UF 6+6F 3SiOTeF 5→−6SiF 4U(OTeF 5)6 91

## 12 Conclusion

This Review underscores the exceptional properties and chemistry of pentafluoro­orthochalcogenate (OEF_5_, E = S, Se, Te) compounds in comparison with fluorine-containing species, highlighting their significance in contemporary and future chemical research with a strong focus on teflates. The combination of strong electron-withdrawing effects, substantial steric effects, and reduced lone pair back-bonding renders the OTeF_5_ group and its derivatives compelling substitutes for fluorine in various applications. Moreover, steric effects result in the formation of molecular structures that are more precise in comparison to fluorinated analogues, which often form oligomeric or polymeric structures. Evidently, these properties have facilitated the synthesis of a plethora of innovative compounds across the periodic table, ranging from new main group compounds to fundamental new transition metal complexes. This observation underscores the remarkable versatility of the teflate group and its derivatives, thereby highlighting their considerable potential in expanding the frontiers of modern chemistry in different areas such as Lewis or Brønsted acids, coordination chemistry, strong oxidizers and many more. Furthermore, teflate compounds are of interest not only as a result of their sometimes unique structures and properties but also due to their potential use for the preparation and stabilization of often fragile and very reactive species, which opens novel pathways in advanced synthesis and materials design.

Nevertheless, the field continues to encounter substantial challenges. The synthetic routes to teflate compounds often require costly and difficult-to-handle reagents, which restrict their extensive utilization in conventional chemical laboratories. Furthermore, some of these compounds exhibit sensitivity to hydrolysis, necessitating cautious handling that could impede their utilization in some applications. Consequently, there is a necessity for future research efforts to concentrate on the development of more efficient, straightforward-to-prepare, cost-effective, and environmentally friendly synthetic protocols to address these limitations.

The derivatization of the OTeF_5_ group, as reviewed in Section 2.6, represents an area of significant interest. Beyond improving synthetic protocols, there remains considerable room for exploring broader applications of pentafluoro­ortho­chalcogenate-based compounds (OEF_5_, E = S, Se, Te). These compounds demonstrate considerable potential in a number of intriguing fields of chemistry, including catalysis, the study of weakly coordinating anions, and the production of functional materials, as well as the stabilization of otherwise elusive chemical species. The OSF_5_ group in particular could be of importance in the future as a promising PFAS (per- and polyfluoroalkyl substances) substituent, e.g. in pharmaceuticals or agrochemicals.

Overall, the pentafluoro­orthotellurate group and its derivatives are of significant interest within the field of chemical science, yet their potential remains largely untapped. With ongoing innovation in their synthesis, characterization, and application, these compounds have the potential to play crucial roles in solving emerging challenges and driving discoveries in many fields of chemistry. This comprehensive review not only summarizes the current state of knowledge in the field but also serves as a call to action for further exploration of this promising class of compounds.

## Abbreviations

*a*HF: anhydrous hydrogen fluoride
acac: acetylacetonate
*ax*: axial
bp: boiling point
bpy: 2,2′-bipyridine
bpyMe_2_: 6,6′-dimethyl-2,2′-bipyridine
bpy­(*t*Bu)_2_: 4,4′-di-*tert*-butyl-2,2′-bipyridine
bz: benzene
Cp: cyclopentadienyl
DBDHM: 1,3-dibromo-5,5-dimethylhydantoin
DCE: 1,2-dichloroethane
DCM: dichloromethane
EDF: electron distribution function
*eq*: equatorial
ESP: electrostatic potential
Et: ethyl
F-113: 1,1,2-trichloro-1,2,2-trifluoroethane, Freon-113
F-114: 1,2-dichloro-1,1,2,2-tetrafluoroethane, Freon-114
FIA: fluoride ion affinity
HPLC: high-performance liquid chromatography
*i*Bu: isobutyl
*i*Pr: isopropyl
^*i*Pr^Im: 1,3-diisopropylimidazolium
IQA: interacting quantum atoms
IR: infrared
ITC: isothermal titration calorimetry
m-CBA: 3-chlorobenzoic acid
m-CPBA: 3-chloroperbenzoic acid
Me: methyl
Mes: mesitylene
mp: melting point
NBD: norbonadienyl
*n*Bu: *n*-butyl
Ng: noble gas
NHC: N-heterocyclic carbene
NMR: nuclear magnetic resonance
NPA: natural population analysis
*n*Pr: *n*-propyl
OEP: 2,3,7,8,12,13,17,18-octaethylporphyrinate
OTf: triflate
PFAS: per- and polyfluoroalkyl substances
Ph: phenyl
PNP: bis­(triphenylphosphine)­iminium
py: pyridine
QTAIM: quantum theory of atoms in molecules
r.t.: room temperature
*s*Bu: *sec*-butyl
SIMes: 1,3-bis­(2,4,6-trimethylphenyl)-4,5-dihydroimid­azol-2-ylidene
solv: solvent
*t*Bu: *tert*-butyl
TCICA: trichloroisocyanuric acid
THF: tetrahydrofuran
THP: tetrahydropyran
tol: toluene
TPP: 5,10,15,20-tetraphenylporphyrinate
WCA: weakly coordinating anion
XRD: X-ray diffraction

## References

[1] Seppelt K. (1982). Stabilization
of Unusual Oxidation and Coordination States by the Ligands OSF_5_, OSeF_5_, and OTeF_5_. Angew. Chem. Int. Ed..

[2] Engelbrecht A., Sladky F. (1964). Pentafluoro-orthotelluric
Acid, HOTeF_5_. Angew. Chem. Int. Ed..

[3] Engelbrecht A., Sladky F. (1965). Zur Chemie der Tellur-Fluor-Verbindungen,
1. Mitt.:
Die Produkte der Umsetzung von Bariumtellurat mit Fluorsulfonsäure,
neue Verbindungen mit der F_5_Te-Gruppe. Monatsh. Chem..

[4] Engelbrecht A., Loreck W., Nehoda W. (1968). Zur Chemie der Tellur-Fluor-Verbindungen,
II. Chemische Eigenschaften und physikalische Konstanten einiger,
die F_5_Te-Gruppe enthaltende Verbindungen. Z. Anorg. Allg. Chem..

[5] Seppelt K., Nothe D. (1973). Stability of Xenon­(II)
Compounds. The Pentafluorooxyselenium and
Pentafluorooxytellurium Radicals. Bis­(pentafluorotellurium) Peroxide
and Chlorine Pentafluoroorhtotellurate. Inorg.
Chem..

[6] Schack C. J., Wilson W. W., Christe K. O. (1983). Synthesis and Characterization of
TeF_5_OF. Inorg. Chem..

[7] Strauss S. H., Abney K. D., Anderson O. P. (1986). An Unusual
Hydrogen Bond between
Oxygen Atoms: Preparation and Characterization of [N­(*n*-Bu)_4_
^+^]​[H­(OTeF_5_)_2_
^–^]. Inorg. Chem..

[8] Porcham W., Engelbrecht A. (1971). Bestimmung
der Säurestärke der Pentafluoro-orthotellursäure,
F_5_TeOH. Monatsh. Chem..

[9] Sladky F., Kropshofer H., Leitzke O. (1973). Boron tris­(pentafluoro-orthotellurate),
B­(OTeF_5_)_3_. Preparation and Lewis acidity. J. Chem. Soc., Chem. Commun..

[10] Mayer E., Sladky F. (1975). Infrared and Raman Spectra of the TeF_5_O^–^ Anion and Evidence for Contact-Ion-Pair Formation
in the TeF_5_OAg-CH_3_CN System. Normal-Coordinate
Analysis of the TeF_5_O^–^ and SeF_5_O^–^ Ions. Inorg. Chem..

[11] Sladky F., Kropshofer H., Leitzke O., Peringer P. (1976). Synthesis and characterization
of compounds containing the F_5_TeO^–^ anion. J. Inorg. Nucl. Chem..

[12] Sladky F. (1970). Zur Chemie
des Xenons, 1. Mitt.: Xenon­(II)-bis­(pentafluoro-orthotellurat), Xe­(OTeF_5_)_2_. Monatsh. Chem..

[13] Sladky F. (1970). Zur Chemie
des Xenons, 2. Mitt.: Xenon­(II)-fluorid-pentafluoro-orthotellurat,
FXeOTeF_5_ und das System XeF_2_-CF_3_COOH. Monatsh. Chem..

[14] Sladky F. (1970). Zur Chemie
des Xenons, 3. Mitt.: Xenon­(II)-fluorid-pentafluoro-orthotellurat
als Fluoridionen-Donor: [XeOTeF_5_]^+^[AsF_6_]^−^. Monatsh. Chem..

[15] Lentz D., Seppelt K. (1983). OTeF_5_-Verbindungen
von P, As und Sb. Z. Anorg. Allg. Chem..

[16] Lentz D., Seppelt K. (1979). Xe­(OTeF_5_)_6_, A Deep-Colored Noble
Gas Compound, and O=Xe­(OTeF_5_)_4_ - The Existence
of Kr­(OTeF_5_)_2_. Angew.
Chem. Int. Ed..

[17] Seppelt K. (1976). Covalent Compounds
of Uranium of the Type F_x_U­(OTeF_5_)_6‑x_. Chem. Ber..

[18] Seppelt K. (1977). Neue -OSeF_5_- und -OTeF_5_-Verbindungen. Chem. Ber..

[19] Sladky F., Kropshofer H. (1973). Pentafluoro-orthotellurates of Silicon, Germanium,
and Tin. J. Chem. Soc., Chem. Commun..

[20] Lentz D., Seppelt K. (1978). Xenon-tetrakis­(pentafluoro­orthotellurat),
Xe­(OTeF_5_)_4_. Angew. Chem..

[21] Lentz D., Seppelt K. (1980). Pentafluorotellurate­(VI) des fünfwertigen Iod;
die Elektronegativität der Gruppen OTeF_5_ und OSeF_5_. Z. Anorg. Allg. Chem..

[22] Lentz D., Pritzkow H., Seppelt K. (1978). Novel Tellurium
Oxide Fluorides: *cis*- and *trans*-F_4_Te­(OTeF_5_)_2_, *cis*- and *trans*-F_2_Te­(OTeF_5_)_4_, FTe­(OTeF_5_)_5_, and Te­(OTeF_5_)_6_. Inorg. Chem..

[23] Seppelt K. (1973). Neue Derivate
der Pentafluoro­orthoselensäure, Halogenderivate der Pentafluoro­orthotellursäure. Chem. Ber..

[24] Winter M., Peshkur N., Ellwanger M. A., Pérez-Bitrián A., Voßnacker P., Steinhauer S., Riedel S. (2023). Gold Teflates Revisited:
From the Lewis Superacid [Au­(OTeF_5_)_3_] to the
Anion [Au­(OTeF_5_)_4_]^−^. Chem. Eur. J..

[25] Drews T., Seppelt K. (1991). Fe­(OTeF_5_)_3_, Darstellung, Struktur
und Reaktivität. Z. Anorg. Allg. Chem..

[26] Fischer L., Hoffmann K. F., Riedel S. (2024). A Rare Example of a Gallium-based
Lewis Superacid: Synthesis and Reactivity of Ga­(OTeF_5_)_3_. Chem. Eur. J..

[27] Fundel, S. Neue Verbindungen mit OTeF_5_- und NHTeF_5_-Liganden. Dissertation, Freien Universität Berlin, 2003.

[28] Lentz D., Pritzkow H., Seppelt K. (1977). The Te-O-Fe
System: Te­(OTeF_5_)_6_. Angew.
Chem. Int. Ed..

[29] Engelbrecht, A. ; Sladky, F. Selenium and Tellurium Fluorides. Advances in Inorganic Chemistry and Radiochemistry; Elsevier, 1981; pp 189–223. 10.1016/S0065-2792(08)60328-3.

[30] Sladky F., Kropshofer H. (1972). Ester der pentafluoro-orthotellur­säure:
F_5_TeOCH_3_, F_5_TeOC_2_H_5_, F_5_TeOCH_2_C­(O)­OC_2_H_5_. Inorg. Nucl. Chem. Lett..

[31] Lentz D., Seppelt K. (1978). Extremely High Electronegativities. Angew. Chem. Int. Ed..

[32] Birchall T., Myers R. D., de Waard H., Schrobilgen G. J. (1982). Multinuclear
Nuclear Magnetic Resonance and Mössbauer Study of OTeF_5_ Derivatives of Tellurium, Iodine, and Xenon. Inorg. Chem..

[33] Barrena-Espés D., Martín
Pendás Á., Riedel S., Pérez-Bitrián A., Munárriz J. (2025). Pentafluoro­orthotellurate Uncovered: Theoretical
Perspectives on an Extremely Electronegative Group. Inorg. Chem..

[34] Pérez-Bitrián A., Hoffmann K. F., Krause K. B., Thiele G., Limberg C., Riedel S. (2022). Unravelling the Role of the Pentafluoro­orthotellurate
Group as a Ligand in Nickel Chemistry. Chem.
Eur. J..

[35] Pérez-Bitrián A., Munárriz J., Krause K. B., Schlögl J., Hoffmann K. F., Sturm J. S., Hadi A. N., Teutloff C., Wiesner A., Limberg C., Riedel S. (2024). Questing for homoleptic
mononuclear manganese complexes with monodentate O-donor ligands. Chem. Sci..

[36] Pérez-Bitrián A., Munárriz J., Sturm J. S., Wegener D., Krause K. B., Wiesner A., Limberg C., Riedel S. (2023). Further Perspectives
on the Teflate versus Fluoride Analogy: The Case of a Co­(II) Pentafluoro­orthotellurate
Complex. Inorg. Chem..

[37] Yeh S. K., Wu S. Y., Lee C. S., Wang Y. (1993). Electron-Density Distribution
in a Crystal of Potassium Tetrafluoronickelate, K_2_NiF_4_. Acta Crystallogr. B Struct. Sci..

[38] Keve E. T., Abrahams S. C., Bernstein J. L. (1969). Crystal Structure of Pyroelectric
Paramagnetic Barium Manganese Fluoride, BaMnF_4_. J. Chem. Phys..

[39] Bukovec P., Hoppe R. (1984). Zur Kenntnis von BaMnF_5_: Eine Jahn-Teller-verzerrte Variante
von BaGaF_5_. Z. Anorg. Allg. Chem..

[40] Huppmann P., Hartl H., Seppelt K. (1985). Synthese und Struktur von Au­(OTeF_5_)_3_. Z. Anorg. Allg. Chem..

[41] Einstein F. W. B., Rao P. R., Trotter J., Bartlett N. (1967). The crystal structure
of gold trifluoride. J. Chem. Soc. A.

[42] Seppelt K. (1975). Übergangsmetall-pentafluoro­orthochalcogenate. Chem. Ber..

[43] Mercier H. P. A., Moran M. D., Sanders J. C. P., Schrobilgen G. J., Suontamo R. J. (2005). Synthesis, Structural
Characterization, and Computational
Study of the Strong Oxidant Salt [XeOTeF_5_]​[Sb­(OTeF_5_)_6_]·SO_2_ClF. Inorg. Chem..

[44] Mercier H. P. A., Sanders J. C. P., Schrobilgen G. J. (1994). Hexakis­(pentafluoro­oxotellurato)­pnictate­(V)
Anions, M­(OTeF_5_)_6_
^–^ (M = As,
Sb, Bi): A Series of Very Weakly Coordinating Anions. J. Am. Chem. Soc..

[45] Tötsch W., Sladky F. (1983). Die Hydrolyse von TeF_6_. Z. Naturforsch. B.

[46] Muetterties E. L. (1957). Fluorotellurates. J. Am. Chem. Soc..

[47] Selig H., Sarig S., Abramowitz S. (1974). Alkali fluorotellurates­(VI). Inorg. Chem..

[48] Mahjoub A.-R., Seppelt K. (1991). Preparation and Structure
of the IOF_6_
^–^ and TeF_7_
^–^ Anions. J. Chem. Soc., Chem.
Commun..

[49] Mahjoub A.-R., Drews T., Seppelt K. (1992). The Pentagonal Bipyramid as Fundamental
Structure for Compounds with the Coordination Number Seven. Angew. Chem. Int. Ed..

[50] Engesser T. A., Lichtenthaler M. R., Schleep M., Krossing I. (2016). Reactive p-block cations
stabilized by weakly coordinating anions. Chem.
Soc. Rev..

[51] Hoffmann K. F., Wiesner A., Subat N., Steinhauer S., Riedel S. (2018). Salts of the Weakly Coordinating Anion [Al­(OTeF_5_)_4_]^−^ containing Reactive Counterions. Z. Anorg. Allg. Chem..

[52] Erdmann P., Leitner J., Schwarz J., Greb L. (2020). An Extensive Set of
Accurate Fluoride Ion Affinities for p-Block Element Lewis Acids and
Basic Design Principles for Strong Fluoride Ion Acceptors. ChemPhysChem.

[53] Böhrer H., Trapp N., Himmel D., Schleep M., Krossing I. (2015). From unsuccessful
H_2_-activation with FLPs containing B­(Ohfip)_3_ to a systematic evaluation of the Lewis acidity of 33 Lewis acids
based on fluoride, chloride, hydride and methyl ion affinities. Dalton Trans..

[54] Greb L. (2018). Lewis Superacids:
Classifications, Candidates, and Applications. Chem. Eur. J..

[55] Wiesner A., Gries T. W., Steinhauer S., Beckers H., Riedel S. (2017). Superacids
Based on Pentafluoro­orthotellurate Derivatives of Aluminum. Angew. Chem. Int. Ed..

[56] Hoffmann K. F., Wiesner A., Steinhauer S., Riedel S. (2022). Insights on the Lewis
Superacid Al­(OTeF_5_)_3_: Solvent Adducts, Characterization
and Properties. Chem. Eur. J..

[57] Collins M. J., Schrobilgen G. J. (1985). Study of
the OTeF_5_ Donor Properties of Te­(OTeF_5_)_4_ by ^75^As and ^125^Te NMR
Spectroscopy. Preparation and Characterization of the [TeFe_
*x*
_(OTeF_5_)_3‑*x*
_]^+^ Cations, TeF_
*x*
_(OTeF_5_)_4‑*x*
_, As­(OTeF_5_)_5_ and [As­(OTeF_5_)_6_]^−^. Inorg. Chem..

[58] Gerken M., Kolb P., Wegner A., Mercier H. P., Borrmann H., Dixon D. A., Schrobilgen G. J. (2000). Tetrachloro-
and Tetrabromoarsonium­(V)
Cations: Raman and ^75^As, ^19^F NMR Spectroscopic
Characterization and X-ray Crystal Structures of [AsCl_4_]​[As­(OTeF_5_)_6_] and [AsBr_4_]​[AsF­(OTeF_5_)_5_]. Inorg. Chem..

[59] Strauss S. H. (1993). The Search
for Larger and More Weakly Coordinating Anions. Chem. Rev..

[60] Krossing I., Raabe I. (2004). Noncoordinating Anions-Fact or Fiction? A Survey of Likely Candidates. Angew. Chem. Int. Ed..

[61] Finze M., Bernhardt E., Willner H. (2007). Trifluoromethylboranes and -Borates:
New Synthetic Strategies and Applications. Angew.
Chem. Int. Ed..

[62] Riddlestone I. M., Kraft A., Schaefer J., Krossing I. (2018). Taming the Cationic
Beast: Novel Developments in the Synthesis and Application of Weakly
Coordinating Anions. Angew. Chem. Int. Ed..

[63] Kropshofer H., Leitzke O., Peringer P., Sladky F. (1981). Darstellung und Eigenschaften
von B­(OTeF_5_)_3_, Cs­[B­(OTeF_5_)_4_] und B­(OTeF_5_)_3_ · CH_3_CN. Chem. Ber..

[64] Van
Seggen D. M., Hurlburt P. K., Anderson O. P., Strauss S. H. (1992). Larger
and More Weakly Coordinating Anions: Nb­(OTeF_5_)_6_
^–^ and Ti­(OTeF_5_)_6_
^2–^. J. Am. Chem. Soc..

[65] Moock K., Seppelt K. (1988). Darstellung und elektrochemisches
Verhalten von [Nb­(OTeF_5_)_6_]^−^ und [Ta­(OTeF_5_)_6_]^−^-Komplexen. Z. Anorg. Allg. Chem..

[66] Wiesner A., Fischer L., Steinhauer S., Beckers H., Riedel S. (2019). Oxygen-Bridged
Ga_2_(Et)_3_(OTeF_5_)_3_ and the
Weakly Coordinating Anions [Ga­(Et)​(OTeF_5_)_3_]^−^ and [Ga­(OTeF_5_)_4_]^−^. Chem. Eur. J..

[67] Mercier H. P. A., Moran M. D., Schrobilgen G. J., Steinberg C., Suontamo R. J. (2004). The Syntheses of Carbocations by
Use of the Noble-Gas
Oxidant, [XeOTeF_5_]​[Sb­(OTeF_5_)_6_]: The Syntheses and Characterization of the CX_3_
^+^ (X = Cl, Br, OTeF_5_) and CBr­(OTeF_5_)_2_
^+^ Cations and Theoretical Studies of CX_3_
^+^ and BX_3_ (X = F, Cl, Br, I, OTeF_5_). J. Am. Chem. Soc..

[68] Mercier, H. P. A. ; Moran, M. D. ; Schrobilgen, G. J. Syntheses of the CFY_2_ ^+^ (Y = Cl, Br) and CX_3_ ^+^ (X = Cl, Br, OTeF_5_) Cations Employing the Noble-Gas Oxidant, XeOTeF_5_ ^+^Sb­(OTeF_5_)_6_ ^–^ . In Recent Developments in Carbocation and Onium Ion Chemistry; Laali, K. K. , Ed.; ACS Symposium Series 965; American Chemical Society, 2007; pp 394–427. 10.1021/bk-2007-0965.ch019.

[69] Ulferts P., Seppelt K. (2004). Formation and Crystal Structure of [(F_5_TeOXe)^+^·SO_2_ClF]​[Sb­(OTeF_5_)_6_
^–^]. Z. Anorg. Allg.
Chem..

[70] Toraman A. N., Fischer L., Pérez-Bitrián A., Wiesner A., Hoffmann K. F., Riedel S. (2024). [Xe­(OTeF_5_)​(py^F^)]^+^: A Strong Oxidizing Xenonium­(II)
Teflate Cation with N-Donor Bases. Chem. Commun..

[71] Hoffmann K.
F., Battke D., Golz P., Rupf S. M., Malischewski M., Riedel S. (2022). The Tris­(pentafluorophenyl)­methylium Cation: Isolation
and Reactivity. Angew. Chem. Int. Ed..

[72] Wiesner A., Steinhauer S., Beckers H., Müller C., Riedel S. (2018). [P_4_H]^+^[Al­(OTeF_5_)_4_]^−^: protonation
of white phosphorus with
the Brønsted superacid H­[Al­(OTeF_5_)_4_]_(solv)_. Chem. Sci..

[73] Hämmerling S., Thiele G., Steinhauer S., Beckers H., Müller C., Riedel S. (2019). A Very Strong Methylation
Agent: [Me_2_Cl]​[Al­(OTeF_5_)_4_]. Angew. Chem. Int. Ed..

[74] Kotsyuda S., Toraman A. N., Voßnacker P., Ellwanger M. A., Steinhauer S., Müller C., Riedel S. (2023). Noncovalent Interactions
in Halogenated Pyridinium Salts of the Weakly Coordinating Anion [Al­(OTeF_5_)_4_]^−^. Chem.
Eur. J..

[75] Fischer L., Lee M. H., Kim I., Wiesner A., Hoffmann K. F., Riedel S. (2024). Fluorinated Dialkyl Chloronium Salts: Synthesis and
Reactivity for Fluoroalkylation and Hydride Abstraction. Angew. Chem. Int. Ed..

[76] Reisinger A., Trapp N., Krossing I., Altmannshofer S., Herz V., Presnitz M., Scherer W. (2007). Homoleptic Silver­(I)
Acetylene Complexes. Angew. Chem. Int. Ed..

[77] Reisinger A., Trapp N., Knapp C., Himmel D., Breher F., Rüegger H., Krossing I. (2009). Silver-Ethene Complexes [Ag­(η^2^-C_2_H_4_)_
*n*
_]​[Al­(OR^F^)_4_] with *n* = 1, 2, 3 (R^F^ = Fluorine-Substituted Group). Chem. Eur.
J..

[78] Bihlmeier A., Gonsior M., Raabe I., Trapp N., Krossing I. (2004). From Weakly
Coordinating to Non-Coordinating Anions? A Simple Preparation of the
Silver Salt of the Least Coordinating Anion and its Application to
Determine the Ground State Structure of the Ag­(η^2^-P_4_)_2_
^+^ Cation. Chem. Eur. J..

[79] Sellin M., Krossing I. (2023). Homoleptic Transition
Metal Carbonyl Cations: Synthetic
Approaches, Characterization and Follow-Up Chemistry. Acc. Chem. Res..

[80] Neese F. (2022). Software update:
The ORCA program system—Version 5.0. WIREs Comput. Mol. Sci..

[81] Chemcraft - graphical software for visualization of quantum chemistry computations, Version 1.8. https://www.chemcraftprog.com/.

[82] Elinburg J. K., Doerrer L. H. (2020). Synthesis, structure,
and electronic properties of
late first-row transition metal complexes of fluorinated alkoxides
and aryloxides. Polyhedron.

[83] Blanco M. A., Martín Pendás A., Francisco E. (2005). Interacting
Quantum Atoms: A Correlated Energy Decomposition Scheme Based on the
Quantum Theory of Atoms in Molecules. J. Chem.
Theory Comput..

[84] Mitra G., Cady G. H. (1959). Preparation and Properties of Pentafluoroselenium Hypofluorite
(F_5_SeOF) and Bis-(pentafluoroselenium) Peroxide (F_5_SeOOSeF_5_). J. Am. Chem. Soc..

[85] Seppelt K. (1973). Halogenderivate
der Pentafluoro­orthoselensäure. Chem. Ber..

[86] Huppmann P., Lentz D., Seppelt K. (1981). Pentafluoroselenate­(VI)
des Kohlenstoffs,
die Elektronegativität der Gruppe −OSeF_5_. Z. Anorg. Allg. Chem..

[87] Damerius R., Huppmann P., Lentz D., Seppelt K. (1984). Ligand Properties of
the −OSeF_5_ and −OTeF_5_ Groups in
Pseudo-trigonal-bipyramidal Molecules. J. Chem.
Soc., Dalton Trans..

[88] Seppelt K. (1972). Pentafluoro-orthoselenate. Chem. Ber..

[89] Seppelt K., Oberhammer H. (1985). Pentafluoroselenium
Cyanate, F_5_Se-O-C≡N. Inorg.
Chem..

[90] Seppelt K. (1972). Pentafluoro-orthoselensäure-disulfurylfluorid
und Pentafluoro-orthoselensäure-trifluoracetat. Chem. Ber..

[91] Seppelt K., Desmarteau D. D. (1977). Die Pentafluoro-orthoschwefelsäure
HOSF_5_ und ihre höheren Homologen. Z. Anorg. Allg. Chem..

[92] Seppelt, K. ; Desmarteau, D. D. Hydrogen Pentafluoro­oxoselenate­(VI). In Inorganic Syntheses; Shreeve, J. M. , Ed.; John Wiley & Sons, 1986; pp 38–41. 10.1002/9780470132517.ch10.

[93] Seppelt K. (1972). Pentafluoro­orthoselenic
Acid. Angew. Chem. Int. Ed..

[94] Seppelt K. (1974). Fluorochalkogenate
und deren Pyrolyseprodukte: SeOF_4_, Se_2_O_2_F_8_, TeO_2_F_4_
^2–^ und Te_2_O_2_F_8_. Z. Anorg. Allg. Chem..

[95] Seppelt K. (1972). Xenon-bis­(pentafluoro­orthoselenat)
und Xenon-fluorid-pentafluoro­orthoselenat. Angew. Chem..

[96] Holleman, A. F. ; Wiberg, E. Lehrbuch der anorganischen Chemie; de Gruyter, 2008; Vol. 102, p 628.

[97] Oberhammer H., Seppelt K. (1979). Molecular structures of Di-μ-oxy-diselenium Octafluoride,
Se_2_O_2_F_8_, and Di-μ-oxy-ditellurium
Octafluoride, Te_2_O_2_F_8_. Inorg. Chem..

[98] Smith J. E., Cady G. H. (1970). Reactions of Fluoroxypentafluoro­selenium. Inorg. Chem..

[99] Reichert W. L., Cady G. H. (1973). Reactions of Bis­(pentafluoroselenium)
Peroxide. Synthesis
of Pentafluoroselenium Fluorosulfate and of Bis­(pentafluoroselenium)
Oxide. Inorg. Chem..

[100] Oberhammer H., Seppelt K. (1978). Molecular Structure
of F_5_SOSF_5_, F_5_SeOSeF_5_ and
F_5_TeOTeF_5_: d-Orbital Participation in Bonds
between Main
Group Elements. Angew. Chem. Int. Ed..

[101] Oberhammer H., Seppelt K. (1978). Electron Diffraction Study of Bis­(pentafluorosulfur),
Bis­(pentafluoroselenium), and Bis­(pentafluorotellurium) Oxides. Inorg. Chem..

[102] Kleemann G., Seppelt K. (1983). Methylenschwefeltetrafluorid,
CH_2_=SF_4_, Darstellung, Struktur und Chemie. Chem. Ber..

[103] Seppelt K. (1972). Xenon Bis­(pentafluoro­orthoselenate) and Xenon
Fluoride Pentafluoro­orthoselenate. Angew.
Chem. Int. Ed..

[104] Seppelt, K. ; Lentz, D. ; Klöter, G. ; Schack, C. J. Selenium Tetrafluoride, Selenium Difluoride Oxide (Seleninyl Fluoride), and Xenon Bis­[Pentafluoro­oxoselenate­(VI)]. In Inorganic Syntheses; Shreeve, J. M. , Ed.; John Wiley & Sons, 1986; pp 27–31. 10.1002/9780470132555.ch9.

[105] Templeton L. K., Templeton D. H., Seppelt K., Bartlett N. (1976). Crystal and
Molecular Structure of Xenon Bis­(oxopentafluoroselenate­(VI)), Xe­(OSeF_5_)_2_. Inorg. Chem..

[106] Fir B. A., Mercier H. P. A., Sanders J. C. P., Dixon D. A., Schrobilgen G. J. (2001). Structural
and theoretical studies of Xe­(OChF_5_)_2_ and [XeOChF_5_]​[AsF_6_] (Ch
= Se, Te). J. Fluorine Chem..

[107] Seppelt K., Rupp H. H. (1974). ^129^Xe-Kernresonanzuntersuchungen
von Xenonverbindungen. II. Xenon­(II)-Derivate. Z. Anorg. Allg. Chem..

[108] Crossman M. C., Hope E. G., Wootton L. J. (1998). Reactions
of [M_2_(CO)_10_] (M = Mn or Re) with xenon bis­[pentafluoro­oxo-tellurate­(VI)
and -selenate­(VI)]. J. Chem. Soc., Dalton Trans..

[109] Sturm J. S., Millanvois A., Bahri C., Golz P., Limberg N., Wiesner A., Riedel S. (2024). Streamlining Thionyl
Tetrafluoride (SOF_4_) and Pentafluoro­oxosulfate [OSF_5_]^−^ Anions Syntheses. Chem. Eur. J..

[110] Haupt A., Duvinage D., Lork E., Ponomarenko M., Röschenthaler G.-V. (2021). A Versatile Silver­(I) Pentafluoro­oxosulfate
Reagent for the Synthesis of OSF_5_ Compounds. Angew. Chem. Int. Ed..

[newref111] Millanvois A., Bahri C., Riedel S. (2025). Thionyl Tetrafluoride. EROS.

[111] Ruff J. K., Lustig M. (1964). Sulfur Oxyfluoride
Derivatives. I. Inorg. Chem..

[112] Schack C. J., Wilson R. D., Muirhead J. S., Cohz S. N. (1969). Chloroxysulfur
Pentafluoride. J. Am. Chem. Soc..

[113] Anderson L. R., Young D. E., Gould D. E., Juurik-Hogan R., Neuchterlein D., Fox W. B. (1970). Perhaloalkyl Hypochlorites and Pentafluorosulfur
Hypochlorite. IV. Reactions with Olefins. J.
Org. Chem..

[114] Place R. D., Williamson S. M. (1968). The Preparation and Characterization
of Some New Pentafluorosulfuroxyalkanes and -alkenes. J. Am. Chem. Soc..

[115] Case J. R., Pass G. (1964). 183. Pentafluorosulphuroxy-derivatives
of hexafluoropropene. J. Chem. Soc..

[116] Schack C. J., Wilson R. D., Christe K. O. (1989). Synthesis
of SF_5_O- substituted fluorocarbons. J. Fluorine
Chem..

[117] Case J. R., Price R., Ray N. H., Roberts H. L., Wright J. (1962). Preparation and Properties of Some Pentafluorosulphuroxyaryl
Compounds, ArO·SF_5_. J. Chem.
Soc..

[118] Merrill C. I., Cady G. H. (1961). Bis-(pentafluorosulfur)
Peroxide. J. Am. Chem. Soc..

[119] Hwang S. H., Naik K., Desmarteau D. D. (1993). Improved
Methods for the Synthesis of SF_5_OSF_5_ and SF_5_O_2_SF_5_. Inorg.
Chem..

[120] Zogu A., Ullah K., Spanopoulos S., Ismalaj E., De Borggraeve W. M., Demaerel J. (2024). Perfluoro­oxosulfate
Salts as SOF_4_-Gas-Free Precursors to Multidimensional SuFEx
Electrophiles. Angew. Chem. Int. Ed..

[121] Shou J.-Y., Qing F.-L. (2025). Pentafluorosulfanoxylation
of Hypervalent
Chlorines and Bromines for Access to Pentafluoro­(biaryloxy)-*λ*
_6_-sulfanes. Org.
Lett..

[122] Kraemer Y., Stephens A. M., Buldt J. A., Young A., McLoughlin C. P., Fettinger J. C., Holder L. M., Pitts C. R. (2025). Strain-release
trifluoromethoxylation and pentafluorosulfanoxylation of [1.1.0]­bicyclobutanes:
expanded access to fluorinated cyclobutane hybrid bioisosteres. Chem. Commun..

[newref123] Millanvois A., Bahri C., Drews T., Steinhauer S., Riedel S. (2025). Assessing Fluorosulfonyl Pentafluorooxosulfate
(FSO_2_–OSF_5_) Reservoir Capacity: Selective
SOF_4_, SO_2_F_2_, and [OSF_5_]^–^ Anion Release. Angew.
Chem. Int. Ed..

[123] Dudley F. B., Cady G. H., Eggers D. F. (1956). Pentafluorosulfur
Hypofluorite and Thionyl Tetrafluoride. J. Am.
Chem. Soc..

[124] Seppelt K. (1976). Pentafluoro­orthosulfuric Acid, HOSF_5_. Angew. Chem. Int. Ed..

[125] Lustig M., Ruff J. K. (1967). Nonmetal oxy- and
thiofluoride salts. Inorg. Chem..

[126] Gage J. C. (1970). The subacute
inhalation toxicity of 109 industrial
chemicals. Br. J. industr. Med..

[127] Jansen A. F., Alam K., Blackburn B. J. (1989). Reactions
of phenyltellurium­(VI) fluorides with alcohols, amines and silicon
compounds. J. Fluorine Chem..

[128] Lermontov S. A., Zavorin S. I., Bakhtin I. V., Pushin A. N., Zefirov N. S., Stang P. J. (1998). Fluorination of
olefins with PhSeF_3_, PhSeF_5_ and PhTeF_5_. J. Fluorine Chem..

[129] Lermontov S. A., Zavorin S. I., Bakhtin I. V., Zefirov N. S., Stang P. J. (1995). Fluorinating Properties of PhTeF_5_ and PhSeF_5_, towards C = C Bond. Phosphorus Sulfur
Silicon Relat. Elem..

[130] Ou X., Janzen A. F. (2000). Oxidative fluorination
of S, Se and Te compounds. J. Fluorine Chem..

[131] Klein G., Naumann D. (1985). Tieftemperatur-flüssigphase-fluorierung
von (C_6_F_5_)_2_Te: Bildung von (C_6_F_5_)_2_TeF_2_, (C_6_F_5_)_2_TeF_4_ und (C_6_F_11_)_2_TeF_4_. J. Fluorine Chem..

[132] Bornemann D., Pitts C. R., Ziegler C. J., Pietrasiak E., Trapp N., Kueng S., Santschi N., Togni A. (2019). Pentafluoro­(aryl)-λ^6^-tellanes and Tetrafluoro­(aryl)​(trifluoromethyl)-λ^6^-tellanes: From SF_5_ to the TeF_5_ and
TeF_4_CF_3_ Groups. Angew.
Chem. Int. Ed..

[133] Kraemer Y., Bergman E. N., Togni A., Pitts C. R. (2022). Oxidative
Fluorination of Heteroatoms Enabled by Trichloroisocyanuric Acid and
Potassium Fluoride. Angew. Chem. Int. Ed..

[134] Wegener D., Hoffmann K. F., Pérez-Bitrián A., Bayindir I., Hadi A. N., Wiesner A., Riedel S. (2022). Air-stable
aryl derivatives of pentafluoro­orthotellurate. Chem. Commun..

[135] Eder T., Buß F., Wilm L. F. B., Seidl M., Podewitz M., Dielmann F. (2022). Oxidative Fluorination
of Selenium
and Tellurium Compounds using a Thermally Stable Phosphonium SF_5_
^–^ Salt Accessible from SF_6_. Angew. Chem. Int. Ed..

[newref138] Wegener D., Limberg N., Bubenik M., Perez-Bitrian A., Wiesner A., Riedel S. (2025). An Aluminum‑Based Lewis Superacid
and Its Weakly Coordinating Anions Derived from an Organotellurium
Ligand. JACS Au.

[136] Wegener D., Pérez-Bitrián A., Limberg N., Wiesner A., Hoffmann K. F., Riedel S. (2024). A Highly Sterically
Encumbered Boron Lewis Acid Enabled by an Organotellurium-Based Ligand. Chem. Eur. J..

[137] Engelbrecht A., Sladky F. (1965). Salts of pentafluoro orthotelluric
acid. Inorg. Nucl. Chem. Lett..

[138] Mayer E., Sladky F. (1975). Infrared and Raman
Spectra of the
OTeF_5_
^–^ Anion and Evidence for Contact-Ion-Pair
Formation in the TeF_5_OAg-CH_3_CN System. Normal-Coordinate
Analysis of the OTeF_5_
^–^ and OSeF_5_
^–^ Ions. Inorg. Chem..

[139] Sawyer J. F., Schrobilgen G. J. (1982). Tris­[pentafluorotellurato­(VI)]­boron­(III). Acta Crystallogr. B Struct. Sci..

[140] Young D. E., Anderson L. R., Fox W. B. (1971). Perhaloalkyl
Hypochlorites.
V. Perfluoroalkyl Borate Esters from Reactions with Boron Trichloride. Inorg. Chem..

[141] Schack C. J., Christe K. O. (1984). An Improved Synthesis
of TeF_5_OF. Inorg. Chem..

[142] Huppmann P., Labischinski H., Lentz D., Pritzkow H., Seppelt K. (1982). Übergangsmetallderivate
mit dem Liganden −OTeF_5_. Z.
Anorg. Allg. Chem..

[143] Noirot M. D., Anderson O. P., Strauss S. H. (1987). A New Candidate
for the Least Coordinating Anion: Preparation and Characterization
of [TlOTeF_5_(mes)_2_]_2_ · mes (mes
= Mesitylene) and [Tl­(mes)_2_
^+^]​[B­(OTeF_5_)_4_
^–^]. Inorg.
Chem..

[144] Van Seggen D. M., Hurlburt P. K., Noirot M. D., Anderson O. P., Strauss S. H. (1992). Tetrakis­(pentafluoro­oxotellurato)­borate­(1−):
Coordinating Ability and Reactivity of a Very Large Weakly Coordinating
Anion. Inorg. Chem..

[145] Hurlburt P. K., Anderson O. P., Strauss S. H. (1992). Preparation
and
characterization of Tl­(1,2-C_2_H_4_Cl_2_)­B­(OTeF_5_)_4_: a metal complex of a least coordinating
solvent and a least coordinating anion. Can.
J. Chem..

[146] Hurlburt P. K., Anderson O. P., Strauss S. H. (1991). Ag­(CO)­B­(OTeF_5_)_4_: The First Isolable Silver Carbonyl. J. Am.
Chem. Soc..

[147] Hurlburt P. K., Rack J. J., Luck J. S., Dec S. F., Webb J. D., Anderson O. P., Strauss S. H. (1994). Nonclassical Metal
Carbonyls: [Ag­(CO)]^+^ and [Ag­(CO)_2_]^+^. J. Am. Chem. Soc..

[148] Hurlburt P. K., Rack J. J., Dec S. F., Anderson O. P., Strauss S. H. (1993). [Ag­(CO)_2_]​[B­(OTeF_5_)_4_]: The First Structurally Characterized M­(CO)_2_ Complex. Inorg. Chem..

[149] Koppe K., Bilir V., Frohn H.-J., Mercier H. P. A., Schrobilgen G. J. (2007). Syntheses, Solution Multi-NMR Characterization,
and Reactivities of [C_6_F_5_Xe]^+^ Salts
of Weakly Coordinating Borate Anions, [BY_4_]^−^ (Y = CF_3_, C_6_F_5_, CN, or OTeF_5_). Inorg. Chem..

[150] Moran M. D., Mercier H. P. A., Schrobilgen G. J. (2007). Synthesis
and Structural Characterization of C­(OTeF_5_)_4_ and a Comparative Structural Study of the Isoelectronic B­(OTeF_5_)_4_
^–^ anion. Inorg. Chem..

[151] Bui M., Hoffmann K. F., Braun T., Riedel S., Heinekamp C., Scheurell K., Scholz G., Stawski T. M., Emmerling F. (2023). An Amorphous
Teflate Doped Aluminium Chlorofluoride: A Solid Lewis-Superacid for
the Dehydrofluorination of Fluoroalkanes. ChemCatChem.

[152] Olah, G. A. Superacid Chemistry, 2nd ed; Wiley, 2009.

[153] Kotsyuda S., Wiesner A., Steinhauer S., Riedel S. (2021). Synthesis and Structural
Characterization of Tetraalkylammonium
Salts of the Weakly Coordinating Anion [Al­(OTeF_5_)_4_]^−^. Z. Anorg. Allg. Chem..

[154] Mann L., Hornberger E., Steinhauer S., Riedel S. (2018). Further Development
of Weakly Coordinating Cations:
Fluorinated Bis­(triarylphosphoranylidene)­iminium Salts. Chem. Eur. J..

[155] Fischer L., Wossidlo F., Frost D., Coles N. T., Steinhauer S., Riedel S., Müller C. (2021). One-step methylation
of aromatic phosphorus heterocycles: synthesis and crystallographic
characterization of a 1-methyl-phosphininium salt. Chem. Commun..

[156] Willrett J., Schmitt M., Zhuravlev V., Sellin M., Malinowski P. J., Krossing I. (2024). Synthesis and Characterization
of a Copper Dinitrogen Complex Supported by a Weakly Coordinating
Anion. Angew. Chem. Int. Ed..

[157] Armbruster C., Sellin M., Seiler M., Würz T., Oesten F., Schmucker M., Sterbak T., Fischer J., Radtke V., Hunger J., Krossing I. (2024). Pushing redox potentials
to highly positive values using inert fluorobenzenes and weakly coordinating
anions. Nat. Commun..

[158] Barthélemy A., Scherer H., Weller H., Krossing I. (2024). On the Synthesis
and Structure of ’Naked’ Ga­(I) and In­(I) Salts and the
Surprising Stability of Simple Ga­(I) and In­(I) Salts in the Coordinating
Solvents Ether and Acetonitrile. Chem. Eur.
J..

[159] Köring L., Stepen A., Birenheide B., Barth S., Leskov M., Schoch R., Krämer F., Breher F., Paradies J. (2023). Boron-Centered
Lewis Superacid through
Redox-Active Ligands: Application in C-F and S-F Bond Activation. Angew. Chem. Int. Ed..

[160] Bens T., Walter R. R. M., Beerhues J., Schmitt M., Krossing I., Sarkar B. (2023). The Best of Both Worlds: Combining
the Power of MICs and WCAs To Generate Stable and Crystalline Cr^I^-Tetracarbonyl Complexes with *π*-Accepting
Ligands. Chem. Eur. J..

[161] Brückner T., Fantuzzi F., Stennett T. E., Krummenacher I., Dewhurst R. D., Engels B., Braunschweig H. (2021). Isolation
of Neutral, Mono-, and Dicationic B_2_P_2_ Rings
by Diphosphorus Addition to a Boron-Boron Triple Bond. Angew. Chem. Int. Ed..

[162] Errulat D., Harriman K. L. M., Gálico D. A., Kitos A. A., Mansikkamäki A., Murugesu M. (2023). A trivalent 4f complex
with two bis-silylamide ligands displaying slow magnetic relaxation. Nature Chem..

[163] Martens A., Petersen O., Scherer H., Riddlestone I., Krossing I. (2018). From a (Pseudo) Aluminum Sesquihalide
Al_2_(Et)_3_(OR^F^)_3_ to Me_3_Si-Cl-Al­(OR^F^)_3_ (R^F^ = C­(CF_3_)_3_). Organometallics.

[164] Strauss S. H., Noirot M. D., Anderson O. P. (1986). Teflate (OTeF_5_
^–^) as a Unique Ligand for Metal Complexes:
Structure of [TlOTeF_5_(1,3,5-(CH_3_)_3_C_6_H_3_)_2_]_2_, a Thallium­(I)
Complex with Neutral Arene Ligands. Inorg. Chem..

[165] Strauss S. H., Noirot M. D., Anderson O. P. (1985). Preparation and
Characterization of Silver­(I) Teflate Complexes: Bridging OTeF_5_ Groups in the Solid State and in Solution. Inorg. Chem..

[166] Fraser G. W., Millar J. B. (1974). Reactions of Tellurium
Hexafluoride
with Alcohols: Preparation and Properties of Mono- and Di-alkoxotellurium­(VI)
Fluorides. J. Chem. Soc., Dalton Trans..

[167] Fraser G. W., Meikle G. D. (1977). Synthesis of Alkoxotellurium­(VI)
Fluorides. J. Chem. Soc., Dalton Trans..

[168] Clouston A., Peacock R. D., Fraser G. W. (1970). The Reaction
of
Tellurium Hexafluoride with Methyl Alcohol. J. Chem. Soc. D.

[169] Fraser G. W., Meikle G. D. (1979). The use of alkoxotellurium­(VI)
fluorides
as alkylating agents. J. Chem. Soc., Perkin
Trans. 1.

[170] Fraser G. W., Meikle G. D. (1975). Reaction of Tellurium
Hexafluoride
with Ethylene Glycol and Other Polyhydric Alcohols. J. Chem. Soc., Dalton Trans..

[171] Hämmerling S., Voßnacker P., Steinhauer S., Beckers H., Riedel S. (2020). Friedel-Crafts Type
Methylation with
Dimethylhalonium Salts. Chem. Eur. J..

[172] Shack C. J., Christe K. O. (1984). Reactions of Pentafluorotellurium
Hypohalites with Fluoroolefins. J. Fluorine
Chem..

[173] Schack C. J., Christe K. O. (1985). Bis-Pentafluorotelluriumoxide Fluorocarbons. J. Fluorine Chem..

[174] Schack C. J., Christe K. O. (1988). Pentafluorotelluriumoxide
Derivatives
of Fluorocarbons. J. Fluorine Chem..

[175] Schack C. J., Christe K. O. (1990). Reactions of TeF_5_OCl with
fluorocarbon iodides and synthesis of CF_3_OTeF_5_. J. Fluorine Chem..

[176] Schack, C. J. ; Christe, K. O. Synthesis of RfOTeF_5_ . US19860824822 19860131.

[177] Schack, C. J. ; Christe, K. O. Pentafluorotelluriumoxide fluorocarbons. US19830478589 19830324.

[178] Schack, C. J. ; Christe, K. O. Multi-(OTeF_5_)-substituted fluorocarbons. US19840617456 19840529.

[179] Ellwanger M. A., von Randow C., Steinhauer S., Zhou Y., Wiesner A., Beckers H., Braun T., Riedel S. (2018). Tuning the Lewis acidity of difluorido
gold­(III) complexes:
the synthesis of [AuClF_2_(SIMes)] and [AuF_2_(OTeF_5_)​(SIMes)]. Chem. Commun..

[180] Vij A., Wilson W. W., Vij V., Corley R. C., Tham F. S., Gerken M., Haiges R., Schneider S., Schroer T., Wagner R. I. (2004). Methyl Tin­(IV) Derivatives of HOTeF_5_ and HN­(SO_2_CF_3_)_2_: A Solution
Multinuclear NMR Study and the X-ray Crystal Structures of (CH_3_)_2_SnCl­(OTeF_5_) and [(CH_3_)_3_Sn­(H_2_O)_2_]​[N­(SO_2_CF_3_)_2_]. Inorg. Chem..

[181] Schack C.
J., Christe K. O. (1984). Synthesis
of bis-pentafluorotelluriumoxide
fluorocarbons. J. Fluorine Chem..

[182] Schack C. J., Christe K. O. (1988). Synthesis of pentafluoroseleniumoxide
fluorocarbons. J. Fluorine Chem..

[183] Schack C. J., Christe K. O. (1988). Pentafluorotelluriumoxide
derivatives
of fluorocarbons. J. Fluorine Chem..

[184] Thrasher J. S., Seppelt K. (1985). Nitrosyl-pentafluorotellurat­(VI). Z. Anorg. Allg. Chem..

[185] Strauss S. H., Abney K. D. (1984). *σ*- and *π*-Acidity of As­(OTeF_5_)_3_. Inorg. Chem..

[186] Zhou J., Liu L. L., Cao L. L., Stephan D. W. (2019). The Arene-Stabilized
η^5^-Pentamethylcyclopentadienyl Arsenic Dication [(η^5^-Cp*) As­(toluene)]^2+^. Angew.
Chem. Int. Ed..

[187] Collins M., Rao U., Schrobilgen G. (1985). ^75^Arsenic NMR Study of [As­(OTeF_5_)_6_]^−^ in Solution. J. Magn. Reson..

[188] Leitzke O., Sladky F. (1981). Darstellung von Antimon­(V)­tetrafluoridpentafluorotellurat­(VI),
SbF_4_OTeF_5_ und Antimon­(V)­trifluorid-bis­[pentafluorotellurat­(VI)],
SbF_3_(OTeF_5_)_2_. Z. Naturforsch. B.

[189] Van Seggen D. M., Hurlburt P. K., Anderson O. P., Strauss S. H. (1995). Weakly
Coordinating Anions M­(OTeF_5_)_6_
^–^ (M = Nb, Sb) and M­(OTeF_5_)_6_
^2–^ (M = Ti, Zr, Hf): Two-Step Synthesis, Characterization, Stability,
and Use in the Isolation of the Dihaloalkane Complex Cations Ag­(CH_2_Cl_2_)_3_
^+^, Ag­(CH_2_Br_2_)_3_
^+^, and *catena*-poly­[Ag­(1,2-C_2_H_4_Br_2_)_2_-*μ*-(1,2-C_2_H_4_Br_2_)-*Br:Br’*]^+^. Inorg. Chem..

[190] Cameron T. S., Krossing I., Passmore J. (2001). The first simple, donor-free
salt of the Sb­(OTeF_5_)_6_
^–^ anion:
synthesis, structure, characterization, and thermochemistry of Cs­[Sb­(OTeF_5_)_6_]. Inorg. Chem..

[191] Cameron T.
S., Decken A., Dionne I., Fang M., Krossing I., Passmore J. (2002). Approaching
the Gas-Phase Structures
of [AgS_8_]^+^ and [AgS_16_]^+^ in the Solid State. Chem. Eur. J..

[192] Aris D., Beck J., Decken A., Dionne I., Schmedt auf der Günne J., Hoffbauer W., Köchner T., Krossing I., Passmore J., Rivard E., Steden F., Wang X. (2011). Metastable Se_6_ as a ligand
for Ag^+^: from isolated molecular to polymeric 1D and 2D
structures. Dalton Trans..

[193] Cameron T. S., Dionne I., Krossing I., Passmore J. (2002). Reactions
directed towards Sb­(OTeF_5_)_6_
^–^ salts of new tellurium cations: formation, FT-Raman spectrum and
X-ray crystal structure of [Cl_3_Te-F-TeCl_3_]​[Sb­(OTeF_5_)_6_] containing the *μ*-fluoro-bis­[trichloro-tellurium­(IV)]
cation. Solid State Sci..

[194] Casteel W. J., Kolb P., LeBlond N., Mercier H. P. A., Schrobilgen G. J. (1996). Tetrachloro- and Tetrabromostibonium­(V)
Cations: Raman and ^19^F, ^121^Sb, and ^123^Sb NMR Spectroscopic Characterization and X-ray Crystal Structures
of SbCl_4_
^+^Sb­(OTeF_5_)_6_
^–^ and SbBr_4_
^+^Sb­(OTeF_5_)_6_
^–^. Inorg. Chem..

[195] Miller H.
B., Baird H. W., Bramlett C. L., Templeton W. K. (1972). Tetrachlorostibonium­(V)
undecafluorodiantimonate­(V); its crystal structure and action as an
aromatic chlorinating agent. J. Chem. Soc.,
Chem. Commun..

[196] Preiss H. (1972). Die Kristallstruktur der Verbindung
SbCl_2_F_3_. Z. Anorg. Allg.
Chem..

[197] Müller U. (1979). Die Kristallstruktur des Antimonfluoridchlorids
SbCl_4_
^+^[Sb_2_F_10,5_Cl_0,5_]^−^:The Crystal Structure of the Antimony
Fluoride
Chloride SbCl_4_
^+^[Sb_2_F_10,5_Cl_0,5_]^−^. Z. Naturforsch.
B.

[198] Mokrai R., Barrett J., Apperley D. C., Batsanov A. S., Benkő Z., Heift D. (2019). Weak Pnictogen Bond with Bismuth:
Experimental Evidence Based on Bi-P Through-Space Coupling. Chem. Eur. J..

[199] Campbell R., Robinson P. L. (1956). The Fluorination
of Tellurium. Ditellurium
Decafluoride and Tellurium Oxyfluorides. J.
Chem. Soc..

[200] Seppelt K. (1973). Schwingungsspektren der einfachsten
F_5_SeO-Derivate. Z. Anorg. Allg. Chem..

[201] Sanders J. C. P., Schrobilgen G. J. (1989). Krypton Bis­[pentafluoro-oxotellurate­(VI)],
Kr­(OTeF_5_)_2_, the First Example of a Kr-O Bond. J. Chem. Soc., Chem. Commun..

[202] Zylka P., Oberhammer H., Seppelt K. (1991). Gas-Phase Structures
of the Bis­(pentafluorochalcogen)­peroxides F_5_MO-OMF_5_ with M = S, Se and Te. J. Mol. Struct..

[203] Bürger H. (1968). Die Schwingungsspektren einiger F_5_TeOH-Derivate. Z. Anorg. Allg. Chem..

[204] Bladon P., Brown D. H., Crosbie K. D., Sharp D. W. A. (1970). The
nuclear magnetic resonance spectra of F_5_TeOH, F_5_TeOSO_2_Cl, F_5_TeOSO_2_F, (F_5_TeO)_2_SO_2_, and (F_5_Te)_2_O. Spectrochim. Acta, Part A.

[205] Hoppenheit R., Mews R. (1985). Thiazyl-pentafluoro­oxotellurat,
NSOTeF_5_. Chem. Ber..

[206] Mercier H. P. A., Sanders J. C. P., Schrobilgen G. J. (1995). The Te­(OTeF_5_)_5_
^–^ and FTe­(OTeF_5_)_4_
^–^ Anions: Synthesis, X-ray Structure Determinations,
and Raman Spectra of Te­(OTeF_5_)_4_ and N­(CH_3_)_4_
^+^Te­(OTeF_5_)_5_
^–^ and Solution Characterization of the FTe­(OTeF_5_)_4_
^–^ and Te­(OTeF_5_)_5_
^–^ Anions by ^19^F and ^125^Te NMR Spectroscopy. Inorg. Chem..

[207] Pritzkow H., Seppelt K. (1977). Crystal and molecular structure of
tellurium­(VI) tetrakis­[oxopentafluorotellurate­(VI)] difluoride. Inorg. Chem..

[208] Pritzkow H., Seppelt K. (1976). Kristallstruktur von
Te_5_O_4_F_22_. Angew.
Chem..

[209] Crawford R. A., Dudley F. B., Hedberg K. (1959). A Verification of the
Molecular Structure of Pentafluorosulfur Hypofluorite (SF_5_OF) by Electron Diffraction 1,2. J. Am. Chem.
Soc..

[210] Schack C. J., Christe K. O. (1982). New Syntheses of Pentafluorotellurium
Hypochlorite. J. Fluorine Chem..

[211] Gould D. E., Anderson L. R., Young D. E., Fox W. B. (1969). Perhaloalkyl
Hypochlorites and Pentafluorosulfur Hypochlorite. I. Preparation and
Properties. J. Am. Chem. Soc..

[212] Kornath A., Hartfeld N., Oberhammer H. (1997). Vibrational
Spectra and Gas-Phase Structure of Pentafluorosulfanyl Hypochlorite. Inorg. Chem..

[213] Hwang I.-C., Kuschel R., Seppelt K. (1997). Structures
of Bromine
Oxygen Compounds. Z. Anorg. Allg. Chem..

[214] Turowsky L., Seppelt K. (1991). Iodine Compounds with the -OTeF_5_ Ligand. Z. Anorg. Allg. Chem..

[215] Naumann, D. ; Habel, W. ; Reinelt, P. ; Renk, E. Ligandenaustauschreaktionen an Perfluoroorganohalogen-Verbindungen; Springer Fachmedien Wiesbaden GmbH, 1982. 10.1007/978-3-663-06757-3.

[216] Schack C. J., Christe K. O. (1990). Reactions of TeF_5_OCl with
Fluorocarbon Iodides and Synthesis of CF_3_OTeF_5_. J. Fluorine Chem..

[217] Jacob E., Lentz D., Seppelt K., Simon A. (1981). Edelgasverbindungen
mit dem Liganden -OTeF_5_. Z. Anorg.
Allg. Chem..

[218] Sladky F. (1969). FXeOTeF_5_: A Derivative of Xenon Difluoride. Angew. Chem. Int. Ed..

[219] Sladky F. (1969). Xe­(OTeF_5_)_2_,
Xenon Bis­(pentafluoro­orthotellurate). Angew. Chem. Int. Ed..

[220] Sladky F. (1970). Xenon­(II) Fluoride Pentafluoro­orthotellurate
as Fluoride Donor: [XeOTeF_5_]^+^ [AsF_6_]^−^. Angew. Chem. Int. Ed..

[221] Sladky, F. ; Schack, C. J. In Inorganic Syntheses, 24th ed.; Shreeve, J. M. , Ed.; John Wiley & Sons, 1986; pp 33–37. 10.1002/SERIES2146.

[222] Syvret R. G., Schrobilgen G. J. (1989). FXeOIOF_4_ and Xe­(OIOF_4_)_2_: Preparation and Study by ^129^Xe and ^19^F NMR
Spectroscopy and Raman Spectroscopy and NMR Characterization
of LXeOIOF_4_ (L = -OTeF_5_, -OSO_2_F). Inorg. Chem..

[223] Pötter B., Lentz D., Pritzkow H., Seppelt K. (1981). *gem*-Bis­(halooxy) Compounds from *cis*- and *trans*-Tetrfluordioxotellurium­(VI)
Acid, (HO)_2_TeF_4_. Angew.
Chem. Int. Ed..

[224] Turowsky L., Seppelt K. (1990). Polymeric Xenon Materials:
(-Xe-O-TeF_4_-O-)_n_ Crystal structure of HF·HO-TeF_4_-O-Xe^+^AsF_6_
^–^. Inorg. Chem..

[225] Keller N., Schrobilgen G. J. (1981). Preparation
of the XeOTeF_5_
^+^, FXeFXeOTeF_5_
^+^, XeF_2_·BrOF_2_
^+^, and XeOSO_2_F^+^ Cations and Their Study by ^129^Xe, ^125^Te and ^19^F Pulse Fourier Transform NMR and Raman
Spectroscopy. Inorg. Chem..

[226] Schrobilgen, G. J. Lewis Acid Properties of Fluorinated Noble Gas Cations. In Synthetic Fluorine Chemistry; Olah, G. A. , Chambers, R. D. , Surya Prakash, G. K. , Eds.; John Wiley & Sons, 1992; pp 1–30.

[227] Seppelt K. (1979). Recent Developments
in the Chemistry of Some Electronegative
Elements. Acc. Chem. Res..

[228] Lentz D., Seppelt K. (1978). Xenon Tetrakis­(pentafluoro­orthotellurate),
Xe­(OTeF_5_)_4_. Angew. Chem.
Int. Ed..

[229] Turowsky L., Seppelt K. (1992). Structures of Xenon Oxygen Compounds. Z. Anorg. Allg. Chem..

[230] Schumacher G. A., Schrobilgen G. J. (1984). Preparation
of O_2_XeF_2‑x_(OTeF_5_)_x_, OXeF_4‑y_(OTeF_5_)_y_ and XeF_4‑y_(OTeF_5_)_y_ (x = 0–2, y
= 0–4) and Study by ^129^Xe and ^19^F NMR
and Raman Spectroscopy: The Oxygen
Primary Isotopic Effect in the ^129^Xe NMR Spectra of XeO_2_F_2_ and XeOF_4_. Inorg. Chem..

[231] Syvret R. G., Mitchell K. M., Sanders J. C. P., Schrobilgen G. J. (1992). F_X_Xe­(OTeF_5_)_3‑*x*
_
^+^, O=XeF_
*x*
_(OTeF_5_)_3‑*x*
_
^+^ (*x* = 0–2), and
O_2_XeOTeF_5_
^+^ Cations: Their Preparation
and Characterization in Solution by ^129^Xe and ^19^F NMR Spectroscopy. Inorg. Chem..

[232] Paprica A., Sanders J., Schrobilgen G. J. (1989). Synthesis
and Characterization of XeOTeF_5_
^+^Sb­(OTeF_5_)_6_
^–^. J.
Fluorine Chem..

[233] DeBackere J. R., Mercier H. P. A., Schrobilgen G. J. (2014). Noble-gas
difluoride complexes of mercury­(II): the syntheses and structures
of Hg­(OTeF_5_)_2_·1.5NgF_2_ (Ng =
Xe, Kr) and Hg­(OTeF_5_)_2_. J. Am. Chem. Soc..

[234] Riedel S., Straka M., Kaupp M. (2005). Can Weakly
Coordinating
Anions Stabilize Mercury in Its Oxidation State +IV?. Chem. Eur. J..

[235] Colsman M. R., Newbound T. D., Marshall L. J., Noirot M. D., Miller M. M., Wulfsberg G. P., Frye J. S., Anderson O. P., Strauss S. H. (1990). Silver­(I) Complexes
of Dichloromethane and 1,2-Dichloroethane. J.
Am. Chem. Soc..

[236] Colsman M. R., Noirot M. D., Miller M. M., Anderson O. P., Strauss S. H. (1988). Lewis Basicity of the “Noncoordinating”
Common Solvent 1,2-Dichloroethane: Strong RCl → Ag Bonding
in AgOTeF_5_(1,2-C_2_H_4_Cl_2_). J. Am. Chem. Soc..

[237] Van Seggen D. M., Anderson O. P., Strauss S. H. (1992). A Chloroalkane-Silver
Complex with a Monodentate RCl → Ag­(I) Interaction. Inorg. Chem..

[238] Dudley F. B. (1963). Alternative Syntheses of Peroxodisulphuryl
Difluoride. J. Chem. Soc..

[newref242] Rohlan V. (2025). Theoretical analysis of the Lewis
acidity of transition
metal-based complexes: [Cu­(OTeF_5_)_3_], [Ag­(OTeF_5_)_3_] and [Au­(OTeF_5_)_3_]. Mol. Phys..

[239] Schröder K., Sladky F. (1980). Preparation and Properties
of TiCl_4‑n_(OTeF_5_)_n_ (n = 1–4),
Cs_2_[Ti­(OTeF_5_)_6_], and Ti­(OTeF_5_)_4_ · 2 POCl_3_. Chem. Ber..

[240] Turowsky L., Seppelt K. (1990). Molybdän- und Wolframverbindungen
mit dem Liganden −OTeF_5_. Z.
Anorg. Allg. Chem..

[241] Rack J. J., Moasser B., Gargulak J. D., Gladfelter W. L., Hochheimer H. D., Strauss S. H. (1994). Infrared and Manometric Evidence
for the Formation of the [Ag­(CO)_3_]^+^ Complex
Ion at High *P*
_CO_. J. Chem. Soc., Chem. Commun..

[242] Hurlburt, P. K. . Dissertation, Colorado State University, Fort Collins, CO, 1993.

[243] Newbound T. D., Colsman M. R., Miller M. M., Wulfsberg G. P., Anderson O. P., Strauss S. H. (1989). Dichloromethane Is a Coordinating
Solvent. J. Am. Chem. Soc..

[244] Yang L., Powell D. R., Houser R. P. (2007). Structural
variation
in copper­(I) complexes with pyridylmethylamide ligands: structural
analysis with a new four-coordinate geometry index, *τ*
_4_. Dalton Trans..

[245] DeBackere J. R., Mercier H. P. A., Schrobilgen G. J. (2015). Pentafluoro-oxotellurate­(VI)
Anions of Mercury­(II); Syntheses and Structures of [Hg­(OTeF_5_)_4_]^2–^, [Hg­(OTeF_5_)_5_]^3–^, [Hg_2_(OTeF_5_)_6_]^2–^, [Hg­(OTeF_5_)_4_]^2–^·Hg­(OTeF_5_)_2_, and [Hg_2_(OTeF_5_)_7_]^3–^·Hg­(OTeF_5_)_2_. Inorg. Chem..

[246] Addison A. W., Rao T. N., Reedijk J., van Rijn J., Verschoor G. C. (1984). Synthesis, Structure, and Spectroscopic
Properties
of Copper­(II) Compounds containing Nitrogen-Sulphur Donor Ligands;
the Crystal and Molecular Structure of Aqua­[1,7-bis­(*N*-methylbenzimidazol-2′-yl)-2,6-dithiaheptane]­copper­(II) Perchlorate. J. Chem. Soc., Dalton Trans..

[247] Schröder K., Sladky F. (1981). Molybdän­(VI)-fluorid-pentafluorotellurate­(VI)
und Molybdän­(VI)-oxid-fluorid-pentafluorotellurate­(VI): MoF_n_(OTeF_5_)_6‑n_ und MoOF_n_(OTeF_5_)_4‑n_. Z.
Anorg. Allg. Chem..

[248] Johnson D. A., Taylor J. C., Waugh A. B. (1980). Crystal and molecular
structure of tetra (tert-perfluoro butoxy) oxo Mo­(VI). J. Inorg. Nucl. Chem..

[249] Casteel W. J., MacLeod D. M., Mercier H. P. A., Schrobilgen G. J. (1996). Synthesis
and Characterization by ^19^F and ^125^Te NMR and
Raman Spectroscopy of *cis*-ReO_2_(OTeF_5_)_3_ and *cis*-ReO_2_(OTeF_5_)_4_
^–^, and X-ray Crystal Structure
of [N­(CH_3_)_4_
^+^]​[*cis*-ReO_2_(OTeF_5_)_4_
^–^]. Inorg. Chem..

[250] Turowsky L., Seppelt K. (1990). Rheniumverbindungen
mit dem Liganden
−OTeF_5_. Z. Anorg. Allg. Chem..

[251] Leitzke O., Sladky F. (1981). Wolfram­(VI)-fluorid-pentafluorotellurate­(VI):
WF_n_(OTeF_5_)_6‑n_. Z. Anorg. Allg. Chem..

[252] Wang X., Andrews L., Riedel S., Kaupp M. (2007). Mercury Is
a Transition Metal: The First Experimental Evidence for HgF_4_. Angew. Chem. Int. Ed..

[253] Kellett P. J., Pawlik M. J., Taylor L. F., Thompson R. G., Levstik M. A., Anderson O. P., Strauss S. H. (1989). Five- and
Six-Coordinate
High-Spin Iron­(III) Porphyrin Complexes with Teflate (OTeF_5_
^–^) Ligands. Inorg. Chem..

[254] Pawlik M. J., Miller P. K., Sullivan E. P., Levstik M. A., Almond D. A., Strauss S. H. (1988). Comparison of Variable-Temperature ^1^H NMR
Spectra of Five-Coordinate High-Spin (S = 5/2) Iron­(III)
Porphyrins and Chlorins. J. Am. Chem. Soc..

[255] Colsman M. R., Manning M. C., Anderson O. P., Strauss S. H. (1987). Preparation
and Characterization of Palladium­(II) and Platinum­(II) OTeF_5_ Complexes. Inorg. Chem..

[256] Buggey L. A., Hope E. G. (1995). A platinum­(II) phosphine
complex
containing the OTeF_5_
^–^ ligand. J. Fluorine Chem..

[257] Brewer S. A., Buggey L. A., Holloway J. H., Hope E. G. (1995). Reactions
of *cis*-[Os­(CO)_4_Me_2_] and *cis*-[Re­(CO)_4_(PPh_3_)­Me] with Anhydrous
HF and HOTeF_5_; Compatibiltiy of Phosphine, Alkyl and OTeF_5_
^–^ Ligands in Low-valent Transition-metal
Complexes. J. Chem. Soc., Dalton Trans..

[258] Hämmerling S., Mann L., Steinhauer S., Kuntze-Fechner M. W., Radius U., Riedel S. (2018). Investigation of Organonickel-Pentafluoro­orthotellurates. Z. Anorg. Allg. Chem..

[259] Hurlburt P. K., Kellett P. J., Anderson O. P., Strauss S. H. (1990). Nitrobenzene
as a Monodentate (*O*) and Bidentate (*O*, *O*’) Ligand: Synthesis and Structures of
Zn­(PhNO_2_)_n_(OTeF_5_)_2_ (n
= 2, 3). J. Chem. Soc., Chem. Commun..

[260] DeBackere J. R., Mercier H. P. A., Schrobilgen G. J. (2015). Thiazyl
Trifluoride (NSF_3_) Adducts and Imidodifluorosulfate (F_2_OSN-) Derivatives of Hg­(OTeF_5_)_2_. Inorg. Chem..

[261] Crossman M. C., Hope E. G., Saunders G. C. (1996). Cyclopentadienyl
metal teflate (OTeF_5_) complexes. J. Chem. Soc., Dalton Trans..

[262] Abney K. D., Long K. M., Anderson O. P., Strauss S. H. (1987). Preparation
and Properties of Metal Carbonyl Teflates, Including the Structure
and Reactivity of Mn­(CO)_5_(OTeF_5_). Inorg. Chem..

[263] Strauss S. H., Abney K. D., Long K. M., Anderson O. P. (1984). Low-Valent
Metal Teflates: Preparation and Characterization of Mn­(CO)_5_(OTeF_5_). Inorg. Chem..

[264] Buggey L. A., Hope E. G. (1996). Pentafluoro­oxotellurate­(VI)
acid as a protic acid. J. Fluorine Chem..

[265] Danopoulos A. A., Simler T., Braunstein P. (2019). N-Heterocyclic
Carbene Complexes of Copper, Nickel, and Cobalt. Chem. Rev..

[266] Templeton L. K., Templeton D. H., Bartlett N., Seppelt K. (1976). Crystal and
Molecular Structure of Uranium Hexakis­(oxopentafluorotellurate), U­(OTeF_5_)_6_. Inorg. Chem..

[267] Qian S., Qiu R., Tang J., Chen J., Liu P., Ao B. (2021). Theoretical
Assignment of Oxidation State of Uranium
in Binary, Ternary, and Quaternary Tellurides. J. Phys. Chem. C.

